# Supplementary material for: Effects of an automated digital brief prevention intervention targeting adolescents and young adults with risky alcohol and other substance use: study protocol for a randomised controlled trial
Source: BMJ Open. 2020 May 12;10(5):e034894. doi: 10.1136/bmjopen-2019-034894 (PMC7228518; doi:10.1136/bmjopen-2019-034894)
Supplement: Author's manuscript [file bmjopen-2019-034894.draft_revisions.pdf]

BMJ Open

BMJ Open is committed to open peer review. As part of this commitment we make the peer review history of every article we publish publicly available.

When an article is published we post the peer reviewers' comments and the authors' responses online. We also post the versions of the paper that were used during peer review. These are the versions that the peer review comments apply to.

The versions of the paper that follow are the versions that were submitted during the peer review process. They are not the versions of record or the final published versions. They should not be cited or distributed as the published version of this manuscript.

BMJ Open is an open access journal and the full, final, typeset and author-corrected version of record of the manuscript is available on our site with no access controls, subscription charges or pay-per-view fees (http://bmjopen.bmj.com).

If you have any questions on BMJ Open's open peer review process please email info.bmjopen@bmj.com

BMJ Open

A brief digital intervention targeting prevention of alcohol and other substance use among adolescents and young adults: Study protocol for a randomised controlled trial

Journal:	*BMJ Open*
Manuscript ID	bmjopen-2019-034894
Article Type:	Protocol
Date Submitted by the Author:	09-Oct-2019
Complete List of Authors:	Kvillemo, Pia; Karolinska Institutet, Department of Clinical Neuroscience, STAD, Centre for Psychiatry Research, Stockholm Health Care Services, Stockholm Region<br>Strandberg, Anna; Karolinska Institutet, Department of Clinical Neuroscience, STAD, Centre for Psychiatry Research, Stockholm Health Care Services, Stockholm Region<br>Gripenberg, Johanna; Karolinska Institutet, Department of Clinical Neuroscience, STAD, Centre for Psychiatry Research, Stockholm Health Care Services, Stockholm Region<br>Berman, Anne; Karolinska Institutet, Department of Clinical Neuroscience, Centre for Psychiatry Research, Stockholm Health Care Services, Stockholm Region<br>Skoglund, Charlotte; Karolinska Institutet, Department of Clinical Neuroscience, STAD, Centre for Psychiatry Research, Stockholm Health Care Services, Stockholm Region<br>Elgán, Tobias; Karolinska Institutet, Department of Clinical Neuroscience, STAD, Centre for Psychiatry Research, Stockholm Health Care Services, Stockholm Region
Keywords:	PSYCHIATRY, Substance misuse < PSYCHIATRY, Child & adolescent psychiatry < PSYCHIATRY, PREVENTIVE MEDICINE

SCHOLARONE™
Manuscripts

BMJ

I, the Submitting Author has the right to grant and does grant on behalf of all authors of the Work (as defined in the below author licence), an exclusive licence and/or a non-exclusive licence for contributions from authors who are: i) UK Crown employees; ii) where BMJ has agreed a CC-BY licence shall apply, and/or iii) in accordance with the terms applicable for US Federal Government officers or employees acting as part of their official duties; on a worldwide, perpetual, irrevocable, royalty-free basis to BMJ Publishing Group Ltd ("BMJ") its licensees and where the relevant Journal is co-owned by BMJ to the co-owners of the Journal, to publish the Work in this journal and any other BMJ products and to exploit all rights, as set out in our licence.

The Submitting Author accepts and understands that any supply made under these terms is made by BMJ to the Submitting Author unless you are acting as an employee on behalf of your employer or a postgraduate student of an affiliated institution which is paying any applicable article publishing charge ("APC") for Open Access articles. Where the Submitting Author wishes to make the Work available on an Open Access basis (and intends to pay the relevant APC), the terms of reuse of such Open Access shall be governed by a Creative Commons licence – details of these licences and which Creative Commons licence will apply to this Work are set out in our licence referred to above.

Other than as permitted in any relevant BMJ Author's Self Archiving Policies, I confirm this Work has not been accepted for publication elsewhere, is not being considered for publication elsewhere and does not duplicate material already published. I confirm all authors consent to publication of this Work and authorise the granting of this licence.

For peer review only

A brief digital intervention targeting prevention of alcohol and other substance use among adolescents and young adults: Study protocol for a randomised controlled trial

ABSTRACT

Introduction

Adolescence and young adulthood is a period in life when individuals may be especially vulnerable to harmful substance use. Several critical developmental processes are occurring in the brain, and substance use poses both short- and long-term risks with regard to mental health and social development. From a public health perspective, it is important to prevent or delay substance use to reduce individual risk and societal costs. Given the scarcity of effective interventions targeting substance use among adolescents and young adults, cost-effective and easily disseminated interventions are warranted. The study described in this protocol will test the effectiveness of a fully automated digital brief intervention aimed at reducing substance use in adolescents and young adults aged 15-25 years.

Methods and analysis

A two-arm randomised controlled trial (RCT) study design is applied to assess the effectiveness of a digital brief intervention. Baseline assessment at study entry and two follow-up assessments at three and six months will be carried out. The aim is to include 800 participants who screen positive for risky substance use. Recruitment, informed consent, randomisation, intervention, and follow-up will be implemented online. The primary outcome is reduction in alcohol use. Secondary outcomes concern other substance use, mental health, sexual risk behaviours, and perceived peer pressure. Moreover, the study involves analyses of potential moderators including perfectionism, openness to parents, help-seeking, and background variables. The trial is expected to expand the knowledge on digital preventive interventions for substance using adolescents and young adults.

Ethics and dissemination

The current study has been approved by the Swedish Ethical Review Authority (no. 2019-03249). Prior to inclusion, eligible participants must give informed consent online. Results will be disseminated in research journals, at conferences and via the media.

For peer review only - http://bmjopen.bmj.com/site/about/guidelines.xhtml

Registration

Date: 24/09/2019, ID: ISRCTN91048246.

Key words

Motivational Interviewing, Decisional balance, Substance use, Web-based intervention, Normative feedback.

ARTICLE SUMMARY

Strengths and limitations of this study

- A double-blind randomised controlled trial (RCT) is considered to be the most robust experimental design, enabling causal inferences, controlling for participant allocation bias and selection bias among recruited participants.
- An active control condition controls for performance bias and placebo effect.
- Analysis of several potential moderators will contribute to the understanding of possible effects.
- A convenience sample may reduce generalizability due to possible selection bias at recruitment.

INTRODUCTION

Substance use is a major public health concern causing individual suffering as well as societal costs [1-3]. Substance use in childhood and younger ages is particularly harmful since the brain is undergoing critical development during this time in life, which makes it more vulnerable to addictive substances [4, 5]. In adolescence, some individuals are especially prone to various risk behaviours and the initiation of substance use often occurs during this period [6]. Peers are increasingly important in teens' social life and the influence of substance using peers is a prominent risk factor for one's own substance use [7]. After initiation, the use of substances frequently increases during adolescence and young adulthood, posing a number of risks for the individual [2, 8, 9]. Early onset of substance use implies a risk of severe adverse effects on psychological and physiological development [2, 8, 9]. Hazardous use of alcohol, cannabis and other substances can lead to regular consumption and development of addiction chronic problematic consumption patterns that can significantly influence the developmental trajectory during the transition from childhood to adulthood [10]. More acute or short-term consequences include, e.g., problems of academic adjustment [11], accidents [12], and problems with the police or legal authorities [13]. Risk

For peer review only - http://bmjopen.bmj.com/site/about/guidelines.xhtml

behaviours, such as substance use, binge drinking, intoxication, and sexual risk behaviour often co-occur, and the use of lllicit substances, is often accompanied by alcohol consumption [1, 2, 11, 14-18]. The association between substance use and mental health problems is well established and bidirectional, sometimes as a result of self-medication but also as an increase in mental health problems due to substance use [19]. Poly-substance use, i.e., simultaneous use of different substances, has in previous research been associated with mental health problems, increased in magnitude and over time with the number of substances used [20].

Although alcohol use has declined among adolescents during recent years, some individuals drink more and binge drinking is still highly prevalent among young adults were the largest proportion of alcohol risk consumers is still found [21, 22]. Moreover, a high prevalence of substance use and poly-substance use among young people in Europe has been recognized in the EU Drug Strategy 2005–2012, which calls for action [23]. Also, established cannabis users among Swedish upper secondary class students tend to use cannabis more often than before, according to recent survey data [24]. In light of these circumstances, it is of utmost importance to target and tailor attractive preventive interventions for adolescents and young adults. One way to reach large groups of young people is to disseminate interventions via the Internet. In Sweden, 98 percent of the population has access to the Internet at home and virtually 100 percent of 16-25 year-olds use the Internet in mobile phones, tablets or computers [25]. Digital interventions have several advantages over traditional ones. They may reduce the stigma around risk behaviours, are accessible at any time and place, and can be cost-effective because minimal staff resources are used for implementation, especially if they are fully automated [26, 27]. Digital intervention may thus be particularly suitable for adolescents and young adults [28]. To date, digital prevention programs targeting alcohol and other substance use among adolescents and young adults that meet scientific evaluation criteria are scarce. An early example is eScreen.se, offering screening for alcohol and drug use with personalized feedback [29]. A more recent example is a fully automated brief motivational intervention, for substance-using 16-18 year-olds, which was tested in a randomised controlled trial (RCT) involving four European countries [30-32], where our research group was one of the partners. The intervention, named WISEteens, was based on Motivational Interviewing (MI) and demonstrated significant between-group effects for alcohol use, indicating that a targeted brief motivational intervention in a fully automated digital format can be effective to reduce drinking and lower barriers for existing substance use service in hazardous drinking adolescents [34].

Digital interventions generally offer adaptations of evidence based face-to-face interventions, e.g., MI. The effects of MI on several health related behaviours have been evaluated in a number of studies,

For peer review only - http://bmjopen.bmj.com/site/about/guidelines.xhtml

especially among adults [33]. Among adolescents, systematic reviews and meta-analyses on MI for various health-related behaviours show mixed results. A review of six studies of MI for reducing alcohol consumption in the emergency room setting suggested that MI was at least as effective as other brief interventions in the same setting, and suggested that MI could be even more effective than existing interventions [34]. Another review of 24 studies of different brief interventions for reducing alcohol consumption and alcohol-related problems among adolescents showed a significant effect that persisted up to one year compared to control interventions, with greater effects for MI. Effects were consistent over diverse settings and particularly effective components included decisional (juxtaposition of pros and cons of change) [35] and goal-setting [36]. Regarding MI for illicit drug use among adolescents, a review of 10 studies showed no effects on drug use behaviors; however, changes in attitudes towards drug use were found, which could be translated into intentions to change behaviors [35]. A scoping review concerning MI for reducing sexual risk behaviors among adolescents identified 29 unique studies with varying of designs, conceptualizations of MI and specific risk behaviours, making it difficult to generalize regarding outcomes but indicating the need of more research [37]. MI has most commonly been delivered as an individual face-to-face intervention, but has also been provided in other forms, e.g., via telephone or digitally, with various results [33, 38, 39]. Digital programs based on MI have demonstrated effect in the form of reduced alcohol consumption among young adults [40, 41] and a combination of screening with a short intervention has shown similar effects on alcohol consumption among adolescents [42]. Brief motivational interventions are empirically supported individual level interventions for reducing alcohol use [43, 44], which have also been digitally provided. Digital interventions can be provided with or without human guidance, i.e., more or less automated [45]. Previous research has demonstrated that even fully automated interventions can reduce alcohol-related problems for young people with risk consumption of alcohol up to 12 months after implementation [41, 46], and indicated potential effect on cannabis use in certain groups [47, 48]. Although various digital substance use prevention interventions have been developed and tested with promising results [30, 49], studies on the effects of fully automated digitally delivered and MI-based substance use prevention programs for adolescents and young adults are still few [49] and more well-designed studies are warranted in order to obtain evidence for potential effects.

Aim

The study described in this protocol is informed by builds on results previously obtained in a European study of a brief MI-based substance use prevention intervention (WISEteens), digitally delivered to adolescents in a fully automated form [30]. In this study, the effectiveness of a modified version of this intervention will be tested across a range of outcome measures among adolescents and young adults. We

For peer review only - http://bmjopen.bmj.com/site/about/guidelines.xhtml

aim to target individuals at an age when they commonly debut as alcohol or other substance users, recruiting 15-25 year-olds with hazardous alcohol or other substance use. The primary aim is reduction in frequency and quantity of substance use. The primary outcome is alcohol consumption at three-month follow-up. A secondary outcome includes reduction of alcohol consumption at six-month follow-up and reduction of other substance use at three and six-month follow-up, respectively. We hypothesize that participants in the intervention group will report reduced substance use (primarily alcohol consumption at three-month follow-up), with a larger effect size compared to an active control group receiving health information about various substances. A secondary aim involves analysis of intervention effects on mental health, sexual risk behaviours, perceived peer pressure, and analysis of a number of potential moderators including, perfectionism, openness to parents, help-seeking, and various background variables. The study will add to existing evidence regarding the effectiveness of digital interventions aimed at reducing alcohol and other substance use among young people.

METHODS AND ANALYSIS

In order to evaluate the effectiveness of the digital screening and brief motivational intervention, a double-blind two-arm RCT study design is planned with baseline assessment at study entry and follow-up assessment at three and six-months across a number of outcome variables. Figure 1 displays the trial design. We will also explore and test for moderator effects. To outline and report the current study we used SPIRITreporting guidelines [50].

Recruitment

The target group for the current study is adolescents and young adults aged 15-25 years with self-reported substance use, a capacity to understand the Swedish language, and access to the Internet. Based on a power calculation to detect a small effect size (Cohen's d=0.2) [51], the aim is to include 800 participants [52]. The participants will be recruited by offline marketing at youth health clinics and other health care units for youth, at upper secondary schools, and by social networks, using banner advertisements, which will result in a convenience sample from the general population [53]. To enhance participation and follow-up rates, an incentive in the form of a movie ticket for each completed follow-up assessment will be provided; i.e., up to 3 tickets. Up to three reminders will be sent out if participants do not respond to follow-up assessments.

For peer review only

Screening and informed consent

Potential participants will be guided to a digital landing page with optional screening by an adapted version of the screening tool for use of alcohol and other substances, CRAFFT (Car, Relax, Alone, Forget, Friends, Trouble) [54]. This six-item screening tool has demonstrated criterion validity and appropriateness for identifying substance-related problems among adolescents [55-57]. The primary eligibility criterion for participating in the study will be a score of 2 or more on the CRAFFT, since this value has shown satisfactory sensitivity for identifying substance use problems [54]. Those who score 2 or higher will be offered participation in the study and given information on confidentiality, voluntariness of participation, and human subject protections [53]. They will also be provided with contact information on suitable counseling service providers. Those who agree to participate will be guided to an online platform where they will be asked to give digital informed consent.

Assessment

All assessment will occur online. The selection of measures and associated instruments is based on the aim of the study and the theoretical base concerning factors influencing substance use, partly outlined above. While most of prior research on substance use related problems and mental health problems show that low socio-economic standard is a risk factor for substance use and related problems [58, 59], the current study is informed by recent research on mechanisms involved in adolescence alcohol- and other substance use, showing associations between internalizing symptoms and substance use among high school students in affluent areas [60, 61], as well as associations between achievement pressures (particularly excessive perfectionistic strivings), and isolation from parents (particularly low perceived closeness to mothers) [60, 62]. The latter finding is in line with previous research showing that a positive parent–child relationship is a protective factor related to lower likelihood of adolescence substance use [63, 64]. That said, the baseline assessment in the current study will cover behavioural and health-related questions for measuring various outcomes including alcohol and other substance use, mental health, sexual risk behaviours, and perceived peer pressure, as well as questions related to moderating variables including perfectionism, openness to parents, help-seeking due to mental health problems, and socio-demographic and personal characteristics, (i.e. sex, age, residence, occupation (student/not student), school performance (for students), and parents' education). Based on the previous European WISEteens study [30], where significant effects were found for alcohol consumption but not for other substance use, the primary outcome will be changes in alcohol use between baseline and both follow-up assessments.

For peer review only - http://bmjopen.bmj.com/site/about/guidelines.xhtml

Measures

Alcohol use will be measured using two instruments. One of them is the short version of the Alcohol Use Disorders Identification Test (AUDIT-C) [65], measuring frequency and amount of consumed alcohol and frequency of binge drinking, providing a widely used and valid index sum score for problematic alcohol use among adolescents [57, 66], the other is the Daily Drinking Questionnaire (DDQ) [67], measuring the quantity and frequency of alcohol use a typical week. Previous research has demonstrated that the DDQ is highly correlated with other measures of self-reported alcohol consumption [68]. Other substance use will be measured by the first three items of the Drug Use Disorder Identification Test (DUDIT) [69], assessing frequency of consumption of drugs other than alcohol, frequency of different types of drugs other than alcohol used at the same occasion (1 = never to 5 = four or more times a week), and number of occasions when drugs other than alcohol are consumed on a typical day of drug use (1 = zero to 5 = seven or more). The DUDIT has been found to be effective in screening for drug-related problems in clinically selected groups [70], and has proven useful in the context of public health surveys in Sweden [69, 71]. Mental health will be measured by The WHO-5 Well-Being Index, which has demonstrated validity both as a screening tool for depression and as an outcome measure in clinical trials [72]. This scale has been successfully applied across a wide range of study fields and translated into more than 30 languages, including Swedish [73], and found psychometrically sound. Changes in sexual risk behaviors will be measured by multiple choice questions on sexual behaviour under the influence of alcohol and/or other substances and unprotected sex, previously used in a survey among Swedish visitors at youth health clinics in Stockholm county [74]. Changes in perceived peer pressure will be measured by two items retrieved and adapted from the Peer pressure inventory [75]. Perfectionism will be measured by two subscales of the Frost Multidimensional Perfectionism Scale (FMPS) [76, 77], 2) openness to parents will be measured among participants who are up to 20 years old, by questions about disclosure from Stattin & Kerr [78-80], slightly modified into statements to be compatible with the digital format, and help- seeking due to mental health problems, will be measured by dichotomous questions regarding if, and to what healthcare provider, the participant has turned for help. Additionally, background data regarding sex, age, residence, occupation (student/not student), school performance (for students), and parents' education will be asked for, using multiple choice questions.

Randomisation

The study is double-blind, thus neither the participant or the researchers know which participant is allocated to the intervention or to the active control condition. After baseline assessment, the participants will be automatically randomised to one of the two study groups by a computer program using an

For peer review only - http://bmjopen.bmj.com/site/about/guidelines.xhtml

unrestricted randomisation protocol. Participants will then be informed about the name of their program and given access immediately.

The intervention

Participants allocated to the intervention group will initially be asked to give additional information on alcohol and other substance use and to state their body weight, in order to generate personalized feedback on substance use behaviour and to estimate alcohol blood concentration on a typical drinking occasion.

Theoretical background of the intervention

The intervention relies on Motivational Interviewing (MI) [81] and models of Social Influence [82, 83]. The goal of an MI approach in the present context is to enhance motivation to change by exploring and resolving ambivalence about substance-related behaviours. Important elements are personalized feedback about one's substance use behaviours in relation to normative comparisons, balancing of pros and cons of the target behaviour, and supporting self-efficacy. Personalized feedback, or tailored feedback, regarding risk-status and normative feedback relative to a relevant comparison group is perceived as more relevant for changing behavior than more general information [53]. Normative feedback include information about how a specific reference group actually consumes substances, in order to correct participants' "inflated perception" [43], and has been proven effective for reduction of alcohol consumption in young adults in previous meta-analyses [84, 85]. For adolescents and young adults, a normative feedback approach may be particularly appealing, assuming they are curious about how their substance consumption compares to their peers [86]. Likewise, an important element of the intervention is a focus on substance-related social norms and training on how to avoid social high-risk situations and how to resist peer pressure; i.e., raising refusal self-efficacy [87]. In line with the social influence hypothesis, which states that a prominent risk factor for substance use in adolescence is the influence of substance using peers [7], targeting peer pressure in brief digital interventions has been an important element for reducing alcohol use among adolescents [88].

Description of the intervention content

The intervention is interactive, digitally delivered in a fully automated form, and requires approximately 20 minutes to complete. Tailored feedback is given to the participants based on their responses to previous assessment and suggestions on how to respond to this feedback are provided. This interactivity imitates a face-to face "dialogue" with techniques from MI such as an empathic approach, rolling with resistance, aiming at creating a dissonance between actual and desired behaviour, raising self-efficacy, and at the same time avoiding argumentation [39]. The intervention consists of three main components outlined below, and additional health related information.

For peer review only

Personalized feedback

The personalized feedback includes an estimation of the participants' blood-alcohol-concentration (BAC) and information on the associated risks concerning the participants' heaviest drinking episode during the past 30 days. The value will be based on a measure of Peak Drinking Quantity [89] and estimated using the Widmark-formula, which takes into account weight and gender [90]. The participants will receive graphed feedback regarding number of standard alcohol units per week that they think their peers consume (descriptive norms), as well as the participant's individual levels of consumption in relation to comparative data (actual drinking levels) from a reference group. Comparative data (AUDIT-C scores) will be taken from alcohol prevalence estimates found in a nationally representative sample of 16-25 year-olds undertaken by the Public Health Agency of Sweden. Comparative feedback will be available for drinking but not for substances other than alcohol.

Interactive MI-based exercises

The exercises provided in the current intervention build on the assumption that participants may hold certain levels of ambivalence about their current substance use, and that if they are willing to make a change they may not know how to, or may not be confident that they are able to do so [81].Therefore, the intervention uses importance and confidence rulers with a short summary and feedback to encourage participants to reflect on personal reasons for change and explore personal strengths and ability to change. Furthermore, the program provides a decisional balance to pick up and graphically illustrate potential levels of ambivalence by offering the participants a list of possible pros and cons regarding the decision to change their current substance use [39, 91]. Participants are instructed to choose statements that apply to them and are presented with the resulting balance sheet of their personal comparative potential gains and losses.

Practical advice

The intervention focuses on raising self-efficacy for being able to avoiding drinking in social situations, if desirable [88]. The participant will be asked to select three among 12 provided drinking situations that they consider most tempting and rank them (the situations are adapted from the adolescent version of the Drinking Refusal Self-efficacy Questionnaire (DRSEQ-RA) [92]. According to the selection, a number of strategies will be offered for each of the selected drinking situations to provide participants with a tool kit necessary for engaging in and maintaining their behavioural goal.

Health related information connected to substance use

Finally, the intervention program includes health related information associated with substance use. The information is provided optionally throughout the program behind "read more" buttons and also at the end

For peer review only - http://bmjopen.bmj.com/site/about/guidelines.xhtml

of the program. The information contains statements regarding risks connected to substance use that the participant can reflect on, as well as optional links for more information. There are also two FAQ-sections about alcohol and cannabis, respectively, as well as information about how different substances may affect the individual, both physically and mentally. Finally, the information also contains cases, were the participant can read about some typical young persons who have used substances, why they used it and what negative effects they have noted, and why they chose to stop using it

The control condition

The control group will receive the same general health related information as those in the intervention group, i.e., the additional information which is connected to the intervention program.

Statistical analysis

Randomisation-checks of baseline variables regarding alcohol and other substances consumption and psychological state will be conducted using multivariate analyses of variance MANOVA. To test the effectiveness of the intervention, we will assess whether participants in the intervention group report decreased substance use, sexual risk behaviours, perceived peer-pressure, and improved their mental health after three and six months, respectively, compared to participants in the active control group. Data analyses will consist of comparing outcome measurements with regards to within-group and between-group differences according to the per protocol as well as the intention-to-treat (ITT) principle, accounting for all included participants regardless of whether or not they completed follow-up assessments. The main analysis of effectiveness will use mixed effects regression models, which can be applied to both continuous and categorical outcomes and also non-normally distributed outcomes. Moreover, mixed-effects regression models are robust to missing data in longitudinal studies [93]. In the current study we assume a quite large dropout rate with data missing at random (MAR), which can be handled using mixed effects regression models. Separate models will be run to test each outcome, i.e., alcohol use, other substance use, mental health, sexual risk behaviours and perceived peer pressure. Effect sizes will be calculated separately for the two measures used for the primary outcome variable (AUDIT-C and DDQ). Potential moderators, including perfectionism, openness to parents, help-seeking due to mental health problems, and socio-demographic and personal characteristics, (i.e. sex, age, residence, occupation (student/not student), school performance (for students), and parents' education) will serve as covariates in analyses connected to the secondary aim of the study.

Patient and public involvement statement

Patients and the public were not involved in the design and planning of the study.

For peer review only - http://bmjopen.bmj.com/site/about/guidelines.xhtml

ETHICS AND DISSEMINATION

The current study has been approved by the Swedish Ethical Review Authority (no. 2019-03249) and registered 24/09/2019, pre-result, on IRCTN, ID: ISRCTN91048246. Any important protocol modifications will be reported to IRCTN. For inclusion, all participants must give informed consent online prior to participation in the study. In order to use the web-based intervention program, the participants will need to state a username (which may be fictitious or a pseudonym) and an email address. The email address is necessary for the registration process and for sending one (new) password to use when logging in to the program. Regarding the actual study of the intervention program, all data collection will be done without collecting personal identification information, such as 10-digit personal identification numbers assigned to all residents in Sweden or participants' names, only personal e-mail addresses. The e-mail address will be used to connect data from the baseline measurement to the follow-up measurements. At a later stage, the raw data file will be unidentified and each person assigned a number instead of the e-mail address. The research group is responsible for ensuring that code lists and the data are kept safe in line with routines for handling and storing research data at Karolinka Institutet. All data is handled confidentially and will not be forwarded to third parties. Participating in the present study means that the participants need to reflect on their alcohol and other substance consumption. In addition, questions will be asked concerning personal circumstances, including the participants' mental health and family relationships. These issues may be perceived as somewhat unpleasant. However, in the information that potential participants receive, they are informed that participation is voluntary and that they at any time can interrupt their participation without explaining why. In addition, there will be reference to other types of official support (web pages and telephone numbers), if the advertisement or participation leads to concern about own substance use or related problems. Any issues brought up of the participants during the study will be documented and handled properly. Moreover, the research team includes professionals, such as a psychiatrist and a nurse, with possibility to refer participants to health care clinics if needed. The project's basic hypothesis is that the intervention will have positive effects, with regard to alcohol and other substance consumption among young people with risk use, and potential and benefit may include decreased or ceased risk behaviours. Overall, the benefit for the research persons is considered to exceed any risk of discomfort. Results will be disseminated in scientific peer reviewed journals, at conferences and via the media.

For peer review only - http://bmjopen.bmj.com/site/about/guidelines.xhtml

DISCUSSION OF STRENGTHS AND LIMITATIONS

The current study has a number of strengths. The protocol describes a two-arm double-blind RCT, considered to be the most robust experimental design controlling for participant allocation bias, selection bias, performance bias and placebo effect, and with the possibility to make causal inferences. Moreover, an active control condition controls for performance bias and placebo effect. The collection and analysis of information on potential moderators, allowing for control of these factors, facilitates the understanding of the possible effects. Thus, the present study will contribute to the literature on digital substance use prevention interventions among adolescens and young adults in several ways. The choice to study a digital intervention can from a public health perspective be regarded as positive since digital brief interventions have several advantages over face-to-face approaches, e.g., the reduction of stigma around help-seeking for substance use and the way they can be easily disseminated to large groups of people and thus be cost-effective. Moreover, digital interventions have previously proven effective in addressing alcohol and other substance use in the general population and some studies have shown promising results also among adolescents and young adults [94, 95]. The current intervention is well grounded in theory and incorporates elements of MI and social influence theory that has been shown to be effective in reducing problematic substance use in prior research [39]. Importantly, there are some limitations to the current study. One limitation concerns selection bias and thereby external validity, as recruitment requires either that individuals click on our ads at social media to be considered for inclusion, or that they attend upper secondary schools, or have been in contact with existing clinics, aware of the opportunity to participate in the study. Thus, our participants may have certain personality traits, or are especially prone to help-seeking help compared to a broader audience. The exclusion of people not understanding Swedish is also a limitation with regard to generalizability.

STATEMENTS

Author contributions

PK, AKS, JG, and THE obtained funding for the study. PK, THE, and CS further developed/modified the intervention. THE designed the study with contribution from PK, AKS, JG, AHB, and, CS. PK wrote this paper. All authors commented on successive manuscript drafts and approved the final version of the manuscript.

Funding statement

The work was supported by the National Public Health Agency of Sweden (grant no. 02299-2016-6.2) and the Alcohol Research Council of the Swedish Alcohol Retailing Monopoly (grant no. 2018-0016). Author

For peer review only

AHB was supported by Swedish Research Council (grant no. K2012-61P-22131-01-6). The funding bodies had no role in study design, data collection, analysis, data interpretation or writing manuscripts.

Data statement

Collected data will be available from the Centre for Psychiatry Research, a collaboration between the Karolinska Institute and Region Stockholm, but restrictions apply to their availability, as they were used under ethical permission for the current study, and so are not publicly available. However, data are available from the authors upon reasonable request and with permission from the Centre for Psychiatry Research.

Competing interests

The authors declare no competing interests.

Acknowledgments

The authors would like to thank Dr. Silke Diestelkamp and Dr. Christiane Baldus for invaluable help in the process of building the Swedish version of the WISEteens program.

For peer review only

REFERENCES

1. Dawson DA, Goldstein RB, Chou SP, Ruan WJ, Grant BF: **Age at first drink and the first incidence of adult-onset DSM-IV alcohol use disorders**. *Alcoholism Clinical and Experimental Research* 2008, **32**(12):2149-2160.
2. Hibell B, Guttormsson U, Ahlström S, Balakireva O, Bjarnason T, Kokkevi A, Kraus L: **The 2007 ESPAD Report: Substance use among students in 35 European countries**. In: *ESPAD, The European School Survey Project on Alcohol and Other Drugs.* Stockholm, Sweden; 2009.
3. Anderson P: **Global alcohol policy and the alcohol industry**. *Curr Opin Psychiatry* 2009, **22**(3):253-257.
4. Chambers RA, Taylor JR, Potenza MN: **Developmental neurocircuitry of motivation in adolescence: a critical period of addiction vulnerability**. *The American journal of psychiatry* 2003, **160**(6):1041-1052.

5. Barron S, White A, Swartzwelder HS, Bell RL, Rodd ZA, Slawecki CJ, Ehlers CL, Levin ED, Rezvani AH, Spear LP: **Adolescent vulnerabilities to chronic alcohol or nicotine exposure: findings from rodent models**. *Alcohol Clin Exp Res* 2005, **29**(9):1720-1725.
6. Balogh KN, Mayes LC, Potenza MN: **Risk-taking and decision-making in youth: relationships to addiction vulnerability**. *Journal of behavioral addictions* 2013, **2**(1).
7. Oetting ER, Beauvais F: **Peer cluster theory: Drugs and the adolescent**. *Journal of Counseling & Development* 1986, **65**(1):17-22.
8. Arria AM, Caldeira KM, Bugbee BA, Vincent KB, O'Grady KE: **Marijuana use trajectories during college predict health outcomes nine years post-matriculation**. *Drug and alcohol dependence* 2016, **159**:158-165.
9. Van Der Vorst H, Vermulst AA, Meeus WH, Dekovic M, Engels RC: **Identification and prediction of drinking trajectories in early and mid-adolescence**. *Journal of clinical child and adolescent psychology : the official journal for the Society of Clinical Child and Adolescent Psychology, American Psychological Association, Division 53* 2009, **38**(3):329-341.
10. R M, E K, P M, V R, S S: **Impact of alcohol consumption on young people: A systematic review of published reviews**. Newcastle: Newcastle University; 2009.
11. Hicks RD, Bemis Batzer G, Bemis Batzer W, Imai WK: **Psychiatric, Developmental, and Adolescent Medicine Issues in Adolescent Substance Use and Abuse**. *Adolesc Med* 1993, **4**(2):453-468.
12. White V, Azar D, Faulkner A, Coomber K, Durkin S, Livingston M, Chikritzhs T, Room R, Wakefield M: **Adolescents' alcohol use and strength of policy relating to youth access, trading hours and driving under the influence: findings from Australia**. *Addiction* 2018, **113**(6):1030-1042.
13. Flory K, Lynam D, Milich R, Leukefeld C, Clayton R: **Early adolescent through young adult alcohol and marijuana use trajectories: early predictors, young adult outcomes, and predictive utility**. *Development and psychopathology* 2004, **16**(1):193-213.
14. Nilses C, Blom H, Heimer G, Danielsson I: **40 procent av gymnasieelever riskbrukar alkohol**. *Läkartidningen* 2011, **34**(108):1156-1559.
15. Bonomo Y, Coffey C, Wolfe R, Lynskey M, Bowes G, Patton G: **Adverse outcomes of alcohol use in adolescents**. *Addiction* 2001, **96**(10):1485-1496.
16. Guttmannova K, Hill KG, Bailey JA, Lee JO, Hartigan LA, Hawkins JD, Catalano RF: **Examining explanatory mechanisms of the effects of early alcohol use on young adult alcohol dependence**. *J Stud Alcohol Drugs* 2012, **73**(3):379-390.
17. Miller JW, Naimi TS, Brewer RD, Jones SE: **Binge drinking and associated health risk behaviors among high school students**. *Pediatrics* 2007, **119**(1):76-85.
18. Chick CF, Reyna VF: **A fuzzy trace theory of adolescent risk taking: Beyond self-control and sensation seeking**. In: *The Adolescent Brain* edn. Edited by V. Reyna SC, M. Dougherty, & J. Confrey (Eds.): Washington, DC: American Psychological Association.; 2012.
19. Hodgins S, Tengström A, Bylin S, Göranson M, Hagen L, Janson M, Larsson A, Lundgren-Andersson C, Lundmark C, Norell E *et al*: **Consulting for substance abuse: Mental disorders among adolescents and their parents**. *Nordic Journal of Psychiatry* 2007, **61**(5):379-386.
20. Burdzovic Andreas J, Lauritzen G, Nordfjærn T: **Co-occurrence between mental distress and poly-drug use: A ten year prospective study of patients from substance abuse treatment**. *Addict Behav* 2015, **48**:71-78.
21. Guttormsson U, Gröndal M: **Befolkningens självrapporterade alkoholvanor 2004-2017** In. Stockholm: CAN; 2018.
22. Lees B, Mewton L, Stapinski L, Squeglia LM, Rae C, Teesson M: **Binge drinking in young people: protocol for a systematic review of neuropsychological, neurophysiological and neuroimaging studies**. *BMJ Open* 2018, **8**(7):e023629.
23. **European Union Drugs Strategy 2013–2020**. Brussels; 2013.

For peer review only - http://bmjopen.bmj.com/site/about/guidelines.xhtml

24. Zetterqvist ME: **Skolelevers drogvanor 2018**. Stockholm: Centralförbundet för alkohol- och narkotikaupplysning (CAN); 2018.
25. **Svenskarna och internet 2018 (The Swedes and the Internet 2018) (In Swedish)** [https://2018.svenskarnaochinternet.se/allmant-om-internetutvecklingen/]
26. Murray E: **Web-Based Interventions for Behavior Change and Self-Management: Potential, Pitfalls, and Progress**. *Med 20* 2012, **1**(2):e3.
27. Riper H, Blankers M, Hadiwijaya H, Cunningham J, Clarke S, Wiers R, Ebert D, Cuijpers P: **Effectiveness of Guided and Unguided Low-Intensity Internet Interventions for Adult Alcohol Misuse: A Meta-Analysis**. *PLOS ONE* 2014, **9**(6):e99912.
28. Spijkerman R, Roek AEM, Vermulst A, Lemmers L, Huiberts A, Engels CMER: **Effectiveness of a Web-Based Brief Alcohol Intervention and Added Value of Normative Feedback in Reducing Underage Drinking: A Randomized Controlled Trial**. *J Med Internet Res* 2010, **12**(5):e65.
29. Sinadinovic K, Berman AH, Hasson D, Wennberg P: **Internet-based assessment and self-monitoring of problematic alcohol and drug use**. *Addictive behaviors* 2010, **35**(5):464-470.
30. Arnaud N, Baldus C, Elgan TH, De Paepe N, Tonnesen H, Csemy L, Thomasius R: **Effectiveness of a Web-Based Screening and Fully Automated Brief Motivational Intervention for Adolescent Substance Use: A Randomized Controlled Trial**. *J Med Internet Res* 2016, **18**(5):e103.
31. Arnaud N, Bröning S, Drechsel M, Thomasius R, Baldus C: **Web-based screening and brief intervention for poly-drug use among teenagers: study protocol of a multicentre two-arm randomized controlled trial**. *BMC Public Health* 2012, **12**:826.
32. Arnaud N, Baldus C, Elgán TH, Tønnesen H, Paepe ND, Csemy L, Thomasius R: **Moderators of Outcome in a Web-Based Substance Use Intervention for Adolescents**. *SUCHT* 2015, **61**(6):377-387.
33. Frost H, Campbell P, Maxwell M, O'Carroll RE, Dombrowski SU, Williams B, Cheyne H, Coles E, Pollock A: **Effectiveness of Motivational Interviewing on adult behaviour change in health and social care settings: A systematic review of reviews**. *PloS one* 2018, **13**(10):e0204890-e0204890.
34. Kohler S, Hofmann A: **Can motivational interviewing in emergency care reduce alcohol consumption in young people? A systematic review and meta-analysis**. *Alcohol Alcohol* 2015, **50**(2):107-117.
35. Li L, Zhu S, Tse N, Tse S, Wong P: **Effectiveness of motivational interviewing to reduce illicit drug use in adolescents: a systematic review and meta-analysis**. *Addiction* 2016, **111**(5):795-805.
36. Tanner-Smith EE, Lipsey MW: **Brief alcohol interventions for adolescents and young adults: a systematic review and meta-analysis**. *Journal of substance abuse treatment* 2015, **51**:1-18.
37. Bahner J, Stenqvist K: **Motivational Interviewing as Evidence-Based Practice? An Example from Sexual Risk Reduction Interventions Targeting Adolescents and Young Adults**. *Sexuality Research and Social Policy* 2019.
38. Gates P, Norberg M, Copeland J, Digiusto E: **Randomized controlled trial of a novel cannabis use intervention delivered by telephone**. *Addiction (Abingdon, England)* 2012, **107**.
39. Berman AH, Beckman M, Lindqvist H: **Motivational Interviewing interventions**. In: *Handbook of Behavior Change.* Edited by Hagger MS, Cameron LD, Hamilton K, N. Hankonen N, Lintunen T. New York, NY: Cambridge University Press. In press; 2019.
40. Bewick BM, Trusler K, Barkham M, Hill AJ, Cahill J, Mulhern B: **The effectiveness of web-based interventions designed to decrease alcohol consumption — A systematic review**. *Preventive Medicine* 2008, **47**(1):17-26.
41. White A, Kavanagh D, Stallman H, Klein B, Kay-Lambkin F, Proudfoot J, Drennan J, Connor J, Baker A, Hines E *et al*: **Online Alcohol Interventions: A Systematic Review**. *J Med Internet Res* 2010, **12**(5):e62.

For peer review only

42. Toumbourou JW, Stockwell T, Neighbors C, Marlatt GA, Sturge J, Rehm J: **Interventions to reduce harm associated with adolescent substance use**. *Lancet (London, England)* 2007, **369**(9570):1391-1401.
43. Wood MD, Fairlie AM, Fernandez AC, Borsari B, Capone C, Laforge R, Carmona-Barros R: **Brief Motivational and Parent Interventions for College Students: A Randomized Factorial Study**. *J Consult Clin Psych* 2010, **78**(3):349-361.
44. Natarajan M, Kaner E: **Brief alcohol interventions in young people: evidence and applicability. I E. Gilvarry & P. McArdle (redaktörer), Alcohol, drugs and young people: clinical approaches**. Newcastle upon Tyne: Mac Keith Press; 2007.
45. Cavanagh K, Millings A: **(Inter)personal Computing: The Role of the Therapeutic Relationship in E-mental Health**. *Journal of Contemporary Psychotherapy* 2013, **43**(4):197-206.
46. Webb TL, Joseph J, Yardley L, Michie S: **Using the internet to promote health behavior change: a systematic review and meta-analysis of the impact of theoretical basis, use of behavior change techniques, and mode of delivery on efficacy**. *J Med Internet Res* 2010, **12**(1):e4.
47. Lee CM, Neighbors C, Kilmer JR, Larimer ME: **A brief, web-based personalized feedback selective intervention for college student marijuana use: a randomized clinical trial**. *Psychol Addict Behav* 2010, **24**(2):265-273.
48. Rooke S, Copeland J, Norberg M, Hine D, McCambridge J: **Effectiveness of a self-guided web-based cannabis treatment program: randomized controlled trial**. *Journal of medical Internet research* 2013, **15**(2):e26-e26.
49. Tonnesen H, H S, Pedersen B: **Review: Web-based brief interventions for young adolescent alcohol and drug abusers - a systematic review**, vol. 3; 2013.
50. Chan A-W, Tetzlaff JM, Altman DG, Laupacis A, Gøtzsche PC, Krleža-Jerić K, Hróbjartsson A, Mann H, Dickersin K, Berlin JA *et al*: **SPIRIT 2013 statement: defining standard protocol items for clinical trials**. *Ann Intern Med* 2013, **158**(3):200-207.
51. Cohen J: **A power primer**. *Psychological bulletin* 1992, **112**(1):155-159.
52. Riper H, Hoogendoorn A, Cuijpers P, Karyotaki E, Boumparis N, Mira A, Andersson G, Berman AH, Bertholet N, Bischof G *et al*: **Effectiveness and treatment moderators of internet interventions for adult problem drinking: An individual patient data meta-analysis of 19 randomised controlled trials**. *PLOS Medicine* 2018, **15**(12):e1002714.
53. Voogt CV, Poelen EA, Kleinjan M, Lemmers LA, Engels RC: **The effectiveness of the 'what do you drink' web-based brief alcohol intervention in reducing heavy drinking among students: a two-arm parallel group randomized controlled trial**. *Alcohol Alcohol* 2013, **48**(3):312-321.
54. Knight JR, Shrier LA, Bravender TD, Farrell M, Vander Bilt J, Shaffer HJ: **A new brief screen for adolescent substance abuse**. *Arch Pediatr Adolesc Med* 1999, **153**(6):591-596.
55. Levy S, Sherritt L, Harris SK, Gates EC, Holder DW, Kulig JW, Knight JR: **Test-retest reliability of adolescents' self-report of substance use**. *Alcohol Clin Exp Res* 2004, **28**(8):1236-1241.
56. Knight JR, Sherritt L, Harris SK, Gates EC, Chang G: **Validity of brief alcohol screening tests among adolescents: a comparison of the AUDIT, POSIT, CAGE, and CRAFFT**. *Alcohol Clin Exp Res* 2003, **27**(1):67-73.
57. Kallmen H, Berman AH, Jayaram-Lindstrom N, Hammarberg A, Elgan TH: **Psychometric Properties of the AUDIT, AUDIT-C, CRAFFT and ASSIST-Y among Swedish Adolescents**. *European addiction research* 2019, **25**(2):68-77.
58. Swift W, Coffey C, Carlin JB, Degenhardt L, Patton GC: **Adolescent cannabis users at 24 years: trajectories to regular weekly use and dependence in young adulthood**. *Addiction* 2008, **103**(8):1361-1370.
59. Von Sydow K, Lieb R, Pfister H, Hofler M, H.U W: **What predicts incident use of cannabis and progression to abuse and dependence? A 4-year prospective examination of risk factors**

For peer review only - http://bmjopen.bmj.com/site/about/guidelines.xhtml

in a community sample of adolescents and young adults. *Drug and Alcohol Dependence* 2002, **68**:49–64.

60. Låftman SB, Almquist YB, Östberg V: **Students' accounts of school-performance stress: a qualitative analysis of a high-achieving setting in Stockholm, Sweden**. *Journal of Youth Studies* 2013, **16**(7):932-949.
61. Hiltunen L: **Lagom perfekt. Erfarenheter av ohälsa bland unga tjejer och killar**. Växjö: Linnéuniversitetet; 2017.
62. Luthar SS, Becker BE: **Privileged but pressured? A study of affluent youth**. *Child development* 2002, **73**(5):1593-1610.
63. Ashby Wills T, Yaeger AM: **Family Factors and Adolescent Substance Use: Models and Mechanisms**. *Current Directions in Psychological Science* 2003, **12**(6):222-226.
64. Wills TA, Cleary SD: **How are social support effects mediated? A test with parental support and adolescent substance use**. *Journal of Personality and Social Psychology* 1996, **71**(5):937-952.
65. Saunders JB, Aasland OG, Babor TF, de la Fuente JR, Grant M: **Development of the Alcohol Use Disorders Identification Test (AUDIT): WHO Collaborative Project on Early Detection of Persons with Harmful Alcohol Consumption--II**. *Addiction* 1993, **88**(6):791-804.
66. Rumpf HJ, Wohlert T, Freyer-Adam J, Grothues J, Bischof G: **Screening questionnaires for problem drinking in adolescents: performance of AUDIT, AUDIT-C, CRAFFT and POSIT**. *European addiction research* 2013, **19**(3):121-127.
67. Collins RL, Parks GA, Marlatt GA: **Social determinants of alcohol consumption: the effects of social interaction and model status on the self-administration of alcohol**. *J Consult Clin Psychol* 1985, **53**(2):189-200.
68. Kivlahan DR, Marlatt GA, Fromme K, Coppel DB, Williams E: **Secondary prevention with college drinkers: Evaluation of an alcohol skills training program**. *J Consult Clin Psych* 1990, **58**(6):805-810.
69. Berman AH, Bergman H, Palmstierna T, Schlyter F: **Evaluation of the Drug Use Disorders Identification Test (DUDIT) in criminal justice and detoxification settings and in a Swedish population sample**. *European addiction research* 2005, **11**(1):22-31.
70. Hildebrand M: **The psychometric properties of the Drug Use Disorders Identification Test (DUDIT): A review of recent research**. *Journal of substance abuse treatment* 2015 a, **53**:52-59.
71. Sinadinovic K, Wennberg P, Berman AH: **Population screening of risky alcohol and drug use via Internet and Interactive Voice Response (IVR): A feasibility and psychometric study in a random sample**. *Drug and Alcohol Dependence* 2011, **114**(1):55-60.
72. Topp CW, Ostergaard SD, Sondergaard S, Bech P: **The WHO-5 Well-Being Index: a systematic review of the literature**. *Psychotherapy and psychosomatics* 2015, **84**(3):167-176.
73. Love J, Andersson L, Moore CD, Hensing G: **Psychometric analysis of the Swedish translation of the WHO well-being index**. *Quality of life research : an international journal of quality of life aspects of treatment, care and rehabilitation* 2014, **23**(1):293-297.
74. Strandberg AK, Skoglund C, Gripenberg J, Kvillemo P: **Alcohol and illicit drug consumption and the association with risky sexual behavior among Swedish youths visiting youth health clinics**. *Nordic Studies on Alcohol and Drugs* Accepted.
75. Brown BB, Clasen DR, Eicher SA: **Perceptions of peer pressure, peer conformity dispositions, and self-reported behavior among adolescents**. *Developmental Psychology* 1986, **22**(4):521-530.
76. Frost RO, Marten P, Lahart C, Rosenblate R: **The dimensions of perfectionism**. *Cognitive Therapy and Research* 1990, **14**(5):449-468.
77. Rozental A, Shafran R, Wade T, Egan S, Nordgren LB, Carlbring P, Landstrom A, Roos S, Skoglund M, Thelander E *et al*: **A randomized controlled trial of Internet-Based Cognitive Behavior Therapy for perfectionism including an investigation of outcome predictors**. *Behaviour research and therapy* 2017, **95**:79-86.

For peer review only

78. Stattin H, Kerr M: **Parental monitoring: a reinterpretation**. *Child development* 2000, **71**(4):1072-1085.
79. Salari R, Thorell LB: **Parental monitoring in late adolescence: Relations to ADHD symptoms and longitudinal predictors**. *Journal of Adolescence* 2015, **40**:24.
80. Kerr M, Stattin H: **What parents know, how they know it, and several forms of adolescent adjustment: further support for a reinterpretation of monitoring**. *Dev Psychol* 2000, **36**(3):366-380.
81. Miller WR, Rollnick S: **Motivational Interviewing: Helping People Change**, 3rd edn. New York: The Guilford Press; 2013.
82. Ajzen I: **The Theory of Planned Behavior**. *Organizational Behavior and Human Decision Processes* 1991, **50**(2):179-211.
83. Bandura A: **Social foundations of thought and action: A social cognitive theory**. NJ: Prentice-Hall: Englewood Cliffs; 1986.
84. Carey KB, Scott-Sheldon LAJ, Elliott JC, Bolles JR, Carey MP: **Computer-delivered interventions to reduce college student drinking: a meta-analysis**. *Addiction (Abingdon, England)* 2009, **104**(11):1807-1819.
85. Moreira MT, Smith LA, Foxcroft D: **Social norms interventions to reduce alcohol misuse in university or college students**. *The Cochrane database of systematic reviews* 2009(3):Cd006748.
86. Doumas DM, Hannah E: **Preventing high-risk drinking in youth in the workplace: a web-based normative feedback program**. *Journal of substance abuse treatment* 2008, **34**(3):263-271.
87. DONALDSON SI, SUSSMAN S, MacKINNON DP, SEVERSON HH, GLYNN T, MURRAY DM, STONE EJ: **Drug Abuse Prevention Programming:Do We Know What Content Works?** *American Behavioral Scientist* 1996, **39**(7):868-883.
88. Voogt CV, Poelen EAP, Kleinjan M, Lemmers LACJ, Engels RCME: **Targeting young drinkers online: the effectiveness of a web-based brief alcohol intervention in reducing heavy drinking among college students: study protocol of a two-arm parallel group randomized controlled trial**. *BMC Public Health* 2011, **11**(1):231.
89. Marlatt GA, Baer JS, Kivlahan DR, Dimeff LA, Larimer ME, Quigley LA, Somers JM, Williams E: **Screening and brief intervention for high-risk college student drinkers: Results from a 2-year follow-up assessment**. *J Consult Clin Psych* 1998, **66**(4):604-615.
90. Widmark E: **Die theoretischen Grundlagen und die praktische Verwendbarkeit der gerichtlich-medizinischen Alkoholbestimmung**. Berlin Wien: Urban und Schwarzenberg; 1932.
91. Babor T, Higgins-Biddle J: **Brief Intervention For Hazardous and Harmful Drinking: A Manual for use in Primary Care**: World health Organization; 2001.
92. Young RM, Hasking PA, Oei TP, Loveday W: **Validation of the Drinking Refusal Self-Efficacy Questionnaire--Revised in an Adolescent Sample (DRSEQ-RA)**. *Addictive behaviors* 2007, **32**(4):862-868.
93. Gibbons RD, Hedeker D, DuToit S: **Advances in analysis of longitudinal data**. *Annu Rev Clin Psychol* 2010, **6**:79-107.
94. Cole HA, Prassel HB, Carlson CR: **A Meta-Analysis of Computer-Delivered Drinking Interventions for College Students: A Comprehensive Review of Studies From 2010 to 2016**. *J Stud Alcohol Drugs* 2018, **79**(5):686-696.
95. Berman AH, Gajecki M, Sinadinovic K, Andersson C: **Mobile Interventions Targeting Risky Drinking Among University Students: A Review**. *Curr Addict Rep* 2016, **3**:166-174.

For peer review only

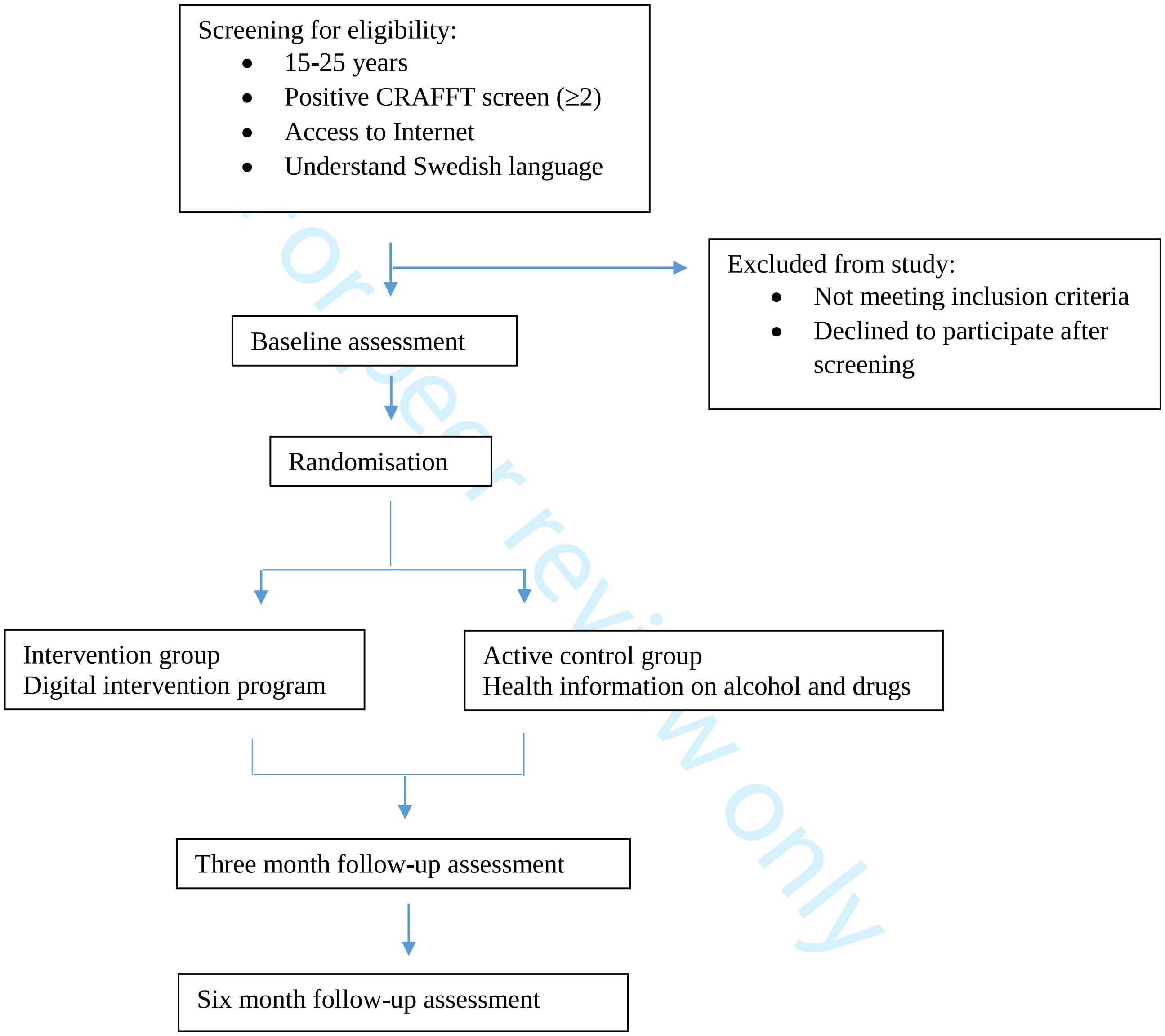

Figure 1 Study design

Reporting checklist for protocol of a clinical trial.

Based on the SPIRIT guidelines.

Instructions to authors

Complete this checklist by entering the page numbers from your manuscript where readers will find each of the items listed below.

Your article may not currently address all the items on the checklist. Please modify your text to include the missing information. If you are certain that an item does not apply, please write "n/a" and provide a short explanation.

Upload your completed checklist as an extra file when you submit to a journal.

In your methods section, say that you used the SPIRITreporting guidelines, and cite them as:

Chan A-W, Tetzlaff JM, Altman DG, Laupacis A, Gøtzsche PC, Krleža-Jerić K, Hróbjartsson A, Mann H, Dickersin K, Berlin J, Doré C, Parulekar W, Summerskill W, Groves T, Schulz K, Sox H, Rockhold FW, Rennie D, Moher D. SPIRIT 2013 Statement: Defining standard protocol items for clinical trials. Ann Intern Med. 2013;158(3):200-207

		Reporting Item	Page Number
**Administrative information**			
Title	#1	Descriptive title identifying the study design, population, interventions, and, if applicable, trial acronym	1, Title page

For peer review only

Trial registration	#2a	Trial identifier and registry name. If not yet registered, name of intended registry	1
Trial registration: data set	#2b	All items from the World Health Organization Trial Registration Data Set	1
Protocol version	#3	Date and version identifier	1
Funding	#4	Sources and types of financial, material, and other support	12
Roles and responsibilities: contributorship	#5a	Names, affiliations, and roles of protocol contributors	Title page
Roles and responsibilities: sponsor contact information	#5b	Name and contact information for the trial sponsor	"n/a" No sponsor, just funders
Roles and responsibilities: sponsor and funder	#5c	Role of study sponsor and funders, if any, in study design; collection, management, analysis, and interpretation of data; writing of the report; and the decision to submit the report for publication, including whether they will have ultimate authority over any of these activities	"n/a" No involvement

For peer review only

Roles and responsibilities: committees	#5d	Composition, roles, and responsibilities of the coordinating centre, steering committee, endpoint adjudication committee, data management team, and other individuals or groups overseeing the trial, if applicable (see Item 21a for data monitoring committee)	"n/a" No such org. or individuals involved
**Introduction**			
Background and rationale	#6a	Description of research question and justification for undertaking the trial, including summary of relevant studies (published and unpublished) examining benefits and harms for each intervention	2-5, 10-11
Background and rationale: choice of comparators	#6b	Explanation for choice of comparators	4-5
Objectives	#7	Specific objectives or hypotheses	4-5
Trial design	#8	Description of trial design including type of trial (eg, parallel group, crossover, factorial, single group), allocation ratio, and framework (eg, superiority, equivalence, non-inferiority, exploratory)	4-5
**Methods: Participants,**			

For peer review only

**interventions, and outcomes**			
Study setting	#9	Description of study settings (eg, community clinic, academic hospital) and list of countries where data will be collected. Reference to where list of study sites can be obtained	5
Eligibility criteria	#10	Inclusion and exclusion criteria for participants. If applicable, eligibility criteria for study centres and individuals who will perform the interventions (eg, surgeons, psychotherapists)	5
Interventions: description	#11a	Interventions for each group with sufficient detail to allow replication, including how and when they will be administered	7-9
Interventions: modifications	#11b	Criteria for discontinuing or modifying allocated interventions for a given trial participant (eg, drug dose change in response to harms, participant request, or improving / worsening disease)	"n/a" No such criteria
Interventions: adherance	#11c	Strategies to improve adherence to intervention protocols, and any procedures for monitoring adherence (eg, drug tablet return; laboratory tests)	5

For peer review only

Interventions: concomitant care	#11d	Relevant concomitant care and interventions that are permitted or prohibited during the trial	"n/a" No such care
Outcomes	#12	Primary, secondary, and other outcomes, including the specific measurement variable (eg, systolic blood pressure), analysis metric (eg, change from baseline, final value, time to event), method of aggregation (eg, median, proportion), and time point for each outcome. Explanation of the clinical relevance of chosen efficacy and harm outcomes is strongly recommended	6-7
Participant timeline	#13	Time schedule of enrolment, interventions (including any run-ins and washouts), assessments, and visits for participants. A schematic diagram is highly recommended (see Figure)	5
Sample size	#14	Estimated number of participants needed to achieve study objectives and how it was determined, including clinical and statistical assumptions supporting any sample size calculations	5
Recruitment	#15	Strategies for achieving adequate participant enrolment to reach target sample size	5

For peer review only

Methods: Assignment of interventions (for controlled trials)

Allocation: sequence generation	#16a	Method of generating the allocation sequence (eg, computer-generated random numbers), and list of any factors for stratification. To reduce predictability of a random sequence, details of any planned restriction (eg, blocking) should be provided in a separate document that is unavailable to those who enrol participants or assign interventions	7
Allocation concealment mechanism	#16b	Mechanism of implementing the allocation sequence (eg, central telephone; sequentially numbered, opaque, sealed envelopes), describing any steps to conceal the sequence until interventions are assigned	7
Allocation: implementation	#16c	Who will generate the allocation sequence, who will enrol participants, and who will assign participants to interventions	7
Blinding (masking)	#17a	Who will be blinded after assignment to interventions (eg, trial participants, care	7

For peer review only

		providers, outcome assessors, data analysts), and how	
Blinding (masking): emergency unblinding	#17b	If blinded, circumstances under which unblinding is permissible, and procedure for revealing a participant's allocated intervention during the trial	"n/a" Unblinding only when analyzing the data, or if participants themselves contact the research team and describe their intervention
**Methods: Data collection, management, and analysis**			
Data collection plan	#18a	Plans for assessment and collection of outcome, baseline, and other trial data, including any related processes to promote data quality (eg, duplicate measurements, training of assessors) and a description of study instruments (eg, questionnaires, laboratory tests) along with their reliability and validity, if known. Reference to where data collection forms can be found, if not in the protocol	6-7

For peer review only

Data collection plan: retention	#18b	Plans to promote participant retention and complete follow-up, including list of any outcome data to be collected for participants who discontinue or deviate from intervention protocols	5
Data management	#19	Plans for data entry, coding, security, and storage, including any related processes to promote data quality (eg, double data entry; range checks for data values). Reference to where details of data management procedures can be found, if not in the protocol	10-12
Statistics: outcomes	#20a	Statistical methods for analysing primary and secondary outcomes. Reference to where other details of the statistical analysis plan can be found, if not in the protocol	10
Statistics: additional analyses	#20b	Methods for any additional analyses (eg, subgroup and adjusted analyses)	10
Statistics: analysis population and missing data	#20c	Definition of analysis population relating to protocol non-adherence (eg, as randomised analysis), and any statistical methods to handle missing data (eg, multiple imputation)	10

Methods: Monitoring

For peer review only

Data monitoring: formal committee	#21a	Composition of data monitoring committee (DMC); summary of its role and reporting structure; statement of whether it is independent from the sponsor and competing interests; and reference to where further details about its charter can be found, if not in the protocol. Alternatively, an explanation of why a DMC is not needed	"n/a" Only the research team is involved in these procedures
Data monitoring: interim analysis	#21b	Description of any interim analyses and stopping guidelines, including who will have access to these interim results and make the final decision to terminate the trial	10-11
Harms	#22	Plans for collecting, assessing, reporting, and managing solicited and spontaneously reported adverse events and other unintended effects of trial interventions or trial conduct	10-11
Auditing	#23	Frequency and procedures for auditing trial conduct, if any, and whether the process will be independent from investigators and the sponsor	"n/a" No such procedures

Ethics and dissemination

For peer review only

Research ethics approval	#24	Plans for seeking research ethics committee / institutional review board (REC / IRB) approval	10
Protocol amendments	#25	Plans for communicating important protocol modifications (eg, changes to eligibility criteria, outcomes, analyses) to relevant parties (eg, investigators, REC / IRBs, trial participants, trial registries, journals, regulators)	10-11
Consent or assent	#26a	Who will obtain informed consent or assent from potential trial participants or authorised surrogates, and how (see Item 32)	5
Consent or assent: ancillary studies	#26b	Additional consent provisions for collection and use of participant data and biological specimens in ancillary studies, if applicable	"n/a" No additional consent
Confidentiality	#27	How personal information about potential and enrolled participants will be collected, shared, and maintained in order to protect confidentiality before, during, and after the trial	10-11
Declaration of interests	#28	Financial and other competing interests for principal investigators for the overall trial and each study site	12

For peer review only

Data access	#29	Statement of who will have access to the final trial dataset, and disclosure of contractual agreements that limit such access for investigators	12
Ancillary and post trial care	#30	Provisions, if any, for ancillary and post-trial care, and for compensation to those who suffer harm from trial participation	11
Dissemination policy: trial results	#31a	Plans for investigators and sponsor to communicate trial results to participants, healthcare professionals, the public, and other relevant groups (eg, via publication, reporting in results databases, or other data sharing arrangements), including any publication restrictions	10-11
Dissemination policy: authorship	#31b	Authorship eligibility guidelines and any intended use of professional writers	10-11
Dissemination policy: reproducible research	#31c	Plans, if any, for granting public access to the full protocol, participant-level dataset, and statistical code	"n/a" No such plans
**Appendices**			
Informed consent materials	#32	Model consent form and other related documentation given to participants and authorised surrogates	5

For peer review only

Biological specimens	#33	Plans for collection, laboratory evaluation, and storage of biological specimens for genetic or molecular analysis in the current trial and for future use in ancillary studies, if applicable	"n/a" No biological specimens

None The SPIRIT checklist is distributed under the terms of the Creative Commons Attribution License CC-BY-ND 3.0. This checklist can be completed online using https://www.goodreports.org/, a tool made by the EQUATOR Network in collaboration with Penelope.ai

For peer review only

BMJ Open

Effects of an automated digital brief prevention intervention targeting adolescents and young adults with risky substance use: Study protocol for a randomised controlled trial

Journal:	*BMJ Open*
Manuscript ID	bmjopen-2019-034894.R1
Article Type:	Protocol
Date Submitted by the Author:	18-Dec-2019
Complete List of Authors:	Kvillemo, Pia; Karolinska Institutet, Department of Clinical Neuroscience, STAD, Centre for Psychiatry Research, Stockholm Health Care Services, Stockholm Region<br>Strandberg, Anna; Karolinska Institutet, Department of Clinical Neuroscience, STAD, Centre for Psychiatry Research, Stockholm Health Care Services, Stockholm Region<br>Gripenberg, Johanna; Karolinska Institutet, Department of Clinical Neuroscience, STAD, Centre for Psychiatry Research, Stockholm Health Care Services, Stockholm Region<br>Berman, Anne; Karolinska Institutet, Department of Clinical Neuroscience, Centre for Psychiatry Research, Stockholm Health Care Services, Stockholm Region<br>Skoglund, Charlotte; Karolinska Institutet, Department of Clinical Neuroscience, STAD, Centre for Psychiatry Research, Stockholm Health Care Services, Stockholm Region<br>Elgán, Tobias; Karolinska Institutet, Department of Clinical Neuroscience, STAD, Centre for Psychiatry Research, Stockholm Health Care Services, Stockholm Region
**Primary Subject Heading**:	Public health
Secondary Subject Heading:	Addiction, Medical education and training, Mental health, Sexual health
Keywords:	PSYCHIATRY, Substance misuse < PSYCHIATRY, Child & adolescent psychiatry < PSYCHIATRY, PREVENTIVE MEDICINE

SCHOLARONE™
Manuscripts

I, the Submitting Author has the right to grant and does grant on behalf of all authors of the Work (as defined in the below author licence), an exclusive licence and/or a non-exclusive licence for contributions from authors who are: i) UK Crown employees; ii) where BMJ has agreed a CC-BY licence shall apply, and/or iii) in accordance with the terms applicable for US Federal Government officers or employees acting as part of their official duties; on a worldwide, perpetual, irrevocable, royalty-free basis to BMJ Publishing Group Ltd ("BMJ") its licensees and where the relevant Journal is co-owned by BMJ to the co-owners of the Journal, to publish the Work in this journal and any other BMJ products and to exploit all rights, as set out in our licence.

The Submitting Author accepts and understands that any supply made under these terms is made by BMJ to the Submitting Author unless you are acting as an employee on behalf of your employer or a postgraduate student of an affiliated institution which is paying any applicable article publishing charge ("APC") for Open Access articles. Where the Submitting Author wishes to make the Work available on an Open Access basis (and intends to pay the relevant APC), the terms of reuse of such Open Access shall be governed by a Creative Commons licence – details of these licences and which Creative Commons licence will apply to this Work are set out in our licence referred to above.

Other than as permitted in any relevant BMJ Author's Self Archiving Policies, I confirm this Work has not been accepted for publication elsewhere, is not being considered for publication elsewhere and does not duplicate material already published. I confirm all authors consent to publication of this Work and authorise the granting of this licence.

For peer review only

Effects of an automated digital brief prevention intervention targeting adolescents and young adults with risky substance use: Study protocol for a randomised controlled trial

Authors

Pia Kvillemo (corresponding author)
STAD, Centre for Psychiatry Research
Department of Clinical Neuroscience, Karolinska Institutet and Stockholm Health Care Services, Stockholm Region
Norra Stationsgatan 69, SE-113 64 Stockholm, Sweden
+46 (0)70 673 48 64
pia.kvillemo@ki.se

Anna K Strandberg
STAD, Centre for Psychiatry Research
Department of Clinical Neuroscience, Karolinska Institutet and Stockholm Health Care Services, Stockholm Region
Stockholm, Sweden

Johanna Gripenberg
STAD, Centre for Psychiatry Research
Department of Clinical Neuroscience, Karolinska Institutet and Stockholm Health Care Services, Stockholm Region
Stockholm, Sweden

Anne H Berman
Centre for Psychiatry Research
Department of Clinical Neuroscience, Karolinska Institutet and Stockholm Health Care Services, Stockholm Region
Stockholm, Sweden

Charlotte Skoglund
STAD, Centre for Psychiatry Research
Department of Clinical Neuroscience, Karolinska Institutet and Stockholm Health Care Services, Stockholm Region
Stockholm, Sweden

Tobias H Elgán
STAD, Centre for Psychiatry Research
Department of Clinical Neuroscience, Karolinska Institutet and Stockholm Health Care Services, Stockholm Region
Stockholm, Sweden

Keywords
Motivational Interviewing, Decisional balance, Substance use, Web-based intervention, Normative feedback.

Wordcount
4624

For peer review only - http://bmjopen.bmj.com/site/about/guidelines.xhtml

Effects of an automated digital brief prevention intervention targeting adolescents and young adults with risky substance use: Study protocol for a randomised controlled trial

ABSTRACT

Introduction

Adolescence and young adulthood is a period in life when individuals may be especially vulnerable to harmful substance use. Several critical developmental processes are occurring in the brain, and substance use poses both short- and long-term risks with regard to mental health and social development. From a public health perspective, it is important to prevent or delay substance use to reduce individual risk and societal costs. Given the scarcity of effective interventions targeting substance use among adolescents and young adults, cost-effective and easily disseminated interventions are warranted. The current study will test the effectiveness of a fully automated digital brief intervention aimed at reducing substance use in adolescents and young adults aged 15-25 years.

Methods and analysis

A two-arm double-blind randomised controlled trial (RCT) design is applied to assess the effectiveness of an online digital brief intervention. Baseline assessment and two follow-up assessments at three and six months will be carried out. The aim is to include 800 participants with risky substance use based on the screening tool CRAFFT. Recruitment, informed consent, randomisation, intervention, and follow-up will be implemented online. The primary outcome is reduction in alcohol use. Secondary outcomes concern binge drinking, frequency of alcohol consumption, amount of alcohol consumed a typical day when alcohol is consumed, other substance use, mental health, sexual risk behaviours, and perceived peer pressure. Moreover, the study involves analyses of potential moderators including perfectionism, openness to parents, help-seeking, and background variables.

For peer review only

For peer review only - http://bmjopen.bmj.com/site/about/guidelines.xhtml

Ethics and dissemination

The study was approved by the Swedish Ethical Review Authority (no. 2019-03249). Prior to inclusion, participants must give informed consent online. The trial is expected to expand the knowledge on digital preventive interventions for substance using adolescents and young adults. Results will be disseminated in research journals, at conferences and via the media.

Registration

24/09/2019, ID: ISRCTN91048246.

Key words

Motivational Interviewing, Decisional balance, Substance use, Web-based intervention, Normative feedback.

ARTICLE SUMMARY

Strengths and limitations of this study

- A double-blind randomised controlled trial (RCT) is considered to be the most robust experimental design, enabling causal inferences, controlling for selection bias and participant allocation bias.
- An active control condition, blinded to the study condition, controls for expectation, detection and performance bias.
- Analysis of several potential moderators, i.e., perfectionism, openness to parents, help-seeking and background variables will contribute to the understanding of possible effects.
- A convenience sample may reduce generalizability due to possible selection bias at recruitment.

For peer review only

INTRODUCTION

Substance use is a major public health concern causing individual suffering as well as societal costs.[1-3] Substance use in childhood and younger ages is particularly harmful since the brain is undergoing critical development during this time in life, which makes it more vulnerable to addictive substances.[4 5] In adolescence, some individuals are especially prone to various risk behaviours, increasing the risk of e.g., substance use.[6] Additionally, having substance using peers is a prominent risk factor for one's own substance use at this age, since peers are increasingly important in teenagers' social life.[7] Thus, the initiation of substance use often occurs during this period.[6] After initiation, the use of substances frequently increases during adolescence and young adulthood, posing a number of risks for the individual.[2 8 9] Early onset of substance use implies a risk of severe adverse effects on psychological and physiological development.[2 8 9] Hazardous use of alcohol, cannabis and other substances can lead to chronic problematic consumption patterns and addiction that can significantly influence the developmental trajectory during the transition from childhood to adulthood.[10] More acute consequences include, e.g., mental or psychiatric problems, problems of academic adjustment,[11] accidents,[12] and problems with the police or legal authorities.[1314] Risk behaviours, such as substance use, binge drinking, intoxication, and sexual risk behaviour often co-occur, and the use of lllicit substances, is e.g., often accompanied by alcohol consumption.[1 2 11 15-19] Poly-substance use, i.e., simultaneous use of different substances, may imply an increased risk of various negative outcomes, e.g., mental health problems, shown to increase in magnitude and over time, with the number of substances used.[20] The well-established association between substance use and mental health problems is bidirectional, sometimes manifested in self-medication for

For peer review only - http://bmjopen.bmj.com/site/about/guidelines.xhtml

mental health problems, and sometimes as an increase in such problems due to substance use.[21] While most of prior research shows that low socio-economic standard is a risk factor for substance use and related problems,[22 23] the current study is informed by recent research on mechanisms involved in adolescence alcohol and other substance use among upper secondary school students from affluent areas, showing associations between internalizing symptoms and substance use,[24 25] as well as associations between achievement pressures (particularly excessive perfectionistic strivings), and isolation from parents (particularly low perceived closeness to mothers).[24 26] The latter finding is in line with previous research showing that a positive parent–child relationship is a protective factor related to lower likelihood of adolescence substance use.[27 28]

Although alcohol use has declined among adolescents during recent years, some individuals drink more and binge drinking is still highly prevalent among young adults, where the largest proportion of alcohol risk consumers is still found.[29 30] Moreover, a high prevalence of substance use and poly-substance use among young people in Europe was recognized in the EU Drug Strategy 2005–2012, which calls for action. [31] Also, established cannabis users among Swedish upper secondary school students tend to use cannabis more often than before, according to recent survey data.[32] In light of these circumstances, it is of utmost importance to target and tailor attractive preventive interventions for adolescents and young adults. One way to reach large groups of young people is to disseminate digital interventions online (i.e., via the Internet). In Sweden, 98 percent of the population has access to the Internet at home and virtually 100 percent of 16-25 year-olds use the Internet in mobile phones, tablets or computers.[33] Digital interventions have several advantages over traditional ones. They may reduce the stigma around risk behaviours, they are accessible at any time and place, and can be cost-effective because minimal staff resources are used for implementation, especially if they are fully automated.[34 35] Digital interventions may thus be particularly suitable for adolescents and young adults.[36] To date, digital prevention programmes targeting alcohol and other substance use among adolescents and young adults that meet scientific evaluation criteria are scarce. An early example is eScreen.se, offering screening for alcohol and drug use with personalized feedback.[37] A more recent example is a fully automated brief motivational intervention for substance-using 16-18 year-olds, tested in an RCT in four European countries,[38-40] where our research group was one of the partners. The intervention, named WISEteens, relied on Motivational Interviewing (MI)[41] and models of Social Influence.[42 43] The goal of the MI approach was to enhance motivation to change by exploring and resolving ambivalence about substance-related behaviours. An important element in the intervention was personalized feedback about one's substance use behaviours in relation to normative comparisons, as personalized feedback is perceived as more relevant for changing behavior than more general information.[44] The normative feedback included information about how a specific

For peer review only - http://bmjopen.bmj.com/site/about/guidelines.xhtml

reference group actually consumed substances, in order to correct participants' "inflated perception",[45] which has proven effective for reduction of alcohol consumption in young adults in previous meta-analyses.[46 47] For adolescents and young adults, a normative feedback approach may be particularly appealing, assuming they are curious about how their substance consumption compares to their peers.[48] Likewise, an important element of the intervention was a focus on substance-related social norms and training on how to avoid social high-risk situations and how to resist peer pressure; i.e., raising refusal self-efficacy.[49] In line with the social influence hypothesis stating that a prominent risk factor for substance use in adolescence is the influence of substance using peers,[7] targeting peer pressure in brief digital interventions has been an important element for reducing alcohol use among adolescents.[50] Wiseteens demonstrated significant between-group effects for alcohol use, indicating that a targeted brief motivational intervention in a fully automated digital format can be effective to reduce drinking and lower barriers for accessing substance use service in hazardous drinking adolescents.[38]

Digital interventions, often delivered online, generally offer adaptations of evidence based face-to-face interventions, e.g., MI. The effects of MI on several health related behaviours have been evaluated in a number of other studies, especially among adults. [51] Among adolescents, systematic reviews and meta-analyses on MI for various health-related behaviours show mixed results. A review of six studies of MI for reducing alcohol consumption in the emergency room setting suggested that MI was at least as effective as other brief interventions in the same setting.[52] Another review of 24 studies of different brief interventions for reducing alcohol consumption and alcohol-related problems among adolescents showed a significant effect that persisted up to one year compared to control interventions, with greater effects for MI. Effects were consistent over diverse settings and particularly effective components included decisional balance (juxtaposition of pros and cons of change)[53] and goal-setting[54]. Regarding MI for illicit drug use among adolescents, a review of 10 studies showed no effects on drug use behaviors; however, changes in attitudes towards drug use were found, which could be translated into intentions to change behaviors [53]. A scoping review concerning MI for reducing sexual risk behaviors among adolescents identified 29 unique studies with varying designs and conceptualizations of MI and specific risk behaviours, making it difficult to generalize regarding outcomes but indicating the need of more research.[55] MI has most commonly been delivered as an individual face-to-face intervention[51], but has also been provided in other forms, e.g., via telephone or digitally, with various results on e.g., substance use behaviours.[56 57] Digital programmes based on MI have demonstrated effect in the form of reduced alcohol consumption among young adults.[58 59] Additionally, a combination of screening with a short intervention has shown similar effects on alcohol consumption among adolescents.[60] Brief motivational interventions are empirically supported individual level interventions for reducing alcohol consumption.[45 61] Such

For peer review only - http://bmjopen.bmj.com/site/about/guidelines.xhtml

interventions have in the last decades also been digitally provided.[62 63] Digital interventions can be provided with or without human guidance, i.e., more or less automated.[64] Previous research has demonstrated that even fully automated interventions can reduce alcohol-related problems for young people with risky alcohol consumption up to 12 months after implementation,[59 65] and indicated potential effect on cannabis use in certain groups.[66 67] Although various digital substance use prevention interventions have been developed and tested with promising results,[38 68] studies on the effects of fully automated digitally delivered and MI-based substance use prevention programmes for adolescents and young adults are still few[68] and more well-designed studies are warranted in order to obtain evidence for potential effects.

Aim

The study described in this protocol builds on results previously obtained in a European study of a digital brief MI-based substance use prevention intervention (WISEteens), delivered online to adolescents in a fully automated form.[38] In this study, the effectiveness of a modified version of this intervention will be tested across a range of outcome measures among 15-25 year-olds with hazardous alcohol or other substance use. The primary aim is reduction in frequency and quantity of substance use.

Rationale and hypothesis

The current study will add to existing evidence regarding the effectiveness of digital interventions aimed at reducing alcohol and other substance use among young people. We hypothesize that participants in the intervention group will report reduced substance use (primarily alcohol consumption at three-month follow-up), with a larger effect size compared to an active control group receiving health information about various substances. A number of potential moderators will be assessed to confirm or contradict previous research on factors influencing the development of substance use patterns over time and effects of prevention interventions in different target groups.

METHODS AND ANALYSIS

In order to evaluate the effectiveness of the digital screening and brief motivational intervention, a double-blind two-arm RCT study design is planned with baseline assessment at study entry and follow-up assessment at three and six-months across a number of outcome variables. Figure 1 displays the trial

For peer review only - http://bmjopen.bmj.com/site/about/guidelines.xhtml

design. We will also explore and test for moderator effects. To outline and report the current study we used SPIRIT reporting guidelines.[69]

Recruitment

The target group for the current study is adolescents and young adults aged 15-25 years with self-reported substance use, a capacity to understand the Swedish language, and access to the Internet. Based on a power calculation to detect a small effect size (Cohen's d=0.2),[70] the aim is to include 800 participants.[71] The participants will be recruited during January-October 2020 by offline marketing at youth health clinics, other health care units for youth, and at upper secondary schools in Stockholm county, as well as by social networks, using online banner advertisements. This will result in a convenience sample from the general Swedish population.[44] Results from follow-up measures are to be completely received in 2021. If sufficient number of participants are not included according to plan, we will extend the recruitment period and intensify the advertising. To enhance participation and follow-up rates, an incentive in the form of a movie ticket, with a value up to 15 Euros, for each completed follow-up assessment will be provided, i.e., up to 3 tickets. Up to three reminders will be sent out if participants do not respond to follow-up assessments.

Screening and informed consent

Potential participants will be guided to an online landing page with screening by an adapted version of the screening tool for use of alcohol and other substances, CRAFFT (Car, Relax, Alone, Forget, Friends, Trouble).[72] This six-item screening tool has demonstrated criterion validity and appropriateness for identifying substance-related problems among adolescents.[73-75] The primary eligibility criterion for participating in the study will be a score of 2 or more on the CRAFFT, since this value has shown satisfactory sensitivity for identifying substance use problems.[72] Those who score 2 or higher will be offered participation in the study and given information on confidentiality, voluntariness of participation, and human subject protections.[44] They will also be provided with contact information on suitable counselling service providers. Those who agree to participate will be asked to give digital informed consent.

For peer review only - http://bmjopen.bmj.com/site/about/guidelines.xhtml

Assessment

All assessment will occur online. The selection of measures and associated instruments is based on the aim of the study and the theoretical base concerning factors influencing substance use. Based on results from the previous European WISEteens study,[38] showing significant effects on alcohol consumption but not on other substance use, the primary outcome is alcohol consumption at three-month follow-up. Secondary outcomes include alcohol consumption at six-month follow-up, binge drinking, frequency of alcohol consumption, amount of alcohol consumed a typical day when alcohol is consumed at three- and six-months follow-ups, other substance use, mental health, sexual risk behaviours, and perceived peer pressure at three and six-month follow-ups. The moderating variables include perfectionism, openness to parents, help-seeking due to mental health problems, and socio-demographic and personal characteristics, (i.e. sex, age, residence, occupation, school performance (for students), and parents' education).

Measures

Alcohol use will be measured using two instruments. One of them is the short version of the Alcohol Use Disorders Identification Test (AUDIT-C),[76] measuring frequency and amount of consumed alcohol and frequency of binge drinking, providing a widely used and valid index sum score for problematic alcohol use among adolescents.[75 77] The other is the Daily Drinking Questionnaire (DDQ),[78] measuring the quantity and frequency of alcohol use a typical week. Previous research has demonstrated that the DDQ is highly correlated with other measures of self-reported alcohol consumption.[79] Other substance use will be measured by the first four items of the Drug Use Disorder Identification Test (DUDIT),[80] assessing frequency of consumption of drugs other than alcohol, frequency of different types of drugs other than alcohol used at the same occasion (1 = never to 5 = four or more times a week), and number of occasions when drugs other than alcohol are consumed on a typical day of drug use (1 = zero to 5 = seven or more). The DUDIT has been found to be effective in screening for drug-related problems in clinically selected groups,[81] and has proven useful in the context of public health surveys in Sweden.[80 82] Mental health will be measured by The WHO-5 Well-Being Index, which has demonstrated validity both as a screening tool for depression and as an outcome measure in clinical trials.[83] This scale has been successfully applied across a wide range of study fields, translated into more than 30 languages, including Swedish,[84] and found psychometrically sound. Changes in sexual risk behaviours will be measured by multiple choice questions on sexual behaviour under the influence of alcohol and/or other substances and unprotected sex, previously used in a survey among Swedish visitors at youth health clinics in Stockholm county.[85] Changes in perceived peer pressure will be measured by two items retrieved and adapted from the Peer pressure inventory.[86] Perfectionism will be measured by two subscales of the Frost Multidimensional

For peer review only - http://bmjopen.bmj.com/site/about/guidelines.xhtml

Perfectionism Scale (FMPS).[87 88] Openness to parents will be measured among participants who are up to 20 years old with questions about disclosure from Stattin & Kerr,[89-91] slightly modified into statements to be compatible with the digital online format. Help-seeking due to mental health problems will be measured by dichotomous questions regarding if, and to what healthcare provider, the participant has turned for help. Additionally, background data will be asked for using multiple choice questions: sex (man, women, other), age (15, 16…25), residence (name of municipality), occupation (secondary school, upper secondary school, university, not student, working, practicing, unemployed, other), school performance (merit value for students), and parents' education (secondary school, upper secondary school, university, don't know).

Randomisation

The study is double-blind, thus neither the participant nor the researchers will know which participant is allocated to the intervention or to the active control condition. After baseline assessment, the participants will be automatically randomised to one of the two study groups by a computer programme using an unrestricted randomisation protocol. Participants will then be informed about the name of their programme and given access immediately.

The intervention

Participants allocated to the intervention group will initially be asked to give additional information on alcohol and other substance use and to state their body weight, in order to generate personalized feedback on substance use behaviour and to estimate alcohol blood concentration on a typical drinking occasion.

Description of the intervention content

The intervention is interactive, digitally delivered online in a fully automated form, and requires approximately 20 minutes to complete. Personalized feedback is given to the participants based on their responses to previous assessment and suggestions on how to respond to this feedback are provided. This interactivity imitates a face-to face "dialogue" with techniques from MI such as an empathic approach, rolling with resistance, aiming at creating a dissonance between actual and desired behaviour, raising self-efficacy, and at the same time avoiding argumentation.[57] The intervention consists of three main components outlined below, and additional health related information.

For peer review only - http://bmjopen.bmj.com/site/about/guidelines.xhtml

Personalized feedback

The personalized feedback includes an estimation of the participants' blood-alcohol-concentration (BAC) and information on the associated risks concerning the participants' heaviest drinking episode during the past 30 days. The value will be based on a measure of Peak Drinking Quantity[92] and estimated using the Widmark-formula, which takes into account weight and sex.[93] The participants will receive graphed feedback regarding number of standard alcohol units per week that they think their peers consume (descriptive norms), as well as the participant's individual levels of consumption in relation to comparative data (actual drinking levels) from a reference group. Comparative data (AUDIT-C scores) will be taken from alcohol prevalence estimates found in a nationally representative sample of 16-25 year-olds undertaken by the Public Health Agency of Sweden. Comparative feedback will be available for drinking but not for substances other than alcohol.

Interactive MI-based exercises

The exercises provided in the current intervention build on the assumption that participants may hold certain levels of ambivalence about their current substance use, and that if they are willing to make a change they may not know how to, or may not be confident that they are able to.[41] Therefore, the intervention uses importance and confidence rulers with a short summary and feedback to encourage participants to reflect on personal reasons for change and explore personal strengths and ability to change. Furthermore, the programme provides a decisional balance to pick up and graphically illustrate potential levels of ambivalence by offering the participants a list of possible pros and cons regarding the decision to change their current substance use.[57 94] Participants are instructed to choose statements that apply to them and are presented with the resulting balance sheet of their personal comparative potential gains and losses.

Practical advice

The intervention focuses on raising self-efficacy for being able to avoid drinking in social situations, if desirable.[50] The participant will be asked to select three among 12 provided drinking situations that they consider most tempting and rank them. The situations are adapted from the adolescent version of the Drinking Refusal Self-efficacy Questionnaire (DRSEQ-RA).[95] According to the selection, a number of strategies will be offered for each of the selected drinking situations to provide participants with a tool kit necessary for engaging in and maintaining their behavioural goal.

Health related information connected to substance use

Finally, the intervention programme includes health related information associated with substance use. The information is provided optionally throughout the program behind "read more" buttons and also at the end of the programme. The information contains statements regarding risks connected to substance use

For peer review only - http://bmjopen.bmj.com/site/about/guidelines.xhtml

that the participant can reflect on, as well as optional links for more information. There are also two FAQ-sections about alcohol and cannabis, respectively, as well as information about how different substances may affect the individual, both physically and mentally. Finally, the information also contains cases, were the participant can read about some typical young persons who have used substances, why they used and what negative effects they have noted, and why they chose to stop using.

The control condition

The control group will receive the same general health related information as the intervention group, i.e., the additional information which is connected to the intervention programme.

Statistical analysis

Randomisation-checks of baseline variables regarding alcohol and other substances use and psychological state will be conducted using multivariate analyses of variance MANOVA. To test the effectiveness of the intervention, we will assess whether participants in the intervention group to a larger extent report decreased substance use, sexual risk behaviours, perceived peer-pressure, and improved mental health after three and six months, respectively, compared to participants in the active control group. Data analyses will consist of comparing outcome measurements with regards to within-group and between-group differences according to the intention-to-treat (ITT) principle in the primary analysis, accounting for all included participants regardless of whether or not they completed follow-up assessments. We will also perform per protocol analyses. The reason for choosing ITT in the primary analysis is the ambition to maintain the power of the study and also to avoid biased results due to selection of those completing all follow-ups, as they may have special characteristics not representative for the whole study group, which might influence study results. The main analysis of effectiveness will use mixed effects regression models, which can be applied to both continuous and categorical outcomes and also non-normally distributed outcomes. Moreover, mixed-effects regression models are robust to missing data in longitudinal studies.[96] In the current study we assume a quite large dropout rate with data missing at random (MAR), which can be handled using mixed effects regression models. Separate models will be run to test each outcome, i.e., alcohol consumption, binge drinking, frequency of alcohol consumption, amount of alcohol consumed a typical day when alcohol is consumed at three- and six-months follow-ups, other substance use, mental health, sexual risk behaviours and perceived peer pressure. Effect sizes will be calculated separately for the two measures used for the primary outcome variable (AUDIT-C and DDQ). Potential moderators, including perfectionism, openness to parents, help-seeking due to mental health problems, and socio-demographic and personal characteristics, (i.e. sex, age, residence, occupation, school performance (for

For peer review only - http://bmjopen.bmj.com/site/about/guidelines.xhtml

students), and parents' education) will serve as covariates in analyses of intervention effects. If moderator effects are found, we will carry out post-hoc analyses stratified by the detected variable.

Patient and public involvement statement

Patients and the public were not involved in the design and planning of the study, except for persons at the age of the target group reading manuscripts for the revised version of Wiseteens and also participating in a pilot-test.

For peer review only

ETHICS AND DISSEMINATION

The current study was approved by the Swedish Ethical Review Authority (no. 2019-03249) and registered 24/09/2019, pre-result, on IRCTN, ID: ISRCTN91048246. Any important protocol modifications will be reported to IRCTN. For inclusion, all participants must give informed consent online prior to participation in the study. In order to participate, the participants will need to state a username (which may be fictitious or a pseudonym) and an email address. All data collection will be done without collecting personal identification information, only personal e-mail addresses. The e-mail address will be used to connect data from the baseline measurement to the follow-up measurements. At a later stage, the raw data file will be anonymized and each person assigned a number instead of the e-mail address. The data will be stored in line with routines for handling and storing research data at Karolinska Institutet. All data is handled confidentially and will not be forwarded to third parties. Participating in the present study means that the participants need to reflect on their alcohol consumption and other substance use. In addition, questions will be asked concerning personal circumstances, including the participants' mental health and family relationships. These issues may be perceived as somewhat unpleasant. However, in the information that potential participants receive, they are informed that participation is voluntary and that they at any time can end their participation without explaining why. In addition, there will be reference to other types of official support (web pages and telephone numbers), if the advertisement or participation leads to concern about own substance use or related problems. Any issues brought up by the participants during the study will be documented and handled properly. Moreover, the research team includes professionals, such as a psychiatrist and a nurse, with possibility to refer participants to health care clinics if needed. The project's basic hypothesis is that the intervention will have positive effects, with regard to alcohol and other substance use among young people with risk use, and potential benefit may include decreased or ceased risk behaviours. Overall, the benefit for the research persons is considered to exceed

any risk of discomfort. Results will be disseminated in scientific peer reviewed journals, at conferences and via the media.

DISCUSSION OF STRENGTHS AND LIMITATIONS

The current study has a number of strengths. The protocol describes a two-arm double-blind RCT, considered to be the most robust experimental design, controlling for selection bias and participant allocation bias and with the possibility to make causal inferences. Moreover, an active control condition, blinded to the study condition, controls for expectation, detection and performance bias. The collection and analysis of information on potential moderators, allowing for control of these factors, facilitates the understanding of the possible effects. Thus, the present study will contribute to the literature on digital substance use prevention interventions among adolescents and young adults in several ways. The choice to study a digital intervention can from a public health perspective be regarded as positive. Digital brief interventions have several advantages over face-to-face approaches, e.g., the reduction of stigma around help-seeking for substance use and easy dissemination to large groups of people, which makes them cost-effective. Moreover, digital interventions have previously proven effective in addressing alcohol and other substance use in the general population and some studies have shown promising results also among adolescents and young adults.[97 98] The current intervention is well grounded in theory and incorporates elements of MI and social influence theory that has been shown to be effective in reducing problematic substance use in prior research.[57] Importantly, there are also some limitations to the current study. One limitation concerns selection bias and thereby external validity, as recruitment requires either that individuals click on our ads at social media to be considered for inclusion, or that they attend upper secondary schools, or have been in contact with existing clinics, aware of the opportunity to participate in the study. Thus, our participants may have certain personality traits, or are especially prone to help-seeking compared to a broader audience. The exclusion of people not understanding Swedish is also a limitation with regard to generalizability.

STATEMENTS

Author contributions

PK, AKS, JG, and THE obtained funding for the study. PK, THE, and CS further developed/modified the intervention. THE designed the study with contribution from PK, AKS, JG, AHB, and, CS. PK wrote this paper. All authors commented on successive manuscript drafts and approved the final version of the manuscript.

For peer review only - http://bmjopen.bmj.com/site/about/guidelines.xhtml

Funding statement

The work was supported by the National Public Health Agency of Sweden (grant no. 02299-2016-6.2) and the Alcohol Research Council of the Swedish Alcohol Retailing Monopoly (grant no. 2018-0016). Author AHB was supported by Swedish Research Council (grant no. K2012-61P-22131-01-6). The funding bodies had no role in study design, data collection, analysis, data interpretation or writing manuscripts.

Data statement

Collected data will be available from the Centre for Psychiatry Research, a collaboration between the Karolinska Institute and Region Stockholm, but restrictions apply to their availability, as they were used under ethical permission for the current study, and so are not publicly available. However, data are available from the authors upon reasonable request and with permission from the Centre for Psychiatry Research.

Competing interests

The authors declare no competing interests.

Acknowledgments

The authors would like to thank Dr. Silke Diestelkamp and Dr. Christiane Baldus for invaluable help in the process of building the Swedish version of the WISEteens programme.

For peer review only

For peer review only

REFERENCES

1. Dawson DA, Goldstein RB, Chou SP, et al. Age at first drink and the first incidence of adult-onset DSM-IV alcohol use disorders. *Alcoholism Clinical and Experimental Research* 2008;32(12):2149-60.
2. Hibell B, Guttormsson U, Ahlström S, et al. The 2007 ESPAD Report: Substance use among students in 35 European countries. ESPAD, The European School Survey Project on Alcohol and Other Drugs. Stockholm, Sweden, 2009.
3. Anderson P. Global alcohol policy and the alcohol industry. *Curr Opin Psychiatry* 2009;22(3):253-7. doi: 10.1097/YCO.0b013e328329ed75

For peer review only - http://bmjopen.bmj.com/site/about/guidelines.xhtml

4. Chambers RA, Taylor JR, Potenza MN. Developmental neurocircuitry of motivation in adolescence: a critical period of addiction vulnerability. *The American journal of psychiatry* 2003;160(6):1041-52. doi: 10.1176/appi.ajp.160.6.1041 [published Online First: 2003/06/05]
5. Barron S, White A, Swartzwelder HS, et al. Adolescent vulnerabilities to chronic alcohol or nicotine exposure: findings from rodent models. *Alcohol Clin Exp Res* 2005;29(9):1720-5. [published Online First: 2005/10/06]
6. Balogh KN, Mayes LC, Potenza MN. Risk-taking and decision-making in youth: relationships to addiction vulnerability. *Journal of behavioral addictions* 2013;2(1) doi: 10.1556/jba.2.2013.1.1 [published Online First: 2013/12/03]
7. Oetting ER, Beauvais F. Peer cluster theory: Drugs and the adolescent. *Journal of Counseling & Development* 1986;65(1):17-22. doi: 10.1002/j.1556-6676.1986.tb01219.x
8. Arria AM, Caldeira KM, Bugbee BA, et al. Marijuana use trajectories during college predict health outcomes nine years post-matriculation. *Drug and alcohol dependence* 2016;159:158-65. doi: 10.1016/j.drugalcdep.2015.12.009 [published Online First: 2015/12/23]
9. Van Der Vorst H, Vermulst AA, Meeus WH, et al. Identification and prediction of drinking trajectories in early and mid-adolescence. *Journal of clinical child and adolescent psychology : the official journal for the Society of Clinical Child and Adolescent Psychology, American Psychological Association, Division 53* 2009;38(3):329-41. doi: 10.1080/15374410902851648 [published Online First: 2009/05/14]
10. R M, E K, P M, et al. Impact of alcohol consumption on young people: A systematic review of published reviews. Newcastle University, 2009.
11. Hicks RD, Bemis Batzer G, Bemis Batzer W, et al. Psychiatric, Developmental, and Adolescent Medicine Issues in Adolescent Substance Use and Abuse. *Adolesc Med* 1993;4(2):453-68.
12. White V, Azar D, Faulkner A, et al. Adolescents' alcohol use and strength of policy relating to youth access, trading hours and driving under the influence: findings from Australia. *Addiction* 2018;113(6):1030-42. doi: 10.1111/add.14164
13. Arendt M, Rosenberg R, Foldager L, et al. Cannabis-induced psychosis and subsequent schizophrenia-spectrum disorders: follow-up study of 535 incident cases. *The British journal of psychiatry : the journal of mental science* 2005;187:510-5. doi: 10.1192/bjp.187.6.510 [published Online First: 2005/12/02]
14. Flory K, Lynam D, Milich R, et al. Early adolescent through young adult alcohol and marijuana use trajectories: early predictors, young adult outcomes, and predictive utility. *Development and psychopathology* 2004;16(1):193-213. [published Online First: 2004/04/30]
15. Nilses C, Blom H, Heimer G, et al. 40 procent av gymnasieelever riskbrukar alkohol. *Läkartidningen* 2011;34(108):1156-559.
16. Bonomo Y, Coffey C, Wolfe R, et al. Adverse outcomes of alcohol use in adolescents. *Addiction* 2001;96(10):1485-96.
17. Guttmannova K, Hill KG, Bailey JA, et al. Examining explanatory mechanisms of the effects of early alcohol use on young adult alcohol dependence. *J Stud Alcohol Drugs* 2012;73(3):379-90.
18. Miller JW, Naimi TS, Brewer RD, et al. Binge drinking and associated health risk behaviors among high school students. *Pediatrics* 2007;119(1):76-85.
19. Chick CF, Reyna VF. A fuzzy trace theory of adolescent risk taking: Beyond self-control and sensation seeking. In: V. Reyna SC, M. Dougherty, & J. Confrey (Eds.), ed. The Adolescent Brain Washington, DC: American Psychological Association. 2012.
20. Burdzovic Andreas J, Lauritzen G, Nordfjærn T. Co-occurrence between mental distress and poly-drug use: A ten year prospective study of patients from substance abuse treatment. *Addict Behav* 2015;48:71-78. doi: https://doi.org/10.1016/j.addbeh.2015.05.001

For peer review only

21. Hodgins S, Tengström A, Bylin S, et al. Consulting for substance abuse: Mental disorders among adolescents and their parents. *Nordic Journal of Psychiatry* 2007;61(5):379-86. doi: 10.1080/08039480701643423
22. Swift W, Coffey C, Carlin JB, et al. Adolescent cannabis users at 24 years: trajectories to regular weekly use and dependence in young adulthood. *Addiction* 2008;103(8):1361-70. doi: 10.1111/j.1360-0443.2008.02246.x [published Online First: 2008/10/16]
23. Von Sydow K, Lieb R, Pfister H, et al. What predicts incident use of cannabis and progression to abuse and dependence? A 4-year prospective examination of risk factors in a community sample of adolescents and young adults. *Drug and Alcohol Dependence* 2002;68:49–64.
24. Låftman SB, Almquist YB, Östberg V. Students' accounts of school-performance stress: a qualitative analysis of a high-achieving setting in Stockholm, Sweden. *Journal of Youth Studies* 2013;16(7):932-49. doi: 10.1080/13676261.2013.780126
25. Hiltunen L. Lagom perfekt. Erfarenheter av ohälsa bland unga tjejer och killar. Linnéuniversitetet, 2017.
26. Luthar SS, Becker BE. Privileged but pressured? A study of affluent youth. *Child development* 2002;73(5):1593-610. [published Online First: 2002/10/04]
27. Ashby Wills T, Yaeger AM. Family Factors and Adolescent Substance Use: Models and Mechanisms. *Current Directions in Psychological Science* 2003;12(6):222-26. doi: 10.1046/j.0963-7214.2003.01266.x
28. Wills TA, Cleary SD. How are social support effects mediated? A test with parental support and adolescent substance use. *Journal of Personality and Social Psychology* 1996;71(5):937-52. doi: 10.1037/0022-3514.71.5.937
29. Guttormsson U, Gröndal M. Befolkningens självrapporterade alkoholvanor 2004-2017 Stockholm: CAN, 2018.
30. Lees B, Mewton L, Stapinski L, et al. Binge drinking in young people: protocol for a systematic review of neuropsychological, neurophysiological and neuroimaging studies. *BMJ Open* 2018;8(7):e023629. doi: 10.1136/bmjopen-2018-023629
31. European Union Drugs Strategy 2013–2020, 2013.
32. Zetterqvist ME. Skolelevers drogvanor 2018. Stockholm: Centralförbundet för alkohol- och narkotikaupplysning (CAN) 2018.
33. Foundation TSI. Svenskarna och internet 2018 (The Swedes and the Internet 2018) (In Swedish) 2018 [Available from: https://2018.svenskarnaochinternet.se/allmant-om-internetutvecklingen/ accessed 2019-06-25.
34. Murray E. Web-Based Interventions for Behavior Change and Self-Management: Potential, Pitfalls, and Progress. *Med 20* 2012;1(2):e3. doi: 10.2196/med20.1741
35. Riper H, Blankers M, Hadiwijaya H, et al. Effectiveness of Guided and Unguided Low-Intensity Internet Interventions for Adult Alcohol Misuse: A Meta-Analysis. *PLOS ONE* 2014;9(6):e99912. doi: 10.1371/journal.pone.0099912
36. Spijkerman R, Roek AEM, Vermulst A, et al. Effectiveness of a Web-Based Brief Alcohol Intervention and Added Value of Normative Feedback in Reducing Underage Drinking: A Randomized Controlled Trial. *J Med Internet Res* 2010;12(5):e65. doi: 10.2196/jmir.1465
37. Sinadinovic K, Berman AH, Hasson D, et al. Internet-based assessment and self-monitoring of problematic alcohol and drug use. *Addictive behaviors* 2010;35(5):464-70. doi: 10.1016/j.addbeh.2009.12.021 [published Online First: 2010/01/23]
38. Arnaud N, Baldus C, Elgan TH, et al. Effectiveness of a Web-Based Screening and Fully Automated Brief Motivational Intervention for Adolescent Substance Use: A Randomized Controlled Trial. *J Med Internet Res* 2016;18(5):e103. doi: 10.2196/jmir.4643

For peer review only

39. Arnaud N, Bröning S, Drechsel M, et al. Web-based screening and brief intervention for poly-drug use among teenagers: study protocol of a multicentre two-arm randomized controlled trial. *BMC Public Health* 2012;12:826. doi: 10.1186/1471-2458-12-826 [published Online First: 2012/09/28]
40. Arnaud N, Baldus C, Elgán TH, et al. Moderators of Outcome in a Web-Based Substance Use Intervention for Adolescents. *SUCHT* 2015;61(6):377-87. doi: 10.1024/0939-5911.a000397
41. Miller WR, Rollnick S. Motivational Interviewing: Helping People Change. 3rd ed. New York: The Guilford Press 2013.
42. Ajzen I. The Theory of Planned Behavior. *Organizational Behavior and Human Decision Processes* 1991;50(2):179-211.
43. Bandura A. Social foundations of thought and action: A social cognitive theory. NJ: Prentice-Hall: Englewood Cliffs 1986.
44. Voogt CV, Poelen EA, Kleinjan M, et al. The effectiveness of the 'what do you drink' web-based brief alcohol intervention in reducing heavy drinking among students: a two-arm parallel group randomized controlled trial. *Alcohol Alcohol* 2013;48(3):312-21. doi: 10.1093/alcalc/ags133 [published Online First: 2013/01/11]
45. Wood MD, Fairlie AM, Fernandez AC, et al. Brief Motivational and Parent Interventions for College Students: A Randomized Factorial Study. *J Consult Clin Psych* 2010;78(3):349-61. doi: Doi 10.1037/A0019166
46. Carey KB, Scott-Sheldon LAJ, Elliott JC, et al. Computer-delivered interventions to reduce college student drinking: a meta-analysis. *Addiction (Abingdon, England)* 2009;104(11):1807-19. doi: 10.1111/j.1360-0443.2009.02691.x [published Online First: 2009/09/10]
47. Moreira MT, Smith LA, Foxcroft D. Social norms interventions to reduce alcohol misuse in university or college students. *The Cochrane database of systematic reviews* 2009(3):Cd006748. doi: 10.1002/14651858.CD006748.pub2 [published Online First: 2009/07/10]
48. Doumas DM, Hannah E. Preventing high-risk drinking in youth in the workplace: a web-based normative feedback program. *Journal of substance abuse treatment* 2008;34(3):263-71. doi: 10.1016/j.jsat.2007.04.006 [published Online First: 2007/06/30]
49. DONALDSON SI, SUSSMAN S, MacKINNON DP, et al. Drug Abuse Prevention Programming:Do We Know What Content Works? *American Behavioral Scientist* 1996;39(7):868-83. doi: 10.1177/0002764296039007008
50. Voogt CV, Poelen EAP, Kleinjan M, et al. Targeting young drinkers online: the effectiveness of a web-based brief alcohol intervention in reducing heavy drinking among college students: study protocol of a two-arm parallel group randomized controlled trial. *BMC Public Health* 2011;11(1):231. doi: 10.1186/1471-2458-11-231
51. Frost H, Campbell P, Maxwell M, et al. Effectiveness of Motivational Interviewing on adult behaviour change in health and social care settings: A systematic review of reviews. *PloS one* 2018;13(10):e0204890-e90. doi: 10.1371/journal.pone.0204890
52. Kohler S, Hofmann A. Can motivational interviewing in emergency care reduce alcohol consumption in young people? A systematic review and meta-analysis. *Alcohol Alcohol* 2015;50(2):107-17. doi: 10.1093/alcalc/agu098 [published Online First: 2015/01/08]
53. Li L, Zhu S, Tse N, et al. Effectiveness of motivational interviewing to reduce illicit drug use in adolescents: a systematic review and meta-analysis. *Addiction* 2016;111(5):795-805. doi: 10.1111/add.13285 [published Online First: 2015/12/22]
54. Tanner-Smith EE, Lipsey MW. Brief alcohol interventions for adolescents and young adults: a systematic review and meta-analysis. *Journal of substance abuse treatment* 2015;51:1-18. doi: 10.1016/j.jsat.2014.09.001 [published Online First: 2014/10/11]

For peer review only

For peer review only - http://bmjopen.bmj.com/site/about/guidelines.xhtml

55. Bahner J, Stenqvist K. Motivational Interviewing as Evidence-Based Practice? An Example from Sexual Risk Reduction Interventions Targeting Adolescents and Young Adults. *Sexuality Research and Social Policy* 2019 doi: 10.1007/s13178-019-00388-y
56. Gates P, Norberg M, Copeland J, et al. Randomized controlled trial of a novel cannabis use intervention delivered by telephone. *Addiction (Abingdon, England)* 2012;107 doi: 10.1111/j.1360-0443.2012.03953.x
57. Berman AH, Beckman M, Lindqvist H. Motivational Interviewing interventions. In: Hagger MS, Cameron LD, Hamilton K, et al., eds. Handbook of Behavior Change. New York, NY: Cambridge University Press. In press, 2020.
58. Bewick BM, Trusler K, Barkham M, et al. The effectiveness of web-based interventions designed to decrease alcohol consumption — A systematic review. *Preventive Medicine* 2008;47(1):17-26. doi: https://doi.org/10.1016/j.ypmed.2008.01.005
59. White A, Kavanagh D, Stallman H, et al. Online Alcohol Interventions: A Systematic Review. *J Med Internet Res* 2010;12(5):e62. doi: 10.2196/jmir.1479
60. Toumbourou JW, Stockwell T, Neighbors C, et al. Interventions to reduce harm associated with adolescent substance use. *Lancet (London, England)* 2007;369(9570):1391-401. doi: 10.1016/S0140-6736(07)60369-9
61. Natarajan M, Kaner E. Brief alcohol interventions in young people: evidence and applicability. I E. Gilvarry & P. McArdle (redaktörer), Alcohol, drugs and young people: clinical approaches. Newcastle upon Tyne: Mac Keith Press 2007.
62. Sinadinovic K, Wennberg P, Berman AH. Internet-based screening and brief intervention for illicit drug users: a randomized controlled trial with 12-month follow-up. *J Stud Alcohol Drugs* 2014;75(2):313-8. [published Online First: 2014/03/22]
63. Tait RJ, Spijkerman R, Riper H. Internet and computer based interventions for cannabis use: A meta-analysis. *Drug and Alcohol Dependence* 2013;133(2):295-304. doi: https://doi.org/10.1016/j.drugalcdep.2013.05.012
64. Cavanagh K, Millings A. (Inter)personal Computing: The Role of the Therapeutic Relationship in E-mental Health. *Journal of Contemporary Psychotherapy* 2013;43(4):197-206. doi: 10.1007/s10879-013-9242-z
65. Webb TL, Joseph J, Yardley L, et al. Using the internet to promote health behavior change: a systematic review and meta-analysis of the impact of theoretical basis, use of behavior change techniques, and mode of delivery on efficacy. *J Med Internet Res* 2010;12(1):e4. doi: 10.2196/jmir.1376 [published Online First: 2010/02/19]
66. Lee CM, Neighbors C, Kilmer JR, et al. A brief, web-based personalized feedback selective intervention for college student marijuana use: a randomized clinical trial. *Psychol Addict Behav* 2010;24(2):265-73. doi: 10.1037/a0018859 [published Online First: 2010/06/23]
67. Rooke S, Copeland J, Norberg M, et al. Effectiveness of a self-guided web-based cannabis treatment program: randomized controlled trial. *Journal of medical Internet research* 2013;15(2):e26-e26. doi: 10.2196/jmir.2256
68. Tonnesen H, H S, Pedersen B. Review: Web-based brief interventions for young adolescent alcohol and drug abusers - a systematic review2013.
69. Chan A-W, Tetzlaff JM, Altman DG, et al. SPIRIT 2013 statement: defining standard protocol items for clinical trials. *Ann Intern Med* 2013;158(3):200-07. doi: 10.7326/0003-4819-158-3-201302050-00583
70. Cohen J. A power primer. *Psychological bulletin* 1992;112(1):155-9. [published Online First: 1992/07/01]
71. Riper H, Hoogendoorn A, Cuijpers P, et al. Effectiveness and treatment moderators of internet interventions for adult problem drinking: An individual patient data meta-analysis of 19

For peer review only

randomised controlled trials. *PLOS Medicine* 2018;15(12):e1002714. doi: 10.1371/journal.pmed.1002714
72. Knight JR, Shrier LA, Bravender TD, et al. A new brief screen for adolescent substance abuse. *Arch Pediatr Adolesc Med* 1999;153(6):591-6. [published Online First: 1999/06/05]
73. Levy S, Sherritt L, Harris SK, et al. Test-retest reliability of adolescents' self-report of substance use. *Alcohol Clin Exp Res* 2004;28(8):1236-41. [published Online First: 2004/08/20]
74. Knight JR, Sherritt L, Harris SK, et al. Validity of brief alcohol screening tests among adolescents: a comparison of the AUDIT, POSIT, CAGE, and CRAFFT. *Alcohol Clin Exp Res* 2003;27(1):67-73. doi: 10.1097/01.ALC.0000046598.59317.3A [published Online First: 2003/01/25]
75. Kallmen H, Berman AH, Jayaram-Lindstrom N, et al. Psychometric Properties of the AUDIT, AUDIT-C, CRAFFT and ASSIST-Y among Swedish Adolescents. *European addiction research* 2019;25(2):68-77. doi: 10.1159/000496741 [published Online First: 2019/02/07]
76. Saunders JB, Aasland OG, Babor TF, et al. Development of the Alcohol Use Disorders Identification Test (AUDIT): WHO Collaborative Project on Early Detection of Persons with Harmful Alcohol Consumption--II. *Addiction* 1993;88(6):791-804.
77. Rumpf HJ, Wohlert T, Freyer-Adam J, et al. Screening questionnaires for problem drinking in adolescents: performance of AUDIT, AUDIT-C, CRAFFT and POSIT. *European addiction research* 2013;19(3):121-7. doi: 10.1159/000342331 [published Online First: 2012/11/28]
78. Collins RL, Parks GA, Marlatt GA. Social determinants of alcohol consumption: the effects of social interaction and model status on the self-administration of alcohol. *J Consult Clin Psychol* 1985;53(2):189-200. [published Online First: 1985/04/01]
79. Kivlahan DR, Marlatt GA, Fromme K, et al. Secondary prevention with college drinkers: Evaluation of an alcohol skills training program. *J Consult Clin Psych* 1990;58(6):805-10. doi: 10.1037/0022-006X.58.6.805
80. Berman AH, Bergman H, Palmstierna T, et al. Evaluation of the Drug Use Disorders Identification Test (DUDIT) in criminal justice and detoxification settings and in a Swedish population sample. *European addiction research* 2005;11(1):22-31. doi: 81413 [pii] 10.1159/000081413 [published Online First: 2004/12/21]
81. Hildebrand M. The psychometric properties of the Drug Use Disorders Identification Test (DUDIT): A review of recent research. *Journal of substance abuse treatment* 2015 a;53:52-59.
82. Sinadinovic K, Wennberg P, Berman AH. Population screening of risky alcohol and drug use via Internet and Interactive Voice Response (IVR): A feasibility and psychometric study in a random sample. *Drug and Alcohol Dependence* 2011;114(1):55-60. doi: https://doi.org/10.1016/j.drugalcdep.2010.09.004
83. Topp CW, Ostergaard SD, Sondergaard S, et al. The WHO-5 Well-Being Index: a systematic review of the literature. *Psychotherapy and psychosomatics* 2015;84(3):167-76. doi: 10.1159/000376585 [published Online First: 2015/04/04]
84. Love J, Andersson L, Moore CD, et al. Psychometric analysis of the Swedish translation of the WHO well-being index. *Quality of life research : an international journal of quality of life aspects of treatment, care and rehabilitation* 2014;23(1):293-7. doi: 10.1007/s11136-013-0447-0 [published Online First: 2013/06/08]
85. Strandberg AK, Skoglund C, Gripenberg J, et al. Alcohol and illicit drug consumption and the association with risky sexual behavior among Swedish youths visiting youth health clinics. *Nordic Studies on Alcohol and Drugs* Accepted
86. Brown BB, Clasen DR, Eicher SA. Perceptions of peer pressure, peer conformity dispositions, and self-reported behavior among adolescents. *Developmental Psychology* 1986;22(4):521-30. doi: 10.1037/0012-1649.22.4.521

For peer review only - http://bmjopen.bmj.com/site/about/guidelines.xhtml

87. Frost RO, Marten P, Lahart C, et al. The dimensions of perfectionism. *Cognitive Therapy and Research* 1990;14(5):449-68. doi: 10.1007/BF01172967
88. Rozental A, Shafran R, Wade T, et al. A randomized controlled trial of Internet-Based Cognitive Behavior Therapy for perfectionism including an investigation of outcome predictors. *Behaviour research and therapy* 2017;95:79-86. doi: 10.1016/j.brat.2017.05.015 [published Online First: 2017/06/07]
89. Stattin H, Kerr M. Parental monitoring: a reinterpretation. *Child development* 2000;71(4):1072-85. [published Online First: 2000/10/04]
90. Salari R, Thorell LB. Parental monitoring in late adolescence: Relations to ADHD symptoms and longitudinal predictors. *Journal of Adolescence* 2015;40:24.
91. Kerr M, Stattin H. What parents know, how they know it, and several forms of adolescent adjustment: further support for a reinterpretation of monitoring. *Dev Psychol* 2000;36(3):366-80. [published Online First: 2000/06/01]
92. Marlatt GA, Baer JS, Kivlahan DR, et al. Screening and brief intervention for high-risk college student drinkers: Results from a 2-year follow-up assessment. *J Consult Clin Psych* 1998;66(4):604-15.
93. Widmark E. Die theoretischen Grundlagen und die praktische Verwendbarkeit der gerichtlich-medizinischen Alkoholbestimmung. Berlin Wien: Urban und Schwarzenberg 1932.
94. Babor T, Higgins-Biddle J. Brief Intervention For Hazardous and Harmful Drinking: A Manual for use in Primary Care: World health Organization 2001.
95. Young RM, Hasking PA, Oei TP, et al. Validation of the Drinking Refusal Self-Efficacy Questionnaire--Revised in an Adolescent Sample (DRSEQ-RA). *Addictive behaviors* 2007;32(4):862-8. doi: 10.1016/j.addbeh.2006.07.001 [published Online First: 2006/08/22]
96. Gibbons RD, Hedeker D, DuToit S. Advances in analysis of longitudinal data. *Annu Rev Clin Psychol* 2010;6:79-107. doi: 10.1146/annurev.clinpsy.032408.153550
97. Cole HA, Prassel HB, Carlson CR. A Meta-Analysis of Computer-Delivered Drinking Interventions for College Students: A Comprehensive Review of Studies From 2010 to 2016. *J Stud Alcohol Drugs* 2018;79(5):686-96. [published Online First: 2018/11/14]
98. Berman AH, Gajecki M, Sinadinovic K, et al. Mobile Interventions Targeting Risky Drinking Among University Students: A Review. *Curr Addict Rep* 2016;3:166-74. doi: 10.1007/s40429-016-0099-6 [published Online First: 2016/04/05]

For peer review only

FIGURE LEGENDS

Figure 1 Study design

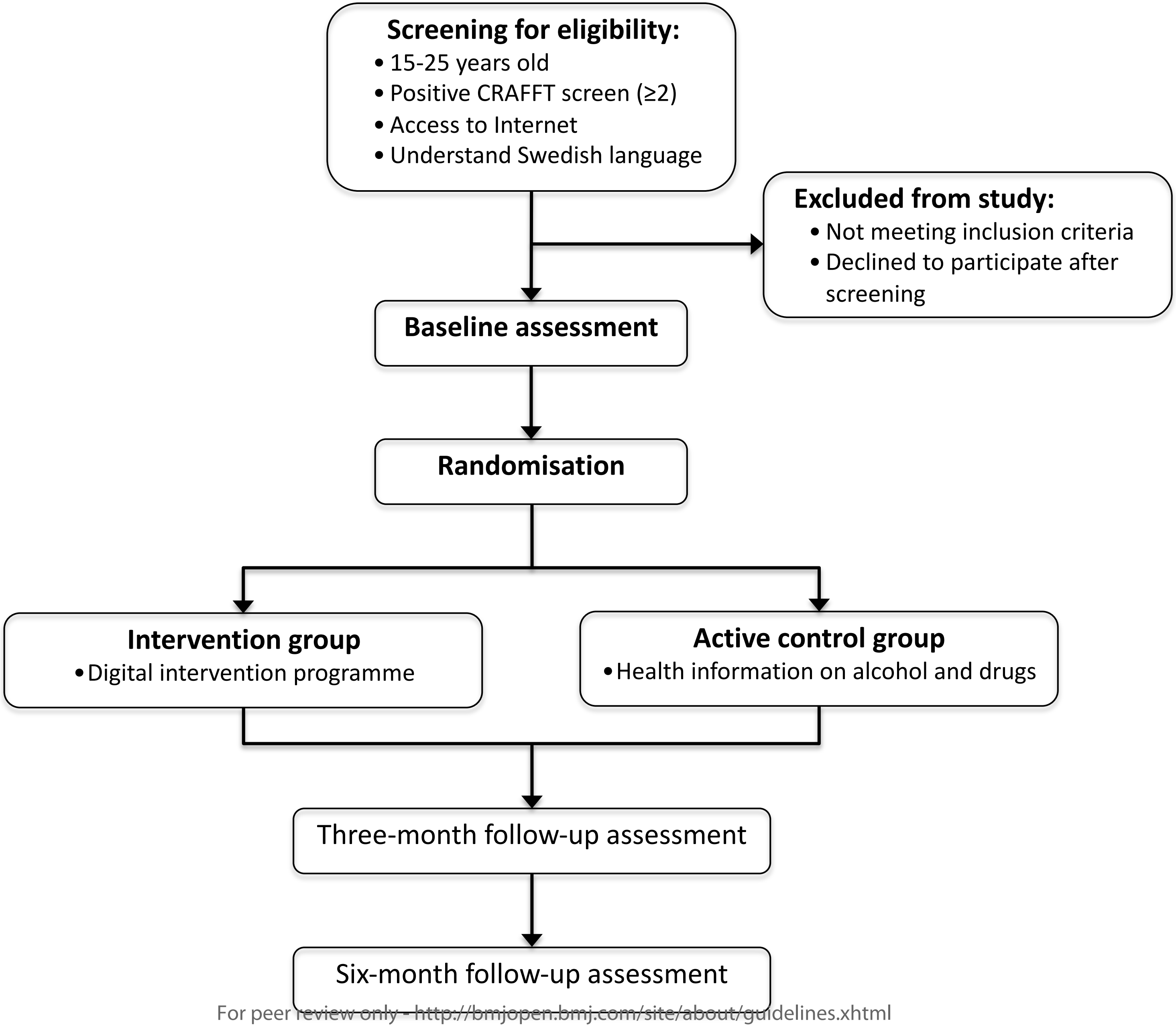
Screening for eligibility:
• 15-25 years old
• Positive CRAFFT screen (≥2)
• Access to Internet
• Understand Swedish language
Excluded from study:
• Not meeting inclusion criteria
• Declined to participate after screening
Baseline assessment
Randomisation
Intervention group
• Digital intervention programme
Active control group
• Health information on alcohol and drugs
Three-month follow-up assessment
Six-month follow-up assessment

BMJ Open

Effects of an automated digital brief prevention intervention targeting adolescents and young adults with risky alcohol and other substance use: Study protocol for a randomised controlled trial

Journal:	*BMJ Open*
Manuscript ID	bmjopen-2019-034894.R2
Article Type:	Protocol
Date Submitted by the Author:	12-Feb-2020
Complete List of Authors:	Kvillemo, Pia; Karolinska Institutet, STAD, Centre for Psychiatry Research, Department of Clinical Neuroscience, Karolinska Institutet & Stockholm Health Care Services, Region Stockholm, Norra Stationsgatan 69, SE-113 64<br>Strandberg, Anna; Karolinska Institutet, STAD, Centre for Psychiatry Research, Department of Clinical Neuroscience, Karolinska Institutet & Stockholm Health Care Services, Region Stockholm<br>Gripenberg, Johanna; Karolinska Institutet, STAD, Centre for Psychiatry Research, Department of Clinical Neuroscience, Karolinska Institutet & Stockholm Health Care Services, Region Stockholm<br>Berman, Anne; Karolinska Institutet, Centre for Psychiatry Research, Department of Clinical Neuroscience, Karolinska Institutet & Stockholm Health Care Services, Region Stockholm, Department of Psychology, Uppsala University, Uppsala<br>Skoglund, Charlotte; Karolinska Institutet, STAD, Centre for Psychiatry Research, Department of Clinical Neuroscience, Karolinska Institutet & Stockholm Health Care Services, Region Stockholm, Department of Pharmaceutical Biosciences, Uppsala University, Uppsala<br>Elgán, Tobias; Karolinska Institutet, STAD, Centre for Psychiatry Research, Department of Clinical Neuroscience, Karolinska Institutet & Stockholm Health Care Services, Region Stockholm
**Primary Subject Heading**:	Public health
Secondary Subject Heading:	Addiction, Medical education and training, Mental health, Sexual health
Keywords:	PSYCHIATRY, Substance misuse < PSYCHIATRY, Child & adolescent psychiatry < PSYCHIATRY, PREVENTIVE MEDICINE

SCHOLARONE™
Manuscripts

I, the Submitting Author has the right to grant and does grant on behalf of all authors of the Work (as defined in the below author licence), an exclusive licence and/or a non-exclusive licence for contributions from authors who are: i) UK Crown employees; ii) where BMJ has agreed a CC-BY licence shall apply, and/or iii) in accordance with the terms applicable for US Federal Government officers or employees acting as part of their official duties; on a worldwide, perpetual, irrevocable, royalty-free basis to BMJ Publishing Group Ltd ("BMJ") its licensees and where the relevant Journal is co-owned by BMJ to the co-owners of the Journal, to publish the Work in this journal and any other BMJ products and to exploit all rights, as set out in our licence.

The Submitting Author accepts and understands that any supply made under these terms is made by BMJ to the Submitting Author unless you are acting as an employee on behalf of your employer or a postgraduate student of an affiliated institution which is paying any applicable article publishing charge ("APC") for Open Access articles. Where the Submitting Author wishes to make the Work available on an Open Access basis (and intends to pay the relevant APC), the terms of reuse of such Open Access shall be governed by a Creative Commons licence – details of these licences and which Creative Commons licence will apply to this Work are set out in our licence referred to above.

Other than as permitted in any relevant BMJ Author's Self Archiving Policies, I confirm this Work has not been accepted for publication elsewhere, is not being considered for publication elsewhere and does not duplicate material already published. I confirm all authors consent to publication of this Work and authorise the granting of this licence.

For peer review only

Effects of an automated digital brief prevention intervention targeting adolescents and young adults with risky alcohol and other substance use: Study protocol for a randomised controlled trial

Authors

Pia Kvillemo (corresponding author)
STAD, Centre for Psychiatry Research,
Department of Clinical Neuroscience, Karolinska Institutet & Stockholm Health Care Services, Region Stockholm
Norra Stationsgatan 69, SE-113 64 Stockholm, Sweden
+46 (0)70 673 48 64
pia.kvillemo@ki.se

Anna K Strandberg
STAD, Centre for Psychiatry Research,
Department of Clinical Neuroscience, Karolinska Institutet, & Stockholm Health Care Services, Region Stockholm
Stockholm, Sweden

Johanna Gripenberg
STAD, Centre for Psychiatry Research,
Department of Clinical Neuroscience, Karolinska Institutet, & Stockholm Health Care Services, Region Stockholm
Stockholm, Sweden

Anne H Berman
Centre for Psychiatry Research,
Department of Clinical Neuroscience, Karolinska Institutet, & Stockholm Health Care Services, Region Stockholm
Stockholm, Sweden
Department of Psychology, Uppsala University, Uppsala, Sweden

Charlotte Skoglund
STAD, Centre for Psychiatry Research,
Department of Clinical Neuroscience, Karolinska Institutet, & Stockholm Health Care Services, Region Stockholm
Stockholm, Sweden
Department of Pharmaceutical Biosciences, Uppsala University, Uppsala, Sweden

Tobias H Elgán
STAD, Centre for Psychiatry Research,
Department of Clinical Neuroscience, Karolinska Institutet, & Stockholm Health Care Services, Region Stockholm
Stockholm, Sweden

Keywords
Motivational Interviewing, Decisional balance, Substance use, Web-based intervention, Normative feedback.

Wordcount
4654

For peer review only - http://bmjopen.bmj.com/site/about/guidelines.xhtml

Effects of an automated digital brief prevention intervention targeting adolescents and young adults with risky alcohol and other substance use: Study protocol for a randomised controlled trial

ABSTRACT

Introduction

Adolescence and young adulthood is a period in life when individuals may be especially vulnerable to harmful substance use. Several critical developmental processes are occurring in the brain, and substance use poses both short- and long-term risks with regard to mental health and social development. From a public health perspective, it is important to prevent or delay substance use to reduce individual risk and societal costs. Given the scarcity of effective interventions targeting substance use among adolescents and young adults, cost-effective and easily disseminated interventions are warranted. The current study will test the effectiveness of a fully automated digital brief intervention aimed at reducing alcohol and other substance use in adolescents and young adults aged 15-25 years.

Methods and analysis

A two-arm double-blind randomised controlled trial (RCT) design is applied to assess the effectiveness of the intervention. Baseline assessment, as well as three- and six-month follow-up, will be carried out. The aim is to include 800 participants with risky substance use based on the screening tool CRAFFT. Recruitment, informed consent, randomisation, intervention, and follow-up will be implemented online. The primary outcome is reduction in alcohol use, measured by AUDIT-C total score and average total drinks per week assessed by DDQ. Secondary outcomes concern binge drinking, frequency of alcohol consumption, amount of alcohol consumed a typical day when alcohol is consumed, other substance use, mental health, sexual risk behaviours, and perceived peer pressure. Moreover, the study involves analyses of potential moderators including perfectionism, openness to parents, help-seeking, and background variables.

For peer review only

For peer review only - http://bmjopen.bmj.com/site/about/guidelines.xhtml

Ethics and dissemination

The study was approved by the Swedish Ethical Review Authority (no. 2019-03249). The trial is expected to expand the knowledge on digital preventive interventions for substance using adolescents and young adults. Results will be disseminated in research journals, at conferences and via the media.

Registration

24/09/2019, ISRCTN91048246.

Key words

Motivational Interviewing, Decisional balance, Substance use, Web-based intervention, Normative feedback.

ARTICLE SUMMARY

Strengths and limitations of this study

- A double-blind randomised controlled trial (RCT) is considered to be the most robust experimental design, enabling causal inferences, controlling for selection bias and participant allocation bias.
- An active control condition, blinded to the study condition, controls for expectation, detection and performance bias.
- Analysis of several potential moderators, i.e., perfectionism, openness to parents, help-seeking and background variables will contribute to the understanding of possible effects.

A convenience sample may reduce generalizability due to possible selection bias at recruitment.

For peer review only - http://bmjopen.bmj.com/site/about/guidelines.xhtml

INTRODUCTION

Substance use is a major public health concern causing individual suffering as well as societal costs.[1-3] Substance use in childhood and younger ages is particularly harmful since the brain is undergoing critical development during this time in life, which makes it more vulnerable to addictive substances.[4 5] In adolescence, some individuals are especially prone to various risk behaviours, increasing the risk of e.g., substance use.[6] Additionally, having substance using peers is a prominent risk factor for one's own substance use at this age, since peers are increasingly important in teenagers' social life.[7] Thus, the initiation of substance use often occurs during this period.[6] After initiation, the use of substances frequently increases during adolescence and young adulthood, posing a number of risks for the individual.[2 8 9] Early onset of substance use implies a risk of severe adverse effects on psychological and physiological development.[2 8 9] Hazardous use of alcohol, cannabis and other substances can lead to chronic problematic consumption patterns and addiction that can significantly influence the developmental trajectory during the transition from childhood to adulthood.[10] More acute consequences include, e.g., mental or psychiatric problems, problems of academic adjustment,[11] accidents,[12] and problems with the police or legal authorities.[13 14] Risk behaviours, such as substance use, binge drinking, intoxication, and sexual risk behaviour often co-occur, and the use of illicit substances, is e.g., often accompanied by alcohol consumption.[1 2 11 15-19] Poly-substance use, i.e., simultaneous use of different substances, may imply an increased risk of various negative outcomes, e.g., mental health problems, shown to increase in magnitude and over time, with the number of substances used.[20] The well-established association between substance use and mental health problems is bidirectional, sometimes manifested in self-medication for mental health problems, and sometimes as an increase in such problems due to substance use.[21] While most of prior research shows that low socio-economic standard is a risk factor for substance use and related problems,[22 23] the current study is informed by recent research on mechanisms involved in adolescence alcohol and other substance use among upper secondary school students from affluent areas, showing associations between internalizing symptoms and substance use,[24 25] as well as associations

For peer review only - http://bmjopen.bmj.com/site/about/guidelines.xhtml

between achievement pressures (particularly excessive perfectionistic strivings), and isolation from parents (particularly low perceived closeness to mothers).[24 26] The latter finding is in line with previous research showing that a positive parent–child relationship is a protective factor related to lower likelihood of adolescence substance use.[27 28]

Although alcohol use has declined among adolescents during recent years, some individuals drink more and binge drinking is still highly prevalent among young adults, where the largest proportion of alcohol risk consumers is still found.[29 30] Moreover, a high prevalence of substance use and poly-substance use among young people in Europe was recognized in the EU Drug Strategy 2005–2012, which calls for action. [31] Also, established cannabis users among Swedish upper secondary school students tend to use cannabis more often than before, according to recent survey data.[32] In light of these circumstances, it is of utmost importance to target and tailor attractive preventive interventions for adolescents and young adults. One way to reach large groups of young people is to disseminate digital interventions online (i.e., via the Internet). In Sweden, 98 percent of the population has access to the Internet at home and virtually 100 percent of 16-25 year-olds use the Internet in mobile phones, tablets or computers.[33] Digital interventions have several advantages over traditional ones. They may reduce the stigma around risk behaviours, they are accessible at any time and place, and can be cost-effective because minimal staff resources are used for implementation, especially if they are fully automated.[34 35] Digital interventions may thus be particularly suitable for adolescents and young adults.[36] To date, digital prevention programmes targeting alcohol and other substance use among adolescents and young adults that meet scientific evaluation criteria are scarce. An early example is eScreen.se, offering screening for alcohol and drug use with personalized feedback.[37] A more recent example is a fully automated brief motivational intervention for substance-using 16-18 year-olds, tested in an RCT in four European countries,[38-40] where our research group was one of the partners. The intervention, named WISEteens, relied on Motivational Interviewing (MI)[41] and models of Social Influence.[42 43] The goal of the MI approach was to enhance motivation to change by exploring and resolving ambivalence about substance-related behaviours. An important element in the intervention was personalized feedback about one's substance use behaviours in relation to normative comparisons, as personalized feedback is perceived as more relevant for changing behavior than more general information.[44] The normative feedback included information about how a specific reference group actually consumed substances, in order to correct participants' "inflated perception",[45] which has proven effective for reduction of alcohol consumption in young adults in previous meta-analyses.[46 47] For adolescents and young adults, a normative feedback approach may be particularly appealing, assuming they are curious about how their substance consumption compares to their peers.[48] Likewise, an important element of the intervention was a focus on substance-related social norms and

For peer review only - http://bmjopen.bmj.com/site/about/guidelines.xhtml

training on how to avoid social high-risk situations and how to resist peer pressure; i.e., raising refusal self-efficacy.[49] In line with the social influence hypothesis stating that a prominent risk factor for substance use in adolescence is the influence of substance using peers,[7] targeting peer pressure in brief digital interventions has been an important element for reducing alcohol use among adolescents.[50] WISEteens demonstrated significant between-group effects for alcohol use, indicating that a targeted brief motivational intervention in a fully automated digital format can be effective to reduce drinking and lower barriers for accessing substance use service in hazardous drinking adolescents.[38]

Digital interventions, often delivered online, generally offer adaptations of evidence based face-to-face interventions, e.g., MI. The effects of MI on several health-related behaviours have been evaluated in a number of other studies, especially among adults. [51] Among adolescents, systematic reviews and meta-analyses on MI for various health-related behaviours show mixed results. A review of six studies of MI for reducing alcohol consumption in the emergency room setting suggested that MI was at least as effective as other brief interventions in the same setting.[52] Another review of 24 studies of different brief interventions for reducing alcohol consumption and alcohol-related problems among adolescents showed a significant effect that persisted up to one year compared to control interventions, with greater effects for MI. Effects were consistent over diverse settings and particularly effective components included decisional balance (juxtaposition of pros and cons of change)[53] and goal-setting[54]. Regarding MI for illicit drug use among adolescents, a review of 10 studies showed no effects on drug use behaviors; however, changes in attitudes towards drug use were found, which could be translated into intentions to change behaviors [53]. A scoping review concerning MI for reducing sexual risk behaviors among adolescents identified 29 unique studies with varying designs and conceptualizations of MI and specific risk behaviours, making it difficult to generalize regarding outcomes but indicating the need of more research.[55] MI has most commonly been delivered as an individual face-to-face intervention[51], but has also been provided in other forms, e.g., via telephone or digitally, with various results on e.g., substance use behaviours.[56 57] Digital programmes based on MI have demonstrated effect in the form of reduced alcohol consumption among young adults.[58 59] Additionally, a combination of screening with a short intervention has shown similar effects on alcohol consumption among adolescents.[60] Brief motivational interventions are empirically supported individual level interventions for reducing alcohol consumption.[45 61] Such interventions have in the last decades also been digitally provided.[62 63] Digital interventions can be provided with or without human guidance, i.e., more or less automated.[64] Previous research has demonstrated that even fully automated interventions can reduce alcohol-related problems for young people with risky alcohol consumption up to 12 months after implementation,[59 65] and indicated potential effect on cannabis use in certain groups.[66 67] Although various digital substance use prevention

For peer review only - http://bmjopen.bmj.com/site/about/guidelines.xhtml

interventions have been developed and tested with promising results,[38 68] studies on the effects of fully automated digitally delivered and MI-based substance use prevention programmes for adolescents and young adults are still few[68] and more well-designed studies are warranted in order to obtain evidence for potential effects.

Aim

The study described in this protocol builds on results previously obtained in a European study of a digital brief MI-based substance use prevention intervention (WISEteens), delivered online to adolescents in a fully automated form.[38] In this study, the effectiveness of a modified version of this intervention, with new graphics and population based feedback-generating data on alcohol consumption covering all included age groups, will be tested across a range of outcome measures among 15-25 year-olds with hazardous alcohol or other substance use. The primary aim is reduction in frequency and quantity of substance use.

Rationale and hypothesis

The current study will add to existing evidence regarding the effectiveness of digital interventions aimed at reducing alcohol and other substance use among young people. We hypothesize that participants in the intervention group will report a significant reduction in substance use (primarily alcohol use), compared to an active control group receiving health information about various substances. A number of potential moderators will be assessed to confirm or contradict previous research on factors influencing the development of substance use patterns over time, and effects of prevention interventions in different target groups.

METHODS AND ANALYSIS

In order to evaluate the effectiveness of the digital screening and brief motivational intervention, a double-blind two-arm RCT study design is planned with baseline assessment at study entry and follow-up assessment at three and six-months across a number of outcome variables. Figure 1 displays the trial design. We will also explore and test for moderator effects. To outline and report the current study we used SPIRIT reporting guidelines.[69]

For peer review only

Recruitment

The target group for the current study is adolescents and young adults aged 15-25 years with self-reported substance use, a capacity to understand the Swedish language, and access to the Internet. Based on a power calculation to detect a small effect size (Cohen's d=0.2),[70] the aim is to include 800 participants.[71] The participants will be recruited during January-October 2020 by offline marketing at youth health clinics, other health care units for youth, and at upper secondary schools in Stockholm county, as well as by social networks, using online banner advertisements. This will result in a convenience sample from the general Swedish population.[44] Results from follow-up measures are to be completely received in 2021. If sufficient number of participants have not been recruited according to plan, we will extend the recruitment period and intensify the advertising by increasing visibility on social platforms. To enhance participation and follow-up rates, an incentive in the form of a movie ticket, with a value up to 15 Euros, for each completed follow-up assessment will be provided, i.e., up to 3 tickets. Up to three reminders will be sent out if participants do not respond to follow-up assessments.

Screening and informed consent

Potential participants will be guided to an online landing page with screening by an adapted version of the screening tool for use of alcohol and other substances, CRAFFT (Car, Relax, Alone, Forget, Friends, Trouble).[72] This six-item screening tool has demonstrated criterion validity and appropriateness for identifying substance-related problems among adolescents.[73-75] The primary eligibility criterion for participating in the study will be a score of 2 or more on the CRAFFT, since this value has shown satisfactory sensitivity for identifying substance use problems.[72] Those who score 2 or higher will be offered participation in the study and given information on confidentiality, voluntariness of participation, and human subject protections.[44] They will also be provided with contact information on suitable counselling service providers. Those who agree to participate will be asked to give digital informed consent.

Measures

All assessment will occur online. The selection of measures and associated instruments is based on the aim of the study and the theoretical base concerning factors influencing substance use. Based on results

For peer review only - http://bmjopen.bmj.com/site/about/guidelines.xhtml

from the previous European WISEteens study,[38] showing significant effects on alcohol consumption but not on other substance use, the primary outcome is alcohol use, measured at three- and six-month follow-ups. Secondary outcomes include binge drinking, frequency of alcohol consumption, amount of alcohol consumed a typical day when alcohol is consumed at three- and six-months follow-ups, other substance use, mental health, sexual risk behaviours, and perceived peer pressure at three and six-month follow-ups. The moderating variables include perfectionism, openness to parents, help-seeking due to mental health problems, and socio-demographic and personal characteristics, (i.e. sex, age, residence, occupation, school performance (for students), and parents' education).

Alcohol use, including the primary outcome, will be measured using two instruments. One of them is the short version of the Alcohol Use Disorders Identification Test (AUDIT-C),[76] measuring frequency and amount of consumed alcohol and frequency of binge drinking, providing a widely used and valid index sum score for problematic alcohol use among adolescents.[75 77] The other is the Daily Drinking Questionnaire (DDQ),[78] measuring a variety of parameters of alcohol use in a typical week. Previous research has demonstrated that the DDQ is highly correlated with other measures of self-reported alcohol consumption.[79] The primary outcome will be measured by AUDIT-C total score and average total drinks per week, assessed by DDQ. The two primary outcome measures complement one another in that the AUDIT-C primarily offers an indication of hazardous or harmful use,[80] whereas the DDQ quantity measure will indicate the level of alcohol consumption in grams per week. Other substance use will be measured by a short version of the Drug Use Disorder Identification Test (DUDIT),[81] including the first four items (DUDIT-C) which assess frequency of consumption of drugs other than alcohol, frequency of different types of drugs other than alcohol used at the same occasion (1 = never to 5 = four or more times a week), and number of occasions when drugs other than alcohol are consumed on a typical day of drug use (1 = zero to 5 = seven or more). The DUDIT has been found to be effective in screening for drug-related problems in clinically selected groups,[82] and both the DUDIT and the DUDIT-C have proven useful in the context of public health surveys in Sweden.[81 83] Mental health will be measured by The WHO-5 Well-Being Index, which has demonstrated validity both as a screening tool for depression and as an outcome measure in clinical trials.[84] This scale has been successfully applied across a wide range of study fields, translated into more than 30 languages, including Swedish,[85] and found psychometrically sound. Changes in sexual risk behaviours will be measured by multiple choice questions on sexual behaviour under the influence of alcohol and/or other substances and unprotected sex, previously used in a survey among Swedish visitors at youth health clinics in Stockholm county.[86] Changes in perceived peer pressure will be

For peer review only - http://bmjopen.bmj.com/site/about/guidelines.xhtml

measured by two items retrieved and adapted from the Peer pressure inventory.[87] Perfectionism will be measured by two subscales of the Frost Multidimensional Perfectionism Scale (FMPS).[88 89] Openness to parents will be measured among participants who are up to 20 years old with questions about disclosure from Stattin & Kerr,[90-92] slightly modified into statements to be compatible with the digital online format. Help-seeking due to mental health problems will be measured by dichotomous questions regarding if, and to what healthcare provider, the participant has turned for help. Additionally, background data will be asked for using multiple choice questions: sex (man, women, other), age (15, 16…25), residence (name of municipality), occupation (secondary school, upper secondary school, university, not student, working, practicing, unemployed, other), school performance (merit value for students), and parents' education (secondary school, upper secondary school, university, don't know).

Randomisation

The study is double-blind, thus neither the participant nor the researchers will know which participant is allocated to the intervention or to the active control condition. After baseline assessment, the participants will be automatically randomised to one of the two study groups by a computer programme using an unrestricted randomisation protocol. Participants will then be informed about the name of their programme and given access immediately.

The intervention

Participants allocated to the intervention group will initially be asked to give additional information on alcohol and other substance use and to state their body weight, in order to generate personalized feedback on substance use behaviour and to estimate alcohol blood concentration on a typical drinking occasion.

Description of the intervention content

The intervention is interactive, digitally delivered online in a fully automated form, and requires approximately 20 minutes to complete. Personalized feedback is given to the participants based on their responses to previous assessment and suggestions on how to respond to this feedback are provided. This interactivity imitates a face-to face "dialogue" with techniques from MI such as an empathic approach, rolling with resistance, aiming at creating a dissonance between actual and desired behaviour, raising self-

For peer review only - http://bmjopen.bmj.com/site/about/guidelines.xhtml

efficacy, and at the same time avoiding argumentation.[57] The intervention consists of three main components outlined below, and additional health related information.

Personalized feedback

The personalized feedback includes an estimation of the participants' blood-alcohol-concentration (BAC) and information on the associated risks concerning the participants' heaviest drinking episode during the past 30 days. The value will be based on a measure of Peak Drinking Quantity[93] and estimated using the Widmark-formula, which takes into account weight and sex.[94] The participants will receive graphed feedback regarding number of standard alcohol units per week that they think their peers consume (descriptive norms), as well as the participant's individual levels of consumption in relation to comparative data (actual drinking levels) from a reference group. Comparative data (AUDIT-C scores) will be taken from alcohol prevalence estimates found in a nationally representative sample of 16-25 year-olds undertaken by the Public Health Agency of Sweden. Comparative feedback will be available for drinking but not for substances other than alcohol.

Interactive MI-based exercises

The exercises provided in the current intervention build on the assumption that participants may hold certain levels of ambivalence about their current substance use, and that if they are willing to make a change they may not know how to, or may not be confident that they are able to.[41] Therefore, the intervention uses importance and confidence rulers with a short summary and feedback to encourage participants to reflect on personal reasons for change and explore personal strengths and ability to change. Furthermore, the programme provides a decisional balance to pick up and graphically illustrate potential levels of ambivalence by offering the participants a list of possible pros and cons regarding the decision to change their current substance use.[57 95] Participants are instructed to choose statements that apply to them and are presented with the resulting balance sheet of their personal comparative potential gains and losses.

For peer review only - http://bmjopen.bmj.com/site/about/guidelines.xhtml

Practical advice

The intervention focuses on raising self-efficacy for being able to avoid drinking in social situations, if desirable.[50] The participant will be asked to select three among 12 provided drinking situations that they consider most tempting and rank them. The situations are adapted from the adolescent version of the Drinking Refusal Self-efficacy Questionnaire (DRSEQ-RA).[96] According to the selection, a number of strategies will be offered for each of the selected drinking situations to provide participants with a tool kit necessary for engaging in and maintaining their behavioural goal.

Health related information connected to substance use

Finally, the intervention programme includes health related information associated with substance use. The information is provided optionally throughout the program behind “read more” buttons and also at the end of the programme. The information contains statements regarding risks connected to substance use that the participant can reflect on, as well as optional links for more information. There are also two FAQ-sections about alcohol and cannabis, respectively, as well as information about how different substances may affect the individual, both physically and mentally. Finally, the information also contains cases, were the participant can read about some typical young persons who have used substances, why they used and what negative effects they have noted, and why they chose to stop using.

The control condition

The control group will receive the same general health related information as the intervention group, i.e., the additional information which is connected to the intervention programme.

Statistical analysis

Randomisation-checks of baseline variables regarding alcohol and other substances use and psychological state will be conducted using multivariate analyses of variance MANOVA. To test the effectiveness of the intervention, we will assess whether participants in the intervention group to a greater extent report decreased substance use, sexual risk behaviours, perceived peer-pressure, and improved mental health after three and six months, respectively, compared to participants in the active control group. Data analyses will consist of comparing outcome measurements with regards to within-group and between-group differences according to the intention-to-treat (ITT) principle in the primary analysis, accounting for all included participants regardless of whether or not they completed follow-up assessments. We will also perform per protocol analyses. The reason for choosing ITT in the primary analysis is the ambition to

For peer review only - http://bmjopen.bmj.com/site/about/guidelines.xhtml

maintain the power of the study and also to avoid biased results due to selection of those completing all follow-ups, as they may have special characteristics not representative for the whole study group, which might influence study results. The main analysis of effectiveness will use mixed effects regression models, which can be applied to both continuous and categorical outcomes and also non-normally distributed outcomes. Also, mixed-effects regression models are robust to missing data in longitudinal studies.[97] In the current study, we assume a quite large dropout rate, based on previous research. For example, a meta-analysis comprising 17 studies of unsupported (fully automated) interventions for depression showed 74% dropout rate at post-treatment.[98] Moreover, we expect data missing at random (MAR), which can be handled using mixed effects regression models. Separate models will be run to test each outcome, i.e., alcohol consumption, binge drinking, frequency of alcohol consumption, amount of alcohol consumed a typical day when alcohol is consumed at three- and six-months follow-ups, other substance use, mental health, sexual risk behaviours and perceived peer pressure. Effect sizes will be calculated separately for the two measures used for the primary outcome variable (AUDIT-C and DDQ). Potential moderators, including perfectionism, openness to parents, help-seeking due to mental health problems, and socio-demographic and personal characteristics, (i.e. sex, age, residence, occupation, school performance (for students), and parents' education) will serve as covariates in analyses of intervention effects. If moderator effects are found, we will carry out post-hoc analyses stratified by the detected variable.

Patient and public involvement statement

Patients and the public were not involved in the design and planning of the study, except for persons at the age of the target group reading manuscripts for the revised version of WISEteens and also participating in a pilot-test.

ETHICS AND DISSEMINATION

The current study was approved by the Swedish Ethical Review Authority (no. 2019-03249) and registered 24/09/2019, pre-results, on IRCTN, ID: ISRCTN91048246. Any important protocol modifications will be reported to IRCTN. For inclusion, all participants must give informed consent online prior to participation in the study. In order to participate, the participants will need to state a username (which may be fictitious or a pseudonym) and an email address. All data collection will be done without collecting personal identification information, only personal e-mail addresses. The e-mail address will be used to connect data from the baseline measurement to the follow-up measurements. At a later stage, the

For peer review only - http://bmjopen.bmj.com/site/about/guidelines.xhtml

raw data file will be anonymized and each person assigned a number instead of the e-mail address. The data will be stored in line with routines for handling and storing research data at Karolinska Institutet. All data is handled confidentially and will not be forwarded to third parties. Participating in the present study means that the participants need to reflect on their alcohol consumption and other substance use. In addition, questions will be asked concerning personal circumstances, including the participants' mental health and family relationships. These issues may be perceived as somewhat unpleasant. However, in the information that potential participants receive, they are informed that participation is voluntary and that they at any time can end their participation without explaining why. In addition, there will be reference to other types of official support (web pages and telephone numbers), if the advertisement or participation leads to concern about own substance use or related problems. Any issues brought up by the participants during the study will be documented and handled properly. Moreover, the research team includes professionals, such as a psychiatrist and a nurse, with possibility to refer participants to health care clinics if needed. The project's basic hypothesis is that the intervention will have positive effects, with regard to alcohol and other substance use among young people with risk use, and potential benefit may include decreased or ceased risk behaviours. Overall, the benefit for the research persons is considered to exceed any risk of discomfort. Results will be disseminated in scientific peer reviewed journals, at conferences and via the media.

DISCUSSION OF STRENGTHS AND LIMITATIONS

The current study has a number of strengths. The protocol describes a two-arm double-blind RCT, considered to be the most robust experimental design, controlling for selection bias and participant allocation bias and with the possibility to make causal inferences. Moreover, an active control condition, blinded to the study condition, controls for expectation, detection and performance bias. The collection and analysis of information on potential moderators, allowing for control of these factors, facilitates the understanding of the possible effects. Thus, the present study will contribute to the literature on digital substance use prevention interventions among adolescents and young adults in several ways. The choice to study a digital intervention can from a public health perspective be regarded as positive. Digital brief interventions have several advantages over face-to-face approaches, e.g., the reduction of stigma around help-seeking for substance use and easy dissemination to large groups of people, which makes them cost-effective. Moreover, digital interventions have previously proven effective in addressing alcohol and other substance use in the general population and some studies have shown promising results also among adolescents and young adults.[99 100] The current intervention is well grounded in theory and incorporates elements of MI and social influence theory that has been shown to be effective in reducing problematic

For peer review only - http://bmjopen.bmj.com/site/about/guidelines.xhtml

substance use in prior research.[57] Importantly, there are also some limitations to the current study. One limitation concerns selection bias and thereby external validity, as recruitment requires either that individuals click on our ads at social media to be considered for inclusion, or that they attend upper secondary schools, or have been in contact with existing clinics, aware of the opportunity to participate in the study. Thus, our participants may have certain personality traits, or are especially prone to help-seeking compared to a broader audience. The exclusion of people not understanding Swedish is also a limitation with regard to generalizability.

STATEMENTS

Author contributions

PK, AKS, JG, and THE obtained funding for the study. PK, THE, and CS further developed/modified the intervention. THE designed the study with contribution from PK, AKS, JG, AHB, and, CS. PK wrote this paper. All authors commented on successive manuscript drafts and approved the final version of the manuscript.

Funding statement

The work was supported by the National Public Health Agency of Sweden (grant no. 02299-2016-6.2) and the Alcohol Research Council of the Swedish Alcohol Retailing Monopoly (grant no. 2018-0016). Author AHB was supported by the Swedish Research Council (grant no. K2012-61P-22131-01-6). The funding bodies had no role in study design, data collection, analysis, data interpretation or writing manuscripts.

Data statement

Collected data will be available from the Centre for Psychiatry Research, a collaboration between the Karolinska Institute and Region Stockholm, but restrictions apply to their availability, as they were used under ethical permission for the current study, and so are not publicly available. However, data are available from the authors upon reasonable request and with permission from the Centre for Psychiatry Research.

For peer review only - http://bmjopen.bmj.com/site/about/guidelines.xhtml

For peer review only

Competing interests

The authors declare no competing interests.

Acknowledgments

The authors would like to thank Dr. Silke Diestelkamp and Dr. Christiane Baldus for invaluable help in the process of building the Swedish version of the WISEteens programme.

For peer review only

REFERENCES

1. Dawson DA, Goldstein RB, Chou SP, et al. Age at first drink and the first incidence of adult-onset DSM-IV alcohol use disorders. *Alcoholism Clinical and Experimental Research* 2008;32(12):2149-60.
2. Hibell B, Guttormsson U, Ahlström S, et al. The 2007 ESPAD Report: Substance use among students in 35 European countries. ESPAD, The European School Survey Project on Alcohol and Other Drugs. Stockholm, Sweden, 2009.
3. Anderson P. Global alcohol policy and the alcohol industry. *Curr Opin Psychiatry* 2009;22(3):253-7. doi: 10.1097/YCO.0b013e328329ed75
4. Chambers RA, Taylor JR, Potenza MN. Developmental neurocircuitry of motivation in adolescence: a critical period of addiction vulnerability. *The American journal of psychiatry* 2003;160(6):1041-52. doi: 10.1176/appi.ajp.160.6.1041 [published Online First: 2003/06/05]
5. Barron S, White A, Swartzwelder HS, et al. Adolescent vulnerabilities to chronic alcohol or nicotine exposure: findings from rodent models. *Alcohol Clin Exp Res* 2005;29(9):1720-5. [published Online First: 2005/10/06]
6. Balogh KN, Mayes LC, Potenza MN. Risk-taking and decision-making in youth: relationships to addiction vulnerability. *Journal of behavioral addictions* 2013;2(1) doi: 10.1556/jba.2.2013.1.1 [published Online First: 2013/12/03]
7. Oetting ER, Beauvais F. Peer cluster theory: Drugs and the adolescent. *Journal of Counseling & Development* 1986;65(1):17-22. doi: 10.1002/j.1556-6676.1986.tb01219.x
8. Arria AM, Caldeira KM, Bugbee BA, et al. Marijuana use trajectories during college predict health outcomes nine years post-matriculation. *Drug and alcohol dependence* 2016;159:158-65. doi: 10.1016/j.drugalcdep.2015.12.009 [published Online First: 2015/12/23]
9. Van Der Vorst H, Vermulst AA, Meeus WH, et al. Identification and prediction of drinking trajectories in early and mid-adolescence. *Journal of clinical child and adolescent psychology : the official journal for the Society of Clinical Child and Adolescent Psychology, American Psychological Association, Division 53* 2009;38(3):329-41. doi: 10.1080/15374410902851648 [published Online First: 2009/05/14]
10. R M, E K, P M, et al. Impact of alcohol consumption on young people: A systematic review of published reviews. Newcastle University, 2009.
11. Hicks RD, Bemis Batzer G, Bemis Batzer W, et al. Psychiatric, Developmental, and Adolescent Medicine Issues in Adolescent Substance Use and Abuse. *Adolesc Med* 1993;4(2):453-68.
12. White V, Azar D, Faulkner A, et al. Adolescents' alcohol use and strength of policy relating to youth access, trading hours and driving under the influence: findings from Australia. *Addiction* 2018;113(6):1030-42. doi: 10.1111/add.14164
13. Arendt M, Rosenberg R, Foldager L, et al. Cannabis-induced psychosis and subsequent schizophrenia-spectrum disorders: follow-up study of 535 incident cases. *The British journal of psychiatry : the journal of mental science* 2005;187:510-5. doi: 10.1192/bjp.187.6.510 [published Online First: 2005/12/02]
14. Flory K, Lynam D, Milich R, et al. Early adolescent through young adult alcohol and marijuana use trajectories: early predictors, young adult outcomes, and predictive utility. *Development and psychopathology* 2004;16(1):193-213. [published Online First: 2004/04/30]
15. Nilses C, Blom H, Heimer G, et al. 40 procent av gymnasieelever riskbrukar alkohol. *Läkartidningen* 2011;34(108):1156-559.
16. Bonomo Y, Coffey C, Wolfe R, et al. Adverse outcomes of alcohol use in adolescents. *Addiction* 2001;96(10):1485-96.
17. Guttmannova K, Hill KG, Bailey JA, et al. Examining explanatory mechanisms of the effects of early alcohol use on young adult alcohol dependence. *J Stud Alcohol Drugs* 2012;73(3):379-90.

For peer review only - http://bmjopen.bmj.com/site/about/guidelines.xhtml

18. Miller JW, Naimi TS, Brewer RD, et al. Binge drinking and associated health risk behaviors among high school students. *Pediatrics* 2007;119(1):76-85.
19. Chick CF, Reyna VF. A fuzzy trace theory of adolescent risk taking: Beyond self-control and sensation seeking. In: V. Reyna SC, M. Dougherty, & J. Confrey (Eds.), ed. The Adolescent Brain Washington, DC: American Psychological Association. 2012.
20. Burdzovic Andreas J, Lauritzen G, Nordfjærn T. Co-occurrence between mental distress and poly-drug use: A ten year prospective study of patients from substance abuse treatment. *Addict Behav* 2015;48:71-78. doi: https://doi.org/10.1016/j.addbeh.2015.05.001
21. Hodgins S, Tengström A, Bylin S, et al. Consulting for substance abuse: Mental disorders among adolescents and their parents. *Nordic Journal of Psychiatry* 2007;61(5):379-86. doi: 10.1080/08039480701643423
22. Swift W, Coffey C, Carlin JB, et al. Adolescent cannabis users at 24 years: trajectories to regular weekly use and dependence in young adulthood. *Addiction* 2008;103(8):1361-70. doi: 10.1111/j.1360-0443.2008.02246.x [published Online First: 2008/10/16]
23. Von Sydow K, Lieb R, Pfister H, et al. What predicts incident use of cannabis and progression to abuse and dependence? A 4-year prospective examination of risk factors in a community sample of adolescents and young adults. *Drug and Alcohol Dependence* 2002;68:49–64.
24. Låftman SB, Almquist YB, Östberg V. Students' accounts of school-performance stress: a qualitative analysis of a high-achieving setting in Stockholm, Sweden. *Journal of Youth Studies* 2013;16(7):932-49. doi: 10.1080/13676261.2013.780126
25. Hiltunen L. Lagom perfekt. Erfarenheter av ohälsa bland unga tjejer och killar. Linnéuniversitetet, 2017.
26. Luthar SS, Becker BE. Privileged but pressured? A study of affluent youth. *Child development* 2002;73(5):1593-610. [published Online First: 2002/10/04]
27. Ashby Wills T, Yaeger AM. Family Factors and Adolescent Substance Use: Models and Mechanisms. *Current Directions in Psychological Science* 2003;12(6):222-26. doi: 10.1046/j.0963-7214.2003.01266.x
28. Wills TA, Cleary SD. How are social support effects mediated? A test with parental support and adolescent substance use. *Journal of Personality and Social Psychology* 1996;71(5):937-52. doi: 10.1037/0022-3514.71.5.937
29. Guttormsson U, Gröndal M. Befolkningens självrapporterade alkoholvanor 2004-2017 Stockholm: CAN, 2018.
30. Lees B, Mewton L, Stapinski L, et al. Binge drinking in young people: protocol for a systematic review of neuropsychological, neurophysiological and neuroimaging studies. *BMJ Open* 2018;8(7):e023629. doi: 10.1136/bmjopen-2018-023629
31. European Union Drugs Strategy 2013–2020, 2013.
32. Zetterqvist ME. Skolelevers drogvanor 2018. Stockholm: Centralförbundet för alkohol- och narkotikaupplysning (CAN) 2018.
33. Foundation TSI. Svenskarna och internet 2018 (The Swedes and the Internet 2018) (In Swedish) 2018 [Available from: https://2018.svenskarnaochinternet.se/allmant-om-internetutvecklingen/ accessed 2019-06-25.
34. Murray E. Web-Based Interventions for Behavior Change and Self-Management: Potential, Pitfalls, and Progress. *Med 20* 2012;1(2):e3. doi: 10.2196/med20.1741
35. Riper H, Blankers M, Hadiwijaya H, et al. Effectiveness of Guided and Unguided Low-Intensity Internet Interventions for Adult Alcohol Misuse: A Meta-Analysis. *PLOS ONE* 2014;9(6):e99912. doi: 10.1371/journal.pone.0099912

For peer review only - http://bmjopen.bmj.com/site/about/guidelines.xhtml

36. Spijkerman R, Roek AEM, Vermulst A, et al. Effectiveness of a Web-Based Brief Alcohol Intervention and Added Value of Normative Feedback in Reducing Underage Drinking: A Randomized Controlled Trial. *J Med Internet Res* 2010;12(5):e65. doi: 10.2196/jmir.1465
37. Sinadinovic K, Berman AH, Hasson D, et al. Internet-based assessment and self-monitoring of problematic alcohol and drug use. *Addictive behaviors* 2010;35(5):464-70. doi: 10.1016/j.addbeh.2009.12.021 [published Online First: 2010/01/23]
38. Arnaud N, Baldus C, Elgan TH, et al. Effectiveness of a Web-Based Screening and Fully Automated Brief Motivational Intervention for Adolescent Substance Use: A Randomized Controlled Trial. *J Med Internet Res* 2016;18(5):e103. doi: 10.2196/jmir.4643
39. Arnaud N, Bröning S, Drechsel M, et al. Web-based screening and brief intervention for poly-drug use among teenagers: study protocol of a multicentre two-arm randomized controlled trial. *BMC Public Health* 2012;12:826. doi: 10.1186/1471-2458-12-826 [published Online First: 2012/09/28]
40. Arnaud N, Baldus C, Elgán TH, et al. Moderators of Outcome in a Web-Based Substance Use Intervention for Adolescents. *SUCHT* 2015;61(6):377-87. doi: 10.1024/0939-5911.a000397
41. Miller WR, Rollnick S. Motivational Interviewing: Helping People Change. 3rd ed. New York: The Guilford Press 2013.
42. Ajzen I. The Theory of Planned Behavior. *Organizational Behavior and Human Decision Processes* 1991;50(2):179-211.
43. Bandura A. Social foundations of thought and action: A social cognitive theory. NJ: Prentice-Hall: Englewood Cliffs 1986.
44. Voogt CV, Poelen EA, Kleinjan M, et al. The effectiveness of the 'what do you drink' web-based brief alcohol intervention in reducing heavy drinking among students: a two-arm parallel group randomized controlled trial. *Alcohol Alcohol* 2013;48(3):312-21. doi: 10.1093/alcalc/ags133 [published Online First: 2013/01/11]
45. Wood MD, Fairlie AM, Fernandez AC, et al. Brief Motivational and Parent Interventions for College Students: A Randomized Factorial Study. *J Consult Clin Psych* 2010;78(3):349-61. doi: Doi 10.1037/A0019166
46. Carey KB, Scott-Sheldon LAJ, Elliott JC, et al. Computer-delivered interventions to reduce college student drinking: a meta-analysis. *Addiction (Abingdon, England)* 2009;104(11):1807-19. doi: 10.1111/j.1360-0443.2009.02691.x [published Online First: 2009/09/10]
47. Moreira MT, Smith LA, Foxcroft D. Social norms interventions to reduce alcohol misuse in university or college students. *The Cochrane database of systematic reviews* 2009(3):Cd006748. doi: 10.1002/14651858.CD006748.pub2 [published Online First: 2009/07/10]
48. Doumas DM, Hannah E. Preventing high-risk drinking in youth in the workplace: a web-based normative feedback program. *Journal of substance abuse treatment* 2008;34(3):263-71. doi: 10.1016/j.jsat.2007.04.006 [published Online First: 2007/06/30]
49. DONALDSON SI, SUSSMAN S, MacKINNON DP, et al. Drug Abuse Prevention Programming:Do We Know What Content Works? *American Behavioral Scientist* 1996;39(7):868-83. doi: 10.1177/0002764296039007008
50. Voogt CV, Poelen EAP, Kleinjan M, et al. Targeting young drinkers online: the effectiveness of a web-based brief alcohol intervention in reducing heavy drinking among college students: study protocol of a two-arm parallel group randomized controlled trial. *BMC Public Health* 2011;11(1):231. doi: 10.1186/1471-2458-11-231
51. Frost H, Campbell P, Maxwell M, et al. Effectiveness of Motivational Interviewing on adult behaviour change in health and social care settings: A systematic review of reviews. *PloS one* 2018;13(10):e0204890-e90. doi: 10.1371/journal.pone.0204890

For peer review only - http://bmjopen.bmj.com/site/about/guidelines.xhtml

52. Kohler S, Hofmann A. Can motivational interviewing in emergency care reduce alcohol consumption in young people? A systematic review and meta-analysis. *Alcohol Alcohol* 2015;50(2):107-17. doi: 10.1093/alcalc/agu098 [published Online First: 2015/01/08]
53. Li L, Zhu S, Tse N, et al. Effectiveness of motivational interviewing to reduce illicit drug use in adolescents: a systematic review and meta-analysis. *Addiction* 2016;111(5):795-805. doi: 10.1111/add.13285 [published Online First: 2015/12/22]
54. Tanner-Smith EE, Lipsey MW. Brief alcohol interventions for adolescents and young adults: a systematic review and meta-analysis. *Journal of substance abuse treatment* 2015;51:1-18. doi: 10.1016/j.jsat.2014.09.001 [published Online First: 2014/10/11]
55. Bahner J, Stenqvist K. Motivational Interviewing as Evidence-Based Practice? An Example from Sexual Risk Reduction Interventions Targeting Adolescents and Young Adults. *Sexuality Research and Social Policy* 2019 doi: 10.1007/s13178-019-00388-y
56. Gates P, Norberg M, Copeland J, et al. Randomized controlled trial of a novel cannabis use intervention delivered by telephone. *Addiction (Abingdon, England)* 2012;107 doi: 10.1111/j.1360-0443.2012.03953.x
57. Berman AH, Beckman M, Lindqvist H. Motivational Interviewing interventions. In: Hagger MS, Cameron LD, Hamilton K, et al., eds. Handbook of Behavior Change. New York, NY: Cambridge University Press. In press, 2019.
58. Bewick BM, Trusler K, Barkham M, et al. The effectiveness of web-based interventions designed to decrease alcohol consumption — A systematic review. *Preventive Medicine* 2008;47(1):17-26. doi: https://doi.org/10.1016/j.ypmed.2008.01.005
59. White A, Kavanagh D, Stallman H, et al. Online Alcohol Interventions: A Systematic Review. *J Med Internet Res* 2010;12(5):e62. doi: 10.2196/jmir.1479
60. Toumbourou JW, Stockwell T, Neighbors C, et al. Interventions to reduce harm associated with adolescent substance use. *Lancet (London, England)* 2007;369(9570):1391-401. doi: 10.1016/S0140-6736(07)60369-9
61. Natarajan M, Kaner E. Brief alcohol interventions in young people: evidence and applicability. I E. Gilvarry & P. McArdle (redaktörer), Alcohol, drugs and young people: clinical approaches. Newcastle upon Tyne: Mac Keith Press 2007.
62. Sinadinovic K, Wennberg P, Berman AH. Internet-based screening and brief intervention for illicit drug users: a randomized controlled trial with 12-month follow-up. *J Stud Alcohol Drugs* 2014;75(2):313-8. [published Online First: 2014/03/22]
63. Tait RJ, Spijkerman R, Riper H. Internet and computer based interventions for cannabis use: A meta-analysis. *Drug and Alcohol Dependence* 2013;133(2):295-304. doi: https://doi.org/10.1016/j.drugalcdep.2013.05.012
64. Cavanagh K, Millings A. (Inter)personal Computing: The Role of the Therapeutic Relationship in E-mental Health. *Journal of Contemporary Psychotherapy* 2013;43(4):197-206. doi: 10.1007/s10879-013-9242-z
65. Webb TL, Joseph J, Yardley L, et al. Using the internet to promote health behavior change: a systematic review and meta-analysis of the impact of theoretical basis, use of behavior change techniques, and mode of delivery on efficacy. *J Med Internet Res* 2010;12(1):e4. doi: 10.2196/jmir.1376 [published Online First: 2010/02/19]
66. Lee CM, Neighbors C, Kilmer JR, et al. A brief, web-based personalized feedback selective intervention for college student marijuana use: a randomized clinical trial. *Psychol Addict Behav* 2010;24(2):265-73. doi: 10.1037/a0018859 [published Online First: 2010/06/23]
67. Rooke S, Copeland J, Norberg M, et al. Effectiveness of a self-guided web-based cannabis treatment program: randomized controlled trial. *Journal of medical Internet research* 2013;15(2):e26-e26. doi: 10.2196/jmir.2256

For peer review only - http://bmjopen.bmj.com/site/about/guidelines.xhtml

68. Tonnesen H, H S, Pedersen B. Review: Web-based brief interventions for young adolescent alcohol and drug abusers - a systematic review2013.
69. Chan A-W, Tetzlaff JM, Altman DG, et al. SPIRIT 2013 statement: defining standard protocol items for clinical trials. *Ann Intern Med* 2013;158(3):200-07. doi: 10.7326/0003-4819-158-3-201302050-00583
70. Cohen J. A power primer. *Psychological bulletin* 1992;112(1):155-9. [published Online First: 1992/07/01]
71. Riper H, Hoogendoorn A, Cuijpers P, et al. Effectiveness and treatment moderators of internet interventions for adult problem drinking: An individual patient data meta-analysis of 19 randomised controlled trials. *PLOS Medicine* 2018;15(12):e1002714. doi: 10.1371/journal.pmed.1002714
72. Knight JR, Shrier LA, Bravender TD, et al. A new brief screen for adolescent substance abuse. *Arch Pediatr Adolesc Med* 1999;153(6):591-6. [published Online First: 1999/06/05]
73. Levy S, Sherritt L, Harris SK, et al. Test-retest reliability of adolescents' self-report of substance use. *Alcohol Clin Exp Res* 2004;28(8):1236-41. [published Online First: 2004/08/20]
74. Knight JR, Sherritt L, Harris SK, et al. Validity of brief alcohol screening tests among adolescents: a comparison of the AUDIT, POSIT, CAGE, and CRAFFT. *Alcohol Clin Exp Res* 2003;27(1):67-73. doi: 10.1097/01.ALC.0000046598.59317.3A [published Online First: 2003/01/25]
75. Kallmen H, Berman AH, Jayaram-Lindstrom N, et al. Psychometric Properties of the AUDIT, AUDIT-C, CRAFFT and ASSIST-Y among Swedish Adolescents. *European addiction research* 2019;25(2):68-77. doi: 10.1159/000496741 [published Online First: 2019/02/07]
76. Saunders JB, Aasland OG, Babor TF, et al. Development of the Alcohol Use Disorders Identification Test (AUDIT): WHO Collaborative Project on Early Detection of Persons with Harmful Alcohol Consumption--II. *Addiction* 1993;88(6):791-804.
77. Rumpf HJ, Wohlert T, Freyer-Adam J, et al. Screening questionnaires for problem drinking in adolescents: performance of AUDIT, AUDIT-C, CRAFFT and POSIT. *European addiction research* 2013;19(3):121-7. doi: 10.1159/000342331 [published Online First: 2012/11/28]
78. Collins RL, Parks GA, Marlatt GA. Social determinants of alcohol consumption: the effects of social interaction and model status on the self-administration of alcohol. *J Consult Clin Psychol* 1985;53(2):189-200. [published Online First: 1985/04/01]
79. Kivlahan DR, Marlatt GA, Fromme K, et al. Secondary prevention with college drinkers: Evaluation of an alcohol skills training program. *J Consult Clin Psych* 1990;58(6):805-10. doi: 10.1037/0022-006X.58.6.805
80. Higgins-Biddle JC, Babor TF. A review of the Alcohol Use Disorders Identification Test (AUDIT), AUDIT-C, and USAUDIT for screening in the United States: Past issues and future directions. *The American journal of drug and alcohol abuse* 2018;44(6):578-86. doi: 10.1080/00952990.2018.1456545 [published Online First: 2018/05/03]
81. Berman AH, Bergman H, Palmstierna T, et al. Evaluation of the Drug Use Disorders Identification Test (DUDIT) in criminal justice and detoxification settings and in a Swedish population sample. *European addiction research* 2005;11(1):22-31. doi: 81413 [pii] 10.1159/000081413 [published Online First: 2004/12/21]
82. Hildebrand M. The psychometric properties of the Drug Use Disorders Identification Test (DUDIT): A review of recent research. *Journal of substance abuse treatment* 2015 a;53:52-59.
83. Sinadinovic K, Wennberg P, Berman AH. Population screening of risky alcohol and drug use via Internet and Interactive Voice Response (IVR): A feasibility and psychometric study in a random sample. *Drug and Alcohol Dependence* 2011;114(1):55-60. doi: https://doi.org/10.1016/j.drugalcdep.2010.09.004

For peer review only - http://bmjopen.bmj.com/site/about/guidelines.xhtml

84. Topp CW, Ostergaard SD, Sondergaard S, et al. The WHO-5 Well-Being Index: a systematic review of the literature. *Psychotherapy and psychosomatics* 2015;84(3):167-76. doi: 10.1159/000376585 [published Online First: 2015/04/04]
85. Love J, Andersson L, Moore CD, et al. Psychometric analysis of the Swedish translation of the WHO well-being index. *Quality of life research : an international journal of quality of life aspects of treatment, care and rehabilitation* 2014;23(1):293-7. doi: 10.1007/s11136-013-0447-0 [published Online First: 2013/06/08]
86. Strandberg AK, Skoglund C, Gripenberg J, et al. Alcohol and illicit drug consumption and the association with risky sexual behavior among Swedish youths visiting youth health clinics. *Nordic Studies on Alcohol and Drugs* Accepted
87. Brown BB, Clasen DR, Eicher SA. Perceptions of peer pressure, peer conformity dispositions, and self-reported behavior among adolescents. *Developmental Psychology* 1986;22(4):521-30. doi: 10.1037/0012-1649.22.4.521
88. Frost RO, Marten P, Lahart C, et al. The dimensions of perfectionism. *Cognitive Therapy and Research* 1990;14(5):449-68. doi: 10.1007/BF01172967
89. Rozental A, Shafran R, Wade T, et al. A randomized controlled trial of Internet-Based Cognitive Behavior Therapy for perfectionism including an investigation of outcome predictors. *Behaviour research and therapy* 2017;95:79-86. doi: 10.1016/j.brat.2017.05.015 [published Online First: 2017/06/07]
90. Stattin H, Kerr M. Parental monitoring: a reinterpretation. *Child development* 2000;71(4):1072-85. [published Online First: 2000/10/04]
91. Salari R, Thorell LB. Parental monitoring in late adolescence: Relations to ADHD symptoms and longitudinal predictors. *Journal of Adolescence* 2015;40:24.
92. Kerr M, Stattin H. What parents know, how they know it, and several forms of adolescent adjustment: further support for a reinterpretation of monitoring. *Dev Psychol* 2000;36(3):366-80. [published Online First: 2000/06/01]
93. Marlatt GA, Baer JS, Kivlahan DR, et al. Screening and brief intervention for high-risk college student drinkers: Results from a 2-year follow-up assessment. *J Consult Clin Psych* 1998;66(4):604-15.
94. Widmark E. Die theoretischen Grundlagen und die praktische Verwendbarkeit der gerichtlich-medizinischen Alkoholbestimmung. Berlin Wien: Urban und Schwarzenberg 1932.
95. Babor T, Higgins-Biddle J. Brief Intervention For Hazardous and Harmful Drinking: A Manual for use in Primary Care: World health Organization 2001.
96. Young RM, Hasking PA, Oei TP, et al. Validation of the Drinking Refusal Self-Efficacy Questionnaire--Revised in an Adolescent Sample (DRSEQ-RA). *Addictive behaviors* 2007;32(4):862-8. doi: 10.1016/j.addbeh.2006.07.001 [published Online First: 2006/08/22]
97. Gibbons RD, Hedeker D, DuToit S. Advances in analysis of longitudinal data. *Annu Rev Clin Psychol* 2010;6:79-107. doi: 10.1146/annurev.clinpsy.032408.153550
98. Richards D, Richardson T. Computer-based psychological treatments for depression: a systematic review and meta-analysis. *Clin Psychol Rev* 2012;32(4):329-42. doi: 10.1016/j.cpr.2012.02.004 [published Online First: 2012/02/28]
99. Cole HA, Prassel HB, Carlson CR. A Meta-Analysis of Computer-Delivered Drinking Interventions for College Students: A Comprehensive Review of Studies From 2010 to 2016. *J Stud Alcohol Drugs* 2018;79(5):686-96. [published Online First: 2018/11/14]
100. Berman AH, Gajecki M, Sinadinovic K, et al. Mobile Interventions Targeting Risky Drinking Among University Students: A Review. *Curr Addict Rep* 2016;3:166-74. doi: 10.1007/s40429-016-0099-6 [published Online First: 2016/04/05]

For peer review only - http://bmjopen.bmj.com/site/about/guidelines.xhtml

FIGURE LEGENDS

Figure 1 Study design

For peer review only

For peer review only - http://bmjopen.bmj.com/site/about/guidelines.xhtml

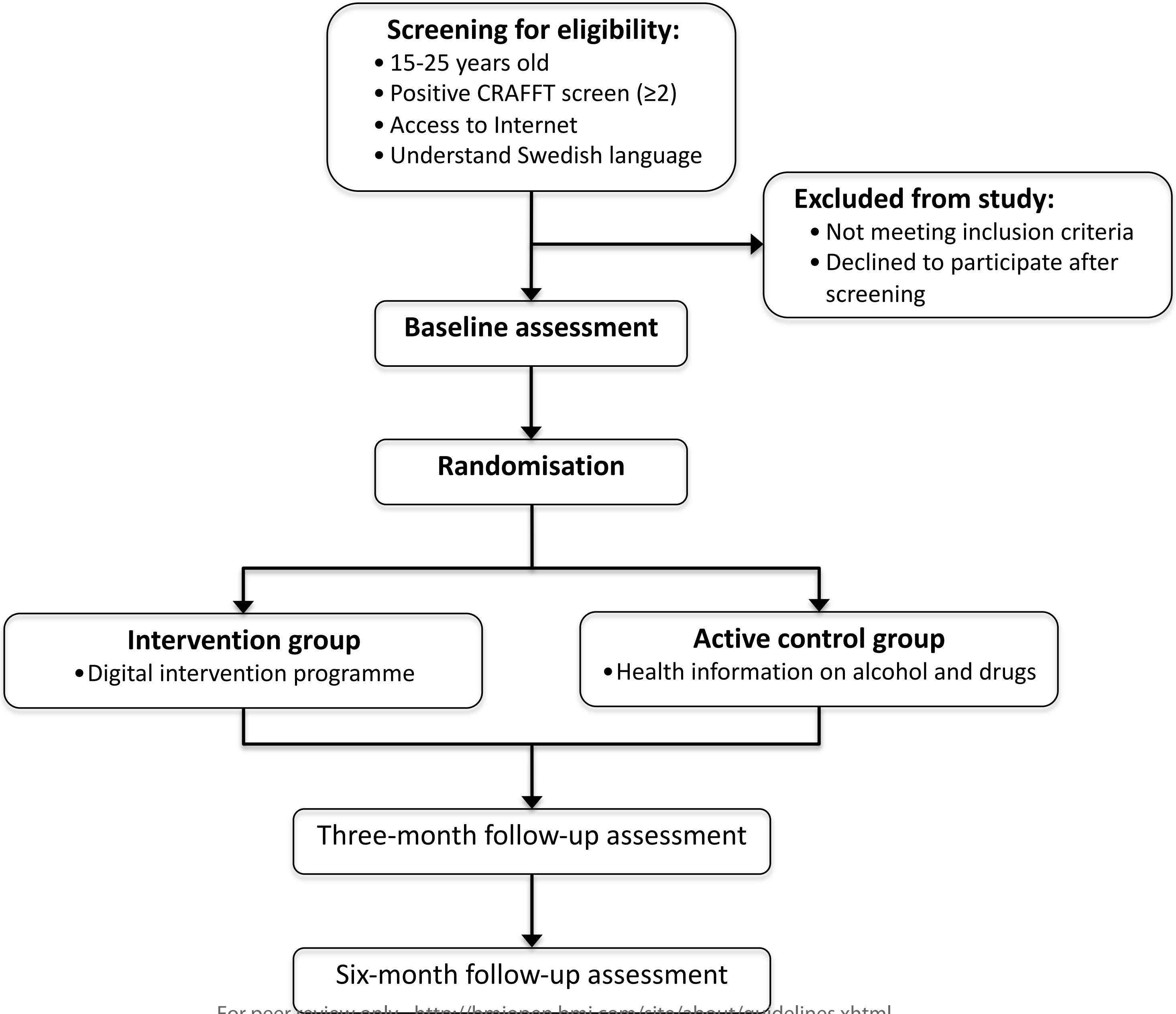
Screening for eligibility:
• 15-25 years old
• Positive CRAFFT screen (≥2)
• Access to Internet
• Understand Swedish language
Excluded from study:
• Not meeting inclusion criteria
• Declined to participate after screening
Baseline assessment
Randomisation
Intervention group
• Digital intervention programme
Active control group
• Health information on alcohol and drugs
Three-month follow-up assessment
Six-month follow-up assessment

BMJ Open

Effects of an automated digital brief prevention intervention targeting adolescents and young adults with risky alcohol and other substance use: Study protocol for a randomised controlled trial

Journal:	*BMJ Open*
Manuscript ID	bmjopen-2019-034894.R3
Article Type:	Protocol
Date Submitted by the Author:	14-Apr-2020
Complete List of Authors:	Kvillemo, Pia; Karolinska Institutet, STAD, Centre for Psychiatry Research, Department of Clinical Neuroscience, Karolinska Institutet & Stockholm Health Care Services, Region Stockholm, Norra Stationsgatan 69, SE-113 64<br>Strandberg, Anna; Karolinska Institutet, STAD, Centre for Psychiatry Research, Department of Clinical Neuroscience, Karolinska Institutet & Stockholm Health Care Services, Region Stockholm<br>Gripenberg, Johanna; Karolinska Institutet, STAD, Centre for Psychiatry Research, Department of Clinical Neuroscience, Karolinska Institutet & Stockholm Health Care Services, Region Stockholm<br>Berman, Anne; Karolinska Institutet, Centre for Psychiatry Research, Department of Clinical Neuroscience, Karolinska Institutet & Stockholm Health Care Services, Region Stockholm, Department of Psychology, Uppsala University, Uppsala<br>Skoglund, Charlotte; Karolinska Institutet, STAD, Centre for Psychiatry Research, Department of Clinical Neuroscience, Karolinska Institutet & Stockholm Health Care Services, Region Stockholm, Department of Pharmaceutical Biosciences, Uppsala University, Uppsala<br>Elgán, Tobias; Karolinska Institutet, STAD, Centre for Psychiatry Research, Department of Clinical Neuroscience, Karolinska Institutet & Stockholm Health Care Services, Region Stockholm
**Primary Subject Heading**:	Public health
Secondary Subject Heading:	Addiction, Medical education and training, Mental health, Sexual health
Keywords:	PSYCHIATRY, Substance misuse < PSYCHIATRY, Child & adolescent psychiatry < PSYCHIATRY, PREVENTIVE MEDICINE

SCHOLARONE™
Manuscripts

I, the Submitting Author has the right to grant and does grant on behalf of all authors of the Work (as defined in the below author licence), an exclusive licence and/or a non-exclusive licence for contributions from authors who are: i) UK Crown employees; ii) where BMJ has agreed a CC-BY licence shall apply, and/or iii) in accordance with the terms applicable for US Federal Government officers or employees acting as part of their official duties; on a worldwide, perpetual, irrevocable, royalty-free basis to BMJ Publishing Group Ltd ("BMJ") its licensees and where the relevant Journal is co-owned by BMJ to the co-owners of the Journal, to publish the Work in this journal and any other BMJ products and to exploit all rights, as set out in our licence.

The Submitting Author accepts and understands that any supply made under these terms is made by BMJ to the Submitting Author unless you are acting as an employee on behalf of your employer or a postgraduate student of an affiliated institution which is paying any applicable article publishing charge ("APC") for Open Access articles. Where the Submitting Author wishes to make the Work available on an Open Access basis (and intends to pay the relevant APC), the terms of reuse of such Open Access shall be governed by a Creative Commons licence – details of these licences and which Creative Commons licence will apply to this Work are set out in our licence referred to above.

Other than as permitted in any relevant BMJ Author's Self Archiving Policies, I confirm this Work has not been accepted for publication elsewhere, is not being considered for publication elsewhere and does not duplicate material already published. I confirm all authors consent to publication of this Work and authorise the granting of this licence.

For peer review only

Effects of an automated digital brief prevention intervention targeting adolescents and young adults with risky alcohol and other substance use: Study protocol for a randomised controlled trial

Authors

Pia Kvillemo (corresponding author)
STAD, Centre for Psychiatry Research,
Department of Clinical Neuroscience, Karolinska Institutet & Stockholm Health Care Services, Region Stockholm
Norra Stationsgatan 69, SE-113 64 Stockholm, Sweden
+46 (0)70 673 48 64
pia.kvillemo@ki.se

Anna K Strandberg
STAD, Centre for Psychiatry Research,
Department of Clinical Neuroscience, Karolinska Institutet, & Stockholm Health Care Services, Region Stockholm
Stockholm, Sweden

Johanna Gripenberg
STAD, Centre for Psychiatry Research,
Department of Clinical Neuroscience, Karolinska Institutet, & Stockholm Health Care Services, Region Stockholm
Stockholm, Sweden

Anne H Berman
Centre for Psychiatry Research,
Department of Clinical Neuroscience, Karolinska Institutet, & Stockholm Health Care Services, Region Stockholm
Stockholm, Sweden
Department of Psychology, Uppsala University, Uppsala, Sweden

Charlotte Skoglund
STAD, Centre for Psychiatry Research,
Department of Clinical Neuroscience, Karolinska Institutet, & Stockholm Health Care Services, Region Stockholm
Stockholm, Sweden
Department of Pharmaceutical Biosciences, Uppsala University, Uppsala, Sweden

Tobias H Elgán
STAD, Centre for Psychiatry Research,
Department of Clinical Neuroscience, Karolinska Institutet, & Stockholm Health Care Services, Region Stockholm
Stockholm, Sweden

Keywords
Motivational Interviewing, Decisional balance, Substance use, Web-based intervention, Normative feedback.

Wordcount
4655

For peer review only - http://bmjopen.bmj.com/site/about/guidelines.xhtml

Effects of an automated digital brief prevention intervention targeting adolescents and young adults with risky alcohol and other substance use: Study protocol for a randomised controlled trial

ABSTRACT

Introduction

Adolescence and young adulthood is a period in life when individuals may be especially vulnerable to harmful substance use. Several critical developmental processes are occurring in the brain, and substance use poses both short- and long-term risks with regard to mental health and social development. From a public health perspective, it is important to prevent or delay substance use to reduce individual risk and societal costs. Given the scarcity of effective interventions targeting substance use among adolescents and young adults, cost-effective and easily disseminated interventions are warranted. The current study will test the effectiveness of a fully automated digital brief intervention aimed at reducing alcohol and other substance use in adolescents and young adults aged 15-25 years.

Methods and analysis

A two-arm double-blind randomised controlled trial (RCT) design is applied to assess the effectiveness of the intervention. Baseline assessment, as well as three- and six-month follow-up, will be carried out. The aim is to include 800 participants with risky substance use based on the screening tool CRAFFT. Recruitment, informed consent, randomisation, intervention, and follow-up will be implemented online. The primary outcome is reduction in alcohol use, measured by AUDIT-C total score. Secondary outcomes concern binge drinking, frequency of alcohol consumption, amount of alcohol consumed a typical day when alcohol is consumed, average daily drinks per typical week, other substance use, mental health, sexual risk behaviours, and perceived peer pressure. Moreover, the study involves analyses of potential moderators including perfectionism, openness to parents, help-seeking, and background variables.

For peer review only - http://bmjopen.bmj.com/site/about/guidelines.xhtml

For peer review only

Ethics and dissemination

The study was approved by the Swedish Ethical Review Authority (no. 2019-03249). The trial is expected to expand the knowledge on digital preventive interventions for substance using adolescents and young adults. Results will be disseminated in research journals, at conferences and via the media.

Registration

24/09/2019, ISRCTN91048246.

Key words

Motivational Interviewing, Decisional balance, Substance use, Web-based intervention, Normative feedback.

ARTICLE SUMMARY

Strengths and limitations of this study

- A double-blind randomised controlled trial (RCT) is considered to be the most robust experimental design, enabling causal inferences, controlling for selection bias and participant allocation bias.
- An active control condition, blinded to the study condition, controls for expectation, detection and performance bias.
- Analysis of several potential moderators, i.e., perfectionism, openness to parents, help-seeking and background variables will contribute to the understanding of possible effects.
- A convenience sample may reduce generalizability due to possible selection bias at recruitment.

For peer review only

INTRODUCTION

Substance use is a major public health concern causing individual suffering as well as societal costs.[1-3] Substance use in childhood and younger ages is particularly harmful since the brain is undergoing critical development during this time in life, which makes it more vulnerable to addictive substances.[4 5] In adolescence, some individuals are especially prone to various risk behaviours, increasing the risk of e.g., substance use.[6] Additionally, having substance using peers is a prominent risk factor for one's own substance use at this age, since peers are increasingly important in teenagers' social life.[7] Thus, the initiation of substance use often occurs during this period.[6] After initiation, the use of substances frequently increases during adolescence and young adulthood, posing a number of risks for the individual.[2 8 9] Early onset of substance use implies a risk of severe adverse effects on psychological and physiological development.[2 8 9] Hazardous use of alcohol, cannabis and other substances can lead to chronic problematic consumption patterns and addiction that can significantly influence the developmental trajectory during the transition from childhood to adulthood.[10] More acute consequences include, e.g., mental or psychiatric problems, problems of academic adjustment,[11] accidents,[12] and problems with the police or legal authorities.[13 14] Risk behaviours, such as substance use, binge drinking, intoxication, and sexual risk behaviour often co-occur, and the use of illicit substances, is e.g., often accompanied by alcohol consumption.[1 2 11 15-19] Poly-substance use, i.e., simultaneous use of different substances, may imply an increased risk of various negative outcomes, e.g., mental health problems, shown to increase in magnitude and over time, with the number of substances used.[20] The well-established association between substance use and mental health problems is bidirectional, sometimes manifested in self-medication for mental health problems, and sometimes as an increase in such problems due to substance use.[21] While most of prior research shows that low socio-economic standard is a risk factor for substance use and related problems,[22 23] the current study is informed by recent research on mechanisms involved in adolescence alcohol and other substance use among upper secondary school students from affluent areas,

For peer review only - http://bmjopen.bmj.com/site/about/guidelines.xhtml

showing associations between internalizing symptoms and substance use,[24 25] as well as associations between achievement pressures (particularly excessive perfectionistic strivings), and isolation from parents (particularly low perceived closeness to mothers).[24 26] The latter finding is in line with previous research showing that a positive parent–child relationship is a protective factor related to lower likelihood of adolescence substance use.[27 28]

Although alcohol use has declined among adolescents during recent years, some individuals drink more and binge drinking is still highly prevalent among young adults, where the largest proportion of alcohol risk consumers is still found.[29 30] Moreover, a high prevalence of substance use and poly-substance use among young people in Europe was recognized in the EU Drug Strategy 2005–2012, which calls for action. [31] Also, established cannabis users among Swedish upper secondary school students tend to use cannabis more often than before, according to recent survey data.[32] In light of these circumstances, it is of utmost importance to target and tailor attractive preventive interventions for adolescents and young adults. One way to reach large groups of young people is to disseminate digital interventions online (i.e., via the Internet). In Sweden, 98 percent of the population has access to the Internet at home and virtually 100 percent of 16-25 year-olds use the Internet in mobile phones, tablets or computers.[33] Digital interventions have several advantages over traditional ones. They may reduce the stigma around risk behaviours, they are accessible at any time and place, and can be cost-effective because minimal staff resources are used for implementation, especially if they are fully automated.[34 35] Digital interventions may thus be particularly suitable for adolescents and young adults.[36] To date, digital prevention programmes targeting alcohol and other substance use among adolescents and young adults that meet scientific evaluation criteria are scarce. An early example is eScreen.se, offering screening for alcohol and drug use with personalized feedback.[37] A more recent example is a fully automated brief motivational intervention for substance-using 16-18 year-olds, tested in an RCT in four European countries,[38-40] where our research group was one of the partners. The intervention, named WISEteens, relied on Motivational Interviewing (MI)[41] and models of Social Influence.[42 43] The goal of the MI approach was to enhance motivation to change by exploring and resolving ambivalence about substance-related behaviours. An important element in the intervention was personalized feedback about one's substance use behaviours in relation to normative comparisons, as personalized feedback is perceived as more relevant for changing behavior than more general information.[44] The normative feedback included information about how a specific reference group actually consumed substances, in order to correct participants' "inflated perception",[45] which has proven effective for reduction of alcohol consumption in young adults in previous meta-analyses.[46 47] For adolescents and young adults, a normative feedback approach may be particularly appealing, assuming they are curious about how their substance consumption compares to their peers.[48]

For peer review only - http://bmjopen.bmj.com/site/about/guidelines.xhtml

Likewise, an important element of the intervention was a focus on substance-related social norms and training on how to avoid social high-risk situations and how to resist peer pressure; i.e., raising refusal self-efficacy.[49] In line with the social influence hypothesis stating that a prominent risk factor for substance use in adolescence is the influence of substance using peers,[7] targeting peer pressure in brief digital interventions has been an important element for reducing alcohol use among adolescents.[50] WISEteens demonstrated significant between-group effects for alcohol use, indicating that a targeted brief motivational intervention in a fully automated digital format can be effective to reduce drinking and lower barriers for accessing substance use service in hazardous drinking adolescents.[38]

Digital interventions, often delivered online, generally offer adaptations of evidence based face-to-face interventions, e.g., MI. The effects of MI on several health-related behaviours have been evaluated in a number of other studies, especially among adults. [51] Among adolescents, systematic reviews and meta-analyses on MI for various health-related behaviours show mixed results. A review of six studies of MI for reducing alcohol consumption in the emergency room setting suggested that MI was at least as effective as other brief interventions in the same setting.[52] Another review of 24 studies of different brief interventions for reducing alcohol consumption and alcohol-related problems among adolescents showed a significant effect that persisted up to one year compared to control interventions, with greater effects for MI. Effects were consistent over diverse settings and particularly effective components included decisional balance (juxtaposition of pros and cons of change)[53] and goal-setting[54]. Regarding MI for illicit drug use among adolescents, a review of 10 studies showed no effects on drug use behaviors; however, changes in attitudes towards drug use were found, which could be translated into intentions to change behaviors [53]. A scoping review concerning MI for reducing sexual risk behaviors among adolescents identified 29 unique studies with varying designs and conceptualizations of MI and specific risk behaviours, making it difficult to generalize regarding outcomes but indicating the need of more research.[55] MI has most commonly been delivered as an individual face-to-face intervention[51], but has also been provided in other forms, e.g., via telephone or digitally, with various results on e.g., substance use behaviours.[56 57] Digital programmes based on MI have demonstrated effect in the form of reduced alcohol consumption among young adults.[58 59] Additionally, a combination of screening with a short intervention has shown similar effects on alcohol consumption among adolescents.[60] Brief motivational interventions are empirically supported individual level interventions for reducing alcohol consumption.[45 61] Such interventions have in the last decades also been digitally provided.[62 63] Digital interventions can be provided with or without human guidance, i.e., more or less automated.[64] Previous research has demonstrated that even fully automated interventions can reduce alcohol-related problems for young people with risky alcohol consumption up to 12 months after implementation,[59 65] and indicated potential

For peer review only - http://bmjopen.bmj.com/site/about/guidelines.xhtml

effect on cannabis use in certain groups.[66 67] Although various digital substance use prevention interventions have been developed and tested with promising results,[38 68] studies on the effects of fully automated digitally delivered and MI-based substance use prevention programmes for adolescents and young adults are still few[68] and more well-designed studies are warranted in order to obtain evidence for potential effects.

Aim

The study described in this protocol builds on results previously obtained in a European study of a digital brief MI-based substance use prevention intervention (WISEteens), delivered online to adolescents in a fully automated form.[38] In this study, the effectiveness of a modified version of this intervention, with new graphics and population based feedback-generating data on alcohol consumption covering all included age groups, will be tested across a range of outcome measures among 15-25 year-olds with hazardous alcohol or other substance use. The primary aim is reduction in frequency and quantity of substance use.

Rationale and hypothesis

The current study will add to existing evidence regarding the effectiveness of digital interventions aimed at reducing alcohol and other substance use among young people. We hypothesize that participants in the intervention group will report a significant reduction in substance use (primarily alcohol use), compared to an active control group receiving health information about various substances. A number of potential moderators will be assessed to confirm or contradict previous research on factors influencing the development of substance use patterns over time, and effects of prevention interventions in different target groups.

METHODS AND ANALYSIS

In order to evaluate the effectiveness of the digital screening and brief motivational intervention, a double-blind two-arm RCT study design is planned with baseline assessment at study entry and follow-up assessment at three and six-months across a number of outcome variables. Figure 1 displays the trial design. We will also explore and test for moderator effects. To outline and report the current study we used SPIRIT reporting guidelines.[69]

For peer review only - http://bmjopen.bmj.com/site/about/guidelines.xhtml

Recruitment

The target group for the current study is adolescents and young adults aged 15-25 years with self-reported substance use, a capacity to understand the Swedish language, and access to the Internet. Based on a power calculation to detect a small effect size (Cohen's d=0.2),[70] the aim is to include 800 participants.[71] The participants will be recruited during January-October 2020 by offline marketing at youth health clinics, other health care units for youth, and at upper secondary schools in Stockholm county, as well as by social networks, using online banner advertisements. This will result in a convenience sample from the general Swedish population.[44] Results from follow-up measures are to be completely received in 2021. If sufficient number of participants have not been recruited according to plan, we will extend the recruitment period and intensify the advertising by increasing visibility on social platforms. To enhance participation and follow-up rates, an incentive in the form of a movie ticket, with a value up to 15 Euros, for each completed follow-up assessment will be provided, i.e., up to 3 tickets. Up to three reminders will be sent out if participants do not respond to follow-up assessments.

Screening and informed consent

Potential participants will be guided to an online landing page with screening by an adapted version of the screening tool for use of alcohol and other substances, CRAFFT (Car, Relax, Alone, Forget, Friends, Trouble).[72] This six-item screening tool has demonstrated criterion validity and appropriateness for identifying substance-related problems among adolescents.[73-75] The primary eligibility criterion for participating in the study will be a score of 2 or more on the CRAFFT, since this value has shown satisfactory sensitivity for identifying substance use problems.[72] Those who score 2 or higher will be offered participation in the study and given information on confidentiality, voluntariness of participation, and human subject protections.[44] They will also be provided with contact information on suitable counselling service providers. Those who agree to participate will be asked to give digital informed consent.

Measures

All assessment will occur online. The selection of measures and associated instruments is based on the aim of the study and the theoretical base concerning factors influencing substance use. Based on results

For peer review only - http://bmjopen.bmj.com/site/about/guidelines.xhtml

from the previous European WISEteens study,[38] showing significant effects on alcohol consumption but not on other substance use, the primary outcome is alcohol use, measured at three- and six-month follow-ups. Secondary outcomes include binge drinking, frequency of alcohol consumption, amount of alcohol consumed a typical day when alcohol is consumed, average daily drinks per typical week, at three- and six-months follow-ups, other substance use, mental health, sexual risk behaviours, and perceived peer pressure at three and six-month follow-ups. The moderating variables include perfectionism, openness to parents, help-seeking due to mental health problems, and socio-demographic and personal characteristics, (i.e. sex, age, residence, occupation, school performance (for students), and parents' education).

Alcohol use, including the primary outcome, will be measured using two instruments. One of them is the short version of the Alcohol Use Disorders Identification Test (AUDIT-C),[76] measuring frequency and amount of consumed alcohol and frequency of binge drinking, providing a widely used and valid index sum score for problematic alcohol use among adolescents.[75 77] The other is the Daily Drinking Questionnaire (DDQ),[78] measuring a variety of parameters of alcohol use in a typical week. Previous research has demonstrated that the DDQ is highly correlated with other measures of self-reported alcohol consumption.[79] The primary outcome will be measured by AUDIT-C total score. The two measures of alcohol use complement one another in that the AUDIT-C primarily offers an indication of hazardous or harmful use,[80] whereas the DDQ quantity measure (average daily drinks per typical week) will indicate the level of alcohol consumption in grams per week. Other substance use will be measured by a short version of the Drug Use Disorder Identification Test (DUDIT),[81] including the first four items (DUDIT-C) which assess frequency of consumption of drugs other than alcohol, frequency of different types of drugs other than alcohol used at the same occasion (1 = never to 5 = four or more times a week), and number of occasions when drugs other than alcohol are consumed on a typical day of drug use (1 = zero to 5 = seven or more). The DUDIT has been found to be effective in screening for drug-related problems in clinically selected groups,[82] and both the DUDIT and the DUDIT-C have proven useful in the context of public health surveys in Sweden.[81 83] Mental health will be measured by The WHO-5 Well-Being Index, which has demonstrated validity both as a screening tool for depression and as an outcome measure in clinical trials.[84] This scale has been successfully applied across a wide range of study fields, translated into more than 30 languages, including Swedish,[85] and found psychometrically sound. Changes in sexual risk behaviours will be measured by multiple choice questions on sexual behaviour under the influence of alcohol and/or other substances and unprotected sex, previously used in a survey among Swedish visitors at youth health clinics in Stockholm county.[86] Changes in perceived peer pressure will be measured by

For peer review only - http://bmjopen.bmj.com/site/about/guidelines.xhtml

two items retrieved and adapted from the Peer pressure inventory.[87] Perfectionism will be measured by two subscales of the Frost Multidimensional Perfectionism Scale (FMPS).[88 89] Openness to parents will be measured among participants who are up to 20 years old with questions about disclosure from Stattin & Kerr,[90-92] slightly modified into statements to be compatible with the digital online format. Help-seeking due to mental health problems will be measured by dichotomous questions regarding if, and to what healthcare provider, the participant has turned for help. Additionally, background data will be asked for using multiple choice questions: sex (man, women, other), age (15, 16…25), residence (name of municipality), occupation (secondary school, upper secondary school, university, not student, working, practicing, unemployed, other), school performance (merit value for students), and parents' education (secondary school, upper secondary school, university, don't know).

Randomisation

The study is double-blind, thus neither the participant nor the researchers will know which participant is allocated to the intervention or to the active control condition. After baseline assessment, the participants will be automatically randomised to one of the two study groups by a computer programme using an unrestricted randomisation protocol. Participants will then be informed about the name of their programme and given access immediately.

The intervention

Participants allocated to the intervention group will initially be asked to give additional information on alcohol and other substance use and to state their body weight, in order to generate personalized feedback on substance use behaviour and to estimate alcohol blood concentration on a typical drinking occasion.

Description of the intervention content

The intervention is interactive, digitally delivered online in a fully automated form, and requires approximately 20 minutes to complete. Personalized feedback is given to the participants based on their responses to previous assessment and suggestions on how to respond to this feedback are provided. This interactivity imitates a face-to face "dialogue" with techniques from MI such as an empathic approach, rolling with resistance, aiming at creating a dissonance between actual and desired behaviour, raising self-

For peer review only - http://bmjopen.bmj.com/site/about/guidelines.xhtml

efficacy, and at the same time avoiding argumentation.[57] The intervention consists of three main components outlined below, and additional health related information.

Personalized feedback

The personalized feedback includes an estimation of the participants' blood-alcohol-concentration (BAC) and information on the associated risks concerning the participants' heaviest drinking episode during the past 30 days. The value will be based on a measure of Peak Drinking Quantity[93] and estimated using the Widmark-formula, which takes into account weight and sex.[94] The participants will receive graphed feedback regarding number of standard alcohol units per week that they think their peers consume (descriptive norms), as well as the participant's individual levels of consumption in relation to comparative data (actual drinking levels) from a reference group. Comparative data (AUDIT-C scores) will be taken from alcohol prevalence estimates found in a nationally representative sample of 16-25 year-olds undertaken by the Public Health Agency of Sweden. Comparative feedback will be available for drinking but not for substances other than alcohol.

Interactive MI-based exercises

The exercises provided in the current intervention build on the assumption that participants may hold certain levels of ambivalence about their current substance use, and that if they are willing to make a change they may not know how to, or may not be confident that they are able to.[41] Therefore, the intervention uses importance and confidence rulers with a short summary and feedback to encourage participants to reflect on personal reasons for change and explore personal strengths and ability to change. Furthermore, the programme provides a decisional balance to pick up and graphically illustrate potential levels of ambivalence by offering the participants a list of possible pros and cons regarding the decision to change their current substance use.[57 95] Participants are instructed to choose statements that apply to them and are presented with the resulting balance sheet of their personal comparative potential gains and losses.

For peer review only

Practical advice

The intervention focuses on raising self-efficacy for being able to avoid drinking in social situations, if desirable.[50] The participant will be asked to select three among 12 provided drinking situations that they consider most tempting and rank them. The situations are adapted from the adolescent version of the Drinking Refusal Self-efficacy Questionnaire (DRSEQ-RA).[96] According to the selection, a number of strategies will be offered for each of the selected drinking situations to provide participants with a tool kit necessary for engaging in and maintaining their behavioural goal.

Health related information connected to substance use

Finally, the intervention programme includes health related information associated with substance use. The information is provided optionally throughout the program behind "read more" buttons and also at the end of the programme. The information contains statements regarding risks connected to substance use that the participant can reflect on, as well as optional links for more information. There are also two FAQ-sections about alcohol and cannabis, respectively, as well as information about how different substances may affect the individual, both physically and mentally. Finally, the information also contains cases, were the participant can read about some typical young persons who have used substances, why they used and what negative effects they have noted, and why they chose to stop using.

The control condition

The control group will receive the same general health related information as the intervention group, i.e., the additional information which is connected to the intervention programme.

Statistical analysis

Randomisation-checks of baseline variables regarding alcohol and other substances use and psychological state will be conducted using multivariate analyses of variance MANOVA. To test the effectiveness of the intervention, we will assess whether participants in the intervention group to a greater extent report decreased substance use, sexual risk behaviours, perceived peer-pressure, and improved mental health after three and six months, respectively, compared to participants in the active control group. Data analyses will consist of comparing outcome measurements with regards to within-group and between-group differences according to the intention-to-treat (ITT) principle in the primary analysis, accounting for all included participants regardless of whether or not they completed follow-up assessments. We will also perform per protocol analyses. The reason for choosing ITT in the primary analysis is the ambition to

For peer review only - http://bmjopen.bmj.com/site/about/guidelines.xhtml

maintain the power of the study and also to avoid biased results due to selection of those completing all follow-ups, as they may have special characteristics not representative for the whole study group, which might influence study results. The main analysis of effectiveness will use mixed effects regression models, which can be applied to both continuous and categorical outcomes and also non-normally distributed outcomes. Also, mixed-effects regression models are robust to missing data in longitudinal studies.[97] In the current study, we assume a quite large dropout rate, based on previous research. For example, a meta-analysis comprising 17 studies of unsupported (fully automated) interventions for depression showed 74% dropout rate at post-treatment.[98] Moreover, we expect data missing at random (MAR), which can be handled using mixed effects regression models. Separate models will be run to test each outcome, i.e., alcohol consumption, binge drinking, frequency of alcohol consumption, amount of alcohol consumed a typical day when alcohol is consumed, average daily drinks per typical week, other substance use, mental health, sexual risk behaviours and perceived peer pressure at three- and six-month follow-ups. Effect size will be calculated for the primary outcome variable (AUDIT-C total score). Potential moderators, including perfectionism, openness to parents, help-seeking due to mental health problems, and socio-demographic and personal characteristics, (i.e. sex, age, residence, occupation, school performance (for students), and parents' education) will serve as covariates in analyses of intervention effects. If moderator effects are found, we will carry out post-hoc analyses stratified by the detected variable.

Patient and public involvement statement

Patients and the public were not involved in the design and planning of the study, except for persons at the age of the target group reading manuscripts for the revised version of WISEteens and also participating in a pilot-test.

ETHICS AND DISSEMINATION

The current study was approved by the Swedish Ethical Review Authority (no. 2019-03249) and registered 24/09/2019, pre-results, on IRCTN, ID: ISRCTN91048246. Any important protocol modifications will be reported to IRCTN. For inclusion, all participants must give informed consent online prior to participation in the study. In order to participate, the participants will need to state a username (which may be fictitious or a pseudonym) and an email address. All data collection will be done without collecting personal identification information, only personal e-mail addresses. The e-mail address will be used to connect data from the baseline measurement to the follow-up measurements. At a later stage, the

For peer review only - http://bmjopen.bmj.com/site/about/guidelines.xhtml

raw data file will be anonymized and each person assigned a number instead of the e-mail address. The data will be stored in line with routines for handling and storing research data at Karolinska Institutet. All data is handled confidentially and will not be forwarded to third parties. Participating in the present study means that the participants need to reflect on their alcohol consumption and other substance use. In addition, questions will be asked concerning personal circumstances, including the participants' mental health and family relationships. These issues may be perceived as somewhat unpleasant. However, in the information that potential participants receive, they are informed that participation is voluntary and that they at any time can end their participation without explaining why. In addition, there will be reference to other types of official support (web pages and telephone numbers), if the advertisement or participation leads to concern about own substance use or related problems. Any issues brought up by the participants during the study will be documented and handled properly. Moreover, the research team includes professionals, such as a psychiatrist and a nurse, with possibility to refer participants to health care clinics if needed. The project's basic hypothesis is that the intervention will have positive effects, with regard to alcohol and other substance use among young people with risk use, and potential benefit may include decreased or ceased risk behaviours. Overall, the benefit for the research persons is considered to exceed any risk of discomfort. Results will be disseminated in scientific peer reviewed journals, at conferences and via the media.

DISCUSSION OF STRENGTHS AND LIMITATIONS

The current study has a number of strengths. The protocol describes a two-arm double-blind RCT, considered to be the most robust experimental design, controlling for selection bias and participant allocation bias and with the possibility to make causal inferences. Moreover, an active control condition, blinded to the study condition, controls for expectation, detection and performance bias. The collection and analysis of information on potential moderators, allowing for control of these factors, facilitates the understanding of the possible effects. Thus, the present study will contribute to the literature on digital substance use prevention interventions among adolescents and young adults in several ways. The choice to study a digital intervention can from a public health perspective be regarded as positive. Digital brief interventions have several advantages over face-to-face approaches, e.g., the reduction of stigma around help-seeking for substance use and easy dissemination to large groups of people, which makes them cost-effective. Moreover, digital interventions have previously proven effective in addressing alcohol and other substance use in the general population and some studies have shown promising results also among adolescents and young adults.[99 100] The current intervention is well grounded in theory and incorporates elements of MI and social influence theory that has been shown to be effective in reducing problematic

For peer review only - http://bmjopen.bmj.com/site/about/guidelines.xhtml

substance use in prior research.[57] Importantly, there are also some limitations to the current study. One limitation concerns selection bias and thereby external validity, as recruitment requires either that individuals click on our ads at social media to be considered for inclusion, or that they attend upper secondary schools, or have been in contact with existing clinics, aware of the opportunity to participate in the study. Thus, our participants may have certain personality traits, or are especially prone to help-seeking compared to a broader audience. The exclusion of people not understanding Swedish is also a limitation with regard to generalizability.

STATEMENTS

Author contributions

PK, AKS, JG, and THE obtained funding for the study. PK, THE, and CS further developed/modified the intervention. THE designed the study with contribution from PK, AKS, JG, AHB, and, CS. PK wrote this paper. All authors commented on successive manuscript drafts and approved the final version of the manuscript.

Funding statement

The work was supported by the National Public Health Agency of Sweden (grant no. 02299-2016-6.2) and the Alcohol Research Council of the Swedish Alcohol Retailing Monopoly (grant no. 2018-0016). Author AHB was supported by the Swedish Research Council (grant no. K2012-61P-22131-01-6). The funding bodies had no role in study design, data collection, analysis, data interpretation or writing manuscripts.

Data statement

Collected data will be available from the Centre for Psychiatry Research, a collaboration between the Karolinska Institute and Region Stockholm, but restrictions apply to their availability, as they were used under ethical permission for the current study, and so are not publicly available. However, data are available from the authors upon reasonable request and with permission from the Centre for Psychiatry Research.

For peer review only - http://bmjopen.bmj.com/site/about/guidelines.xhtml

Competing interests

The authors declare no competing interests.

Acknowledgments

The authors would like to thank Dr. Silke Diestelkamp and Dr. Christiane Baldus for invaluable help in the process of building the Swedish version of the WISEteens programme.

For peer review only

REFERENCES

1. Dawson DA, Goldstein RB, Chou SP, et al. Age at first drink and the first incidence of adult-onset DSM-IV alcohol use disorders. *Alcoholism Clinical and Experimental Research* 2008;32(12):2149-60.
2. Hibell B, Guttormsson U, Ahlström S, et al. The 2007 ESPAD Report: Substance use among students in 35 European countries. ESPAD, The European School Survey Project on Alcohol and Other Drugs. Stockholm, Sweden, 2009.
3. Anderson P. Global alcohol policy and the alcohol industry. *Curr Opin Psychiatry* 2009;22(3):253-7. doi: 10.1097/YCO.0b013e328329ed75
4. Chambers RA, Taylor JR, Potenza MN. Developmental neurocircuitry of motivation in adolescence: a critical period of addiction vulnerability. *The American journal of psychiatry* 2003;160(6):1041-52. doi: 10.1176/appi.ajp.160.6.1041 [published Online First: 2003/06/05]
5. Barron S, White A, Swartzwelder HS, et al. Adolescent vulnerabilities to chronic alcohol or nicotine exposure: findings from rodent models. *Alcohol Clin Exp Res* 2005;29(9):1720-5. [published Online First: 2005/10/06]
6. Balogh KN, Mayes LC, Potenza MN. Risk-taking and decision-making in youth: relationships to addiction vulnerability. *Journal of behavioral addictions* 2013;2(1) doi: 10.1556/jba.2.2013.1.1 [published Online First: 2013/12/03]
7. Oetting ER, Beauvais F. Peer cluster theory: Drugs and the adolescent. *Journal of Counseling & Development* 1986;65(1):17-22. doi: 10.1002/j.1556-6676.1986.tb01219.x
8. Arria AM, Caldeira KM, Bugbee BA, et al. Marijuana use trajectories during college predict health outcomes nine years post-matriculation. *Drug and alcohol dependence* 2016;159:158-65. doi: 10.1016/j.drugalcdep.2015.12.009 [published Online First: 2015/12/23]
9. Van Der Vorst H, Vermulst AA, Meeus WH, et al. Identification and prediction of drinking trajectories in early and mid-adolescence. *Journal of clinical child and adolescent psychology : the official journal for the Society of Clinical Child and Adolescent Psychology, American Psychological Association, Division 53* 2009;38(3):329-41. doi: 10.1080/15374410902851648 [published Online First: 2009/05/14]
10. R M, E K, P M, et al. Impact of alcohol consumption on young people: A systematic review of published reviews. Newcastle University, 2009.
11. Hicks RD, Bemis Batzer G, Bemis Batzer W, et al. Psychiatric, Developmental, and Adolescent Medicine Issues in Adolescent Substance Use and Abuse. *Adolesc Med* 1993;4(2):453-68.
12. White V, Azar D, Faulkner A, et al. Adolescents' alcohol use and strength of policy relating to youth access, trading hours and driving under the influence: findings from Australia. *Addiction* 2018;113(6):1030-42. doi: 10.1111/add.14164
13. Arendt M, Rosenberg R, Foldager L, et al. Cannabis-induced psychosis and subsequent schizophrenia-spectrum disorders: follow-up study of 535 incident cases. *The British journal of psychiatry : the journal of mental science* 2005;187:510-5. doi: 10.1192/bjp.187.6.510 [published Online First: 2005/12/02]
14. Flory K, Lynam D, Milich R, et al. Early adolescent through young adult alcohol and marijuana use trajectories: early predictors, young adult outcomes, and predictive utility. *Development and psychopathology* 2004;16(1):193-213. [published Online First: 2004/04/30]
15. Nilses C, Blom H, Heimer G, et al. 40 procent av gymnasieelever riskbrukar alkohol. *Läkartidningen* 2011;34(108):1156-559.
16. Bonomo Y, Coffey C, Wolfe R, et al. Adverse outcomes of alcohol use in adolescents. *Addiction* 2001;96(10):1485-96.
17. Guttmannova K, Hill KG, Bailey JA, et al. Examining explanatory mechanisms of the effects of early alcohol use on young adult alcohol dependence. *J Stud Alcohol Drugs* 2012;73(3):379-90.

For peer review only - http://bmjopen.bmj.com/site/about/guidelines.xhtml

18. Miller JW, Naimi TS, Brewer RD, et al. Binge drinking and associated health risk behaviors among high school students. *Pediatrics* 2007;119(1):76-85.
19. Chick CF, Reyna VF. A fuzzy trace theory of adolescent risk taking: Beyond self-control and sensation seeking. In: V. Reyna SC, M. Dougherty, & J. Confrey (Eds.), ed. The Adolescent Brain Washington, DC: American Psychological Association. 2012.
20. Burdzovic Andreas J, Lauritzen G, Nordfjærn T. Co-occurrence between mental distress and poly-drug use: A ten year prospective study of patients from substance abuse treatment. *Addict Behav* 2015;48:71-78. doi: https://doi.org/10.1016/j.addbeh.2015.05.001
21. Hodgins S, Tengström A, Bylin S, et al. Consulting for substance abuse: Mental disorders among adolescents and their parents. *Nordic Journal of Psychiatry* 2007;61(5):379-86. doi: 10.1080/08039480701643423
22. Swift W, Coffey C, Carlin JB, et al. Adolescent cannabis users at 24 years: trajectories to regular weekly use and dependence in young adulthood. *Addiction* 2008;103(8):1361-70. doi: 10.1111/j.1360-0443.2008.02246.x [published Online First: 2008/10/16]
23. Von Sydow K, Lieb R, Pfister H, et al. What predicts incident use of cannabis and progression to abuse and dependence? A 4-year prospective examination of risk factors in a community sample of adolescents and young adults. *Drug and Alcohol Dependence* 2002;68:49–64.
24. Låftman SB, Almquist YB, Östberg V. Students' accounts of school-performance stress: a qualitative analysis of a high-achieving setting in Stockholm, Sweden. *Journal of Youth Studies* 2013;16(7):932-49. doi: 10.1080/13676261.2013.780126
25. Hiltunen L. Lagom perfekt. Erfarenheter av ohälsa bland unga tjejer och killar. Linnéuniversitetet, 2017.
26. Luthar SS, Becker BE. Privileged but pressured? A study of affluent youth. *Child development* 2002;73(5):1593-610. [published Online First: 2002/10/04]
27. Ashby Wills T, Yaeger AM. Family Factors and Adolescent Substance Use: Models and Mechanisms. *Current Directions in Psychological Science* 2003;12(6):222-26. doi: 10.1046/j.0963-7214.2003.01266.x
28. Wills TA, Cleary SD. How are social support effects mediated? A test with parental support and adolescent substance use. *Journal of Personality and Social Psychology* 1996;71(5):937-52. doi: 10.1037/0022-3514.71.5.937
29. Guttormsson U, Gröndal M. Befolkningens självrapporterade alkoholvanor 2004-2017 Stockholm: CAN, 2018.
30. Lees B, Mewton L, Stapinski L, et al. Binge drinking in young people: protocol for a systematic review of neuropsychological, neurophysiological and neuroimaging studies. *BMJ Open* 2018;8(7):e023629. doi: 10.1136/bmjopen-2018-023629
31. European Union Drugs Strategy 2013–2020, 2013.
32. Zetterqvist ME. Skolelevers drogvanor 2018. Stockholm: Centralförbundet för alkohol- och narkotikaupplysning (CAN) 2018.
33. Foundation TSI. Svenskarna och internet 2018 (The Swedes and the Internet 2018) (In Swedish) 2018 [Available from: https://2018.svenskarnaochinternet.se/allmant-om-internetutvecklingen/ accessed 2019-06-25.
34. Murray E. Web-Based Interventions for Behavior Change and Self-Management: Potential, Pitfalls, and Progress. *Med 20* 2012;1(2):e3. doi: 10.2196/med20.1741
35. Riper H, Blankers M, Hadiwijaya H, et al. Effectiveness of Guided and Unguided Low-Intensity Internet Interventions for Adult Alcohol Misuse: A Meta-Analysis. *PLOS ONE* 2014;9(6):e99912. doi: 10.1371/journal.pone.0099912

For peer review only - http://bmjopen.bmj.com/site/about/guidelines.xhtml

36. Spijkerman R, Roek AEM, Vermulst A, et al. Effectiveness of a Web-Based Brief Alcohol Intervention and Added Value of Normative Feedback in Reducing Underage Drinking: A Randomized Controlled Trial. *J Med Internet Res* 2010;12(5):e65. doi: 10.2196/jmir.1465
37. Sinadinovic K, Berman AH, Hasson D, et al. Internet-based assessment and self-monitoring of problematic alcohol and drug use. *Addictive behaviors* 2010;35(5):464-70. doi: 10.1016/j.addbeh.2009.12.021 [published Online First: 2010/01/23]
38. Arnaud N, Baldus C, Elgan TH, et al. Effectiveness of a Web-Based Screening and Fully Automated Brief Motivational Intervention for Adolescent Substance Use: A Randomized Controlled Trial. *J Med Internet Res* 2016;18(5):e103. doi: 10.2196/jmir.4643
39. Arnaud N, Bröning S, Drechsel M, et al. Web-based screening and brief intervention for poly-drug use among teenagers: study protocol of a multicentre two-arm randomized controlled trial. *BMC Public Health* 2012;12:826. doi: 10.1186/1471-2458-12-826 [published Online First: 2012/09/28]
40. Arnaud N, Baldus C, Elgán TH, et al. Moderators of Outcome in a Web-Based Substance Use Intervention for Adolescents. *SUCHT* 2015;61(6):377-87. doi: 10.1024/0939-5911.a000397
41. Miller WR, Rollnick S. Motivational Interviewing: Helping People Change. 3rd ed. New York: The Guilford Press 2013.
42. Ajzen I. The Theory of Planned Behavior. *Organizational Behavior and Human Decision Processes* 1991;50(2):179-211.
43. Bandura A. Social foundations of thought and action: A social cognitive theory. NJ: Prentice-Hall: Englewood Cliffs 1986.
44. Voogt CV, Poelen EA, Kleinjan M, et al. The effectiveness of the 'what do you drink' web-based brief alcohol intervention in reducing heavy drinking among students: a two-arm parallel group randomized controlled trial. *Alcohol Alcohol* 2013;48(3):312-21. doi: 10.1093/alcalc/ags133 [published Online First: 2013/01/11]
45. Wood MD, Fairlie AM, Fernandez AC, et al. Brief Motivational and Parent Interventions for College Students: A Randomized Factorial Study. *J Consult Clin Psych* 2010;78(3):349-61. doi: Doi 10.1037/A0019166
46. Carey KB, Scott-Sheldon LAJ, Elliott JC, et al. Computer-delivered interventions to reduce college student drinking: a meta-analysis. *Addiction (Abingdon, England)* 2009;104(11):1807-19. doi: 10.1111/j.1360-0443.2009.02691.x [published Online First: 2009/09/10]
47. Moreira MT, Smith LA, Foxcroft D. Social norms interventions to reduce alcohol misuse in university or college students. *The Cochrane database of systematic reviews* 2009(3):Cd006748. doi: 10.1002/14651858.CD006748.pub2 [published Online First: 2009/07/10]
48. Doumas DM, Hannah E. Preventing high-risk drinking in youth in the workplace: a web-based normative feedback program. *Journal of substance abuse treatment* 2008;34(3):263-71. doi: 10.1016/j.jsat.2007.04.006 [published Online First: 2007/06/30]
49. DONALDSON SI, SUSSMAN S, MacKINNON DP, et al. Drug Abuse Prevention Programming:Do We Know What Content Works? *American Behavioral Scientist* 1996;39(7):868-83. doi: 10.1177/0002764296039007008
50. Voogt CV, Poelen EAP, Kleinjan M, et al. Targeting young drinkers online: the effectiveness of a web-based brief alcohol intervention in reducing heavy drinking among college students: study protocol of a two-arm parallel group randomized controlled trial. *BMC Public Health* 2011;11(1):231. doi: 10.1186/1471-2458-11-231
51. Frost H, Campbell P, Maxwell M, et al. Effectiveness of Motivational Interviewing on adult behaviour change in health and social care settings: A systematic review of reviews. *PloS one* 2018;13(10):e0204890-e90. doi: 10.1371/journal.pone.0204890

For peer review only - http://bmjopen.bmj.com/site/about/guidelines.xhtml

52. Kohler S, Hofmann A. Can motivational interviewing in emergency care reduce alcohol consumption in young people? A systematic review and meta-analysis. *Alcohol Alcohol* 2015;50(2):107-17. doi: 10.1093/alcalc/agu098 [published Online First: 2015/01/08]
53. Li L, Zhu S, Tse N, et al. Effectiveness of motivational interviewing to reduce illicit drug use in adolescents: a systematic review and meta-analysis. *Addiction* 2016;111(5):795-805. doi: 10.1111/add.13285 [published Online First: 2015/12/22]
54. Tanner-Smith EE, Lipsey MW. Brief alcohol interventions for adolescents and young adults: a systematic review and meta-analysis. *Journal of substance abuse treatment* 2015;51:1-18. doi: 10.1016/j.jsat.2014.09.001 [published Online First: 2014/10/11]
55. Bahner J, Stenqvist K. Motivational Interviewing as Evidence-Based Practice? An Example from Sexual Risk Reduction Interventions Targeting Adolescents and Young Adults. *Sexuality Research and Social Policy* 2019 doi: 10.1007/s13178-019-00388-y
56. Gates P, Norberg M, Copeland J, et al. Randomized controlled trial of a novel cannabis use intervention delivered by telephone. *Addiction (Abingdon, England)* 2012;107 doi: 10.1111/j.1360-0443.2012.03953.x
57. Berman AH, Beckman M, Lindqvist H. Motivational Interviewing interventions. In: Hagger MS, Cameron LD, Hamilton K, et al., eds. Handbook of Behavior Change. New York, NY: Cambridge University Press. In press, 2019.
58. Bewick BM, Trusler K, Barkham M, et al. The effectiveness of web-based interventions designed to decrease alcohol consumption — A systematic review. *Preventive Medicine* 2008;47(1):17-26. doi: https://doi.org/10.1016/j.ypmed.2008.01.005
59. White A, Kavanagh D, Stallman H, et al. Online Alcohol Interventions: A Systematic Review. *J Med Internet Res* 2010;12(5):e62. doi: 10.2196/jmir.1479
60. Toumbourou JW, Stockwell T, Neighbors C, et al. Interventions to reduce harm associated with adolescent substance use. *Lancet (London, England)* 2007;369(9570):1391-401. doi: 10.1016/S0140-6736(07)60369-9
61. Natarajan M, Kaner E. Brief alcohol interventions in young people: evidence and applicability. I E. Gilvarry & P. McArdle (redaktörer), Alcohol, drugs and young people: clinical approaches. Newcastle upon Tyne: Mac Keith Press 2007.
62. Sinadinovic K, Wennberg P, Berman AH. Internet-based screening and brief intervention for illicit drug users: a randomized controlled trial with 12-month follow-up. *J Stud Alcohol Drugs* 2014;75(2):313-8. [published Online First: 2014/03/22]
63. Tait RJ, Spijkerman R, Riper H. Internet and computer based interventions for cannabis use: A meta-analysis. *Drug and Alcohol Dependence* 2013;133(2):295-304. doi: https://doi.org/10.1016/j.drugalcdep.2013.05.012
64. Cavanagh K, Millings A. (Inter)personal Computing: The Role of the Therapeutic Relationship in E-mental Health. *Journal of Contemporary Psychotherapy* 2013;43(4):197-206. doi: 10.1007/s10879-013-9242-z
65. Webb TL, Joseph J, Yardley L, et al. Using the internet to promote health behavior change: a systematic review and meta-analysis of the impact of theoretical basis, use of behavior change techniques, and mode of delivery on efficacy. *J Med Internet Res* 2010;12(1):e4. doi: 10.2196/jmir.1376 [published Online First: 2010/02/19]
66. Lee CM, Neighbors C, Kilmer JR, et al. A brief, web-based personalized feedback selective intervention for college student marijuana use: a randomized clinical trial. *Psychol Addict Behav* 2010;24(2):265-73. doi: 10.1037/a0018859 [published Online First: 2010/06/23]
67. Rooke S, Copeland J, Norberg M, et al. Effectiveness of a self-guided web-based cannabis treatment program: randomized controlled trial. *Journal of medical Internet research* 2013;15(2):e26-e26. doi: 10.2196/jmir.2256

For peer review only - http://bmjopen.bmj.com/site/about/guidelines.xhtml

68. Tonnesen H, H S, Pedersen B. Review: Web-based brief interventions for young adolescent alcohol and drug abusers - a systematic review2013.
69. Chan A-W, Tetzlaff JM, Altman DG, et al. SPIRIT 2013 statement: defining standard protocol items for clinical trials. *Ann Intern Med* 2013;158(3):200-07. doi: 10.7326/0003-4819-158-3-201302050-00583
70. Cohen J. A power primer. *Psychological bulletin* 1992;112(1):155-9. [published Online First: 1992/07/01]
71. Riper H, Hoogendoorn A, Cuijpers P, et al. Effectiveness and treatment moderators of internet interventions for adult problem drinking: An individual patient data meta-analysis of 19 randomised controlled trials. *PLOS Medicine* 2018;15(12):e1002714. doi: 10.1371/journal.pmed.1002714
72. Knight JR, Shrier LA, Bravender TD, et al. A new brief screen for adolescent substance abuse. *Arch Pediatr Adolesc Med* 1999;153(6):591-6. [published Online First: 1999/06/05]
73. Levy S, Sherritt L, Harris SK, et al. Test-retest reliability of adolescents' self-report of substance use. *Alcohol Clin Exp Res* 2004;28(8):1236-41. [published Online First: 2004/08/20]
74. Knight JR, Sherritt L, Harris SK, et al. Validity of brief alcohol screening tests among adolescents: a comparison of the AUDIT, POSIT, CAGE, and CRAFFT. *Alcohol Clin Exp Res* 2003;27(1):67-73. doi: 10.1097/01.ALC.0000046598.59317.3A [published Online First: 2003/01/25]
75. Kallmen H, Berman AH, Jayaram-Lindstrom N, et al. Psychometric Properties of the AUDIT, AUDIT-C, CRAFFT and ASSIST-Y among Swedish Adolescents. *European addiction research* 2019;25(2):68-77. doi: 10.1159/000496741 [published Online First: 2019/02/07]
76. Saunders JB, Aasland OG, Babor TF, et al. Development of the Alcohol Use Disorders Identification Test (AUDIT): WHO Collaborative Project on Early Detection of Persons with Harmful Alcohol Consumption--II. *Addiction* 1993;88(6):791-804.
77. Rumpf HJ, Wohlert T, Freyer-Adam J, et al. Screening questionnaires for problem drinking in adolescents: performance of AUDIT, AUDIT-C, CRAFFT and POSIT. *European addiction research* 2013;19(3):121-7. doi: 10.1159/000342331 [published Online First: 2012/11/28]
78. Collins RL, Parks GA, Marlatt GA. Social determinants of alcohol consumption: the effects of social interaction and model status on the self-administration of alcohol. *J Consult Clin Psychol* 1985;53(2):189-200. [published Online First: 1985/04/01]
79. Kivlahan DR, Marlatt GA, Fromme K, et al. Secondary prevention with college drinkers: Evaluation of an alcohol skills training program. *J Consult Clin Psych* 1990;58(6):805-10. doi: 10.1037/0022-006X.58.6.805
80. Higgins-Biddle JC, Babor TF. A review of the Alcohol Use Disorders Identification Test (AUDIT), AUDIT-C, and USAUDIT for screening in the United States: Past issues and future directions. *The American journal of drug and alcohol abuse* 2018;44(6):578-86. doi: 10.1080/00952990.2018.1456545 [published Online First: 2018/05/03]
81. Berman AH, Bergman H, Palmstierna T, et al. Evaluation of the Drug Use Disorders Identification Test (DUDIT) in criminal justice and detoxification settings and in a Swedish population sample. *European addiction research* 2005;11(1):22-31. doi: 81413 [pii] 10.1159/000081413 [published Online First: 2004/12/21]
82. Hildebrand M. The psychometric properties of the Drug Use Disorders Identification Test (DUDIT): A review of recent research. *Journal of substance abuse treatment* 2015 a;53:52-59.
83. Sinadinovic K, Wennberg P, Berman AH. Population screening of risky alcohol and drug use via Internet and Interactive Voice Response (IVR): A feasibility and psychometric study in a random sample. *Drug and Alcohol Dependence* 2011;114(1):55-60. doi: https://doi.org/10.1016/j.drugalcdep.2010.09.004

For peer review only

For peer review only - http://bmjopen.bmj.com/site/about/guidelines.xhtml

84. Topp CW, Ostergaard SD, Sondergaard S, et al. The WHO-5 Well-Being Index: a systematic review of the literature. *Psychotherapy and psychosomatics* 2015;84(3):167-76. doi: 10.1159/000376585 [published Online First: 2015/04/04]
85. Love J, Andersson L, Moore CD, et al. Psychometric analysis of the Swedish translation of the WHO well-being index. *Quality of life research : an international journal of quality of life aspects of treatment, care and rehabilitation* 2014;23(1):293-7. doi: 10.1007/s11136-013-0447-0 [published Online First: 2013/06/08]
86. Strandberg AK, Skoglund C, Gripenberg J, et al. Alcohol and illicit drug consumption and the association with risky sexual behavior among Swedish youths visiting youth health clinics. *Nordic Studies on Alcohol and Drugs* Accepted
87. Brown BB, Clasen DR, Eicher SA. Perceptions of peer pressure, peer conformity dispositions, and self-reported behavior among adolescents. *Developmental Psychology* 1986;22(4):521-30. doi: 10.1037/0012-1649.22.4.521
88. Frost RO, Marten P, Lahart C, et al. The dimensions of perfectionism. *Cognitive Therapy and Research* 1990;14(5):449-68. doi: 10.1007/BF01172967
89. Rozental A, Shafran R, Wade T, et al. A randomized controlled trial of Internet-Based Cognitive Behavior Therapy for perfectionism including an investigation of outcome predictors. *Behaviour research and therapy* 2017;95:79-86. doi: 10.1016/j.brat.2017.05.015 [published Online First: 2017/06/07]
90. Stattin H, Kerr M. Parental monitoring: a reinterpretation. *Child development* 2000;71(4):1072-85. [published Online First: 2000/10/04]
91. Salari R, Thorell LB. Parental monitoring in late adolescence: Relations to ADHD symptoms and longitudinal predictors. *Journal of Adolescence* 2015;40:24.
92. Kerr M, Stattin H. What parents know, how they know it, and several forms of adolescent adjustment: further support for a reinterpretation of monitoring. *Dev Psychol* 2000;36(3):366-80. [published Online First: 2000/06/01]
93. Marlatt GA, Baer JS, Kivlahan DR, et al. Screening and brief intervention for high-risk college student drinkers: Results from a 2-year follow-up assessment. *J Consult Clin Psych* 1998;66(4):604-15.
94. Widmark E. Die theoretischen Grundlagen und die praktische Verwendbarkeit der gerichtlich-medizinischen Alkoholbestimmung. Berlin Wien: Urban und Schwarzenberg 1932.
95. Babor T, Higgins-Biddle J. Brief Intervention For Hazardous and Harmful Drinking: A Manual for use in Primary Care: World health Organization 2001.
96. Young RM, Hasking PA, Oei TP, et al. Validation of the Drinking Refusal Self-Efficacy Questionnaire--Revised in an Adolescent Sample (DRSEQ-RA). *Addictive behaviors* 2007;32(4):862-8. doi: 10.1016/j.addbeh.2006.07.001 [published Online First: 2006/08/22]
97. Gibbons RD, Hedeker D, DuToit S. Advances in analysis of longitudinal data. *Annu Rev Clin Psychol* 2010;6:79-107. doi: 10.1146/annurev.clinpsy.032408.153550
98. Richards D, Richardson T. Computer-based psychological treatments for depression: a systematic review and meta-analysis. *Clin Psychol Rev* 2012;32(4):329-42. doi: 10.1016/j.cpr.2012.02.004 [published Online First: 2012/02/28]
99. Cole HA, Prassel HB, Carlson CR. A Meta-Analysis of Computer-Delivered Drinking Interventions for College Students: A Comprehensive Review of Studies From 2010 to 2016. *J Stud Alcohol Drugs* 2018;79(5):686-96. [published Online First: 2018/11/14]
100. Berman AH, Gajecki M, Sinadinovic K, et al. Mobile Interventions Targeting Risky Drinking Among University Students: A Review. *Curr Addict Rep* 2016;3:166-74. doi: 10.1007/s40429-016-0099-6 [published Online First: 2016/04/05]

For peer review only - http://bmjopen.bmj.com/site/about/guidelines.xhtml

FIGURE LEGENDS

Figure 1 Study design

For peer review only

For peer review only - http://bmjopen.bmj.com/site/about/guidelines.xhtml

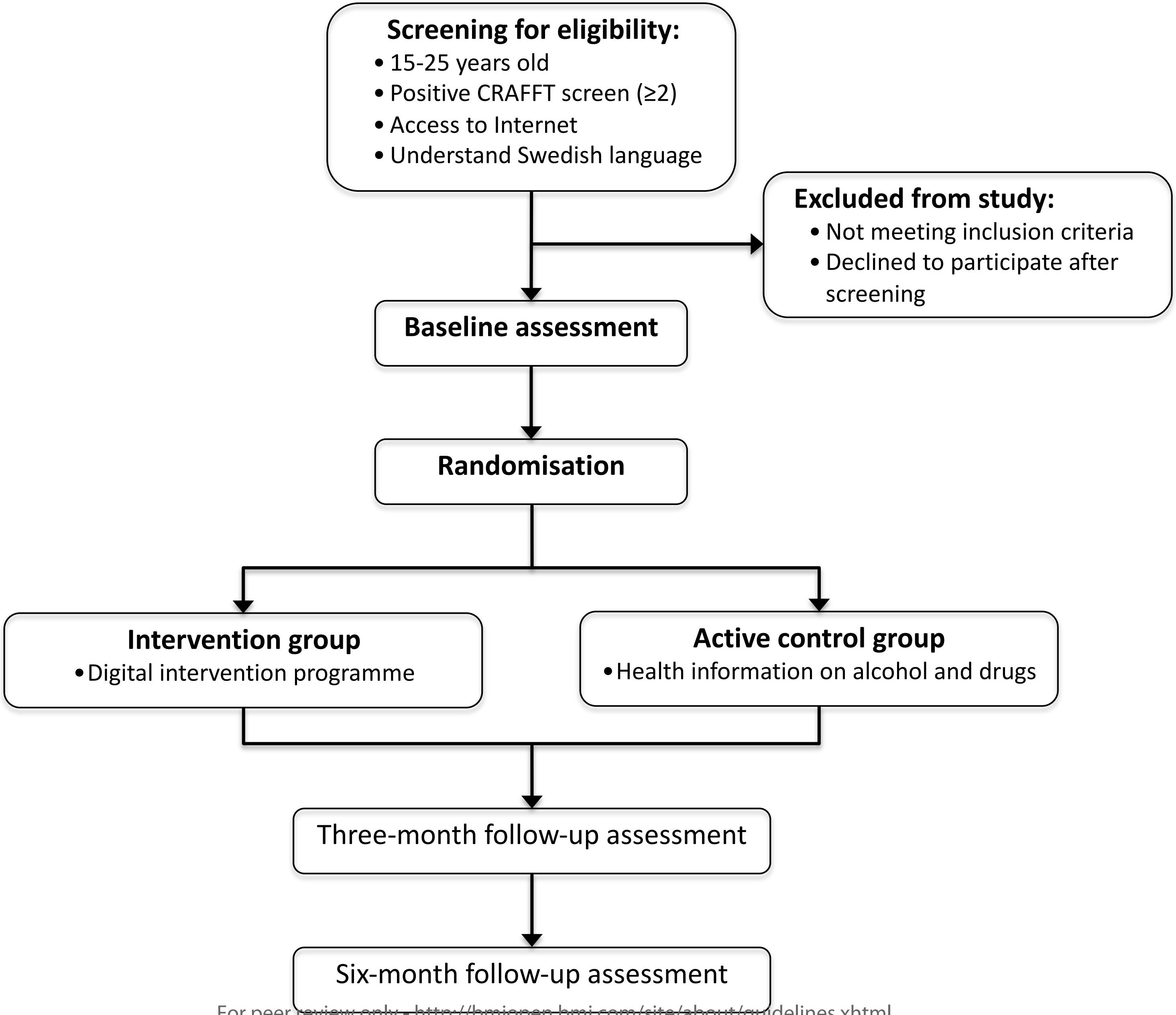
Screening for eligibility:
• 15-25 years old
• Positive CRAFFT screen (≥2)
• Access to Internet
• Understand Swedish language
Excluded from study:
• Not meeting inclusion criteria
• Declined to participate after screening
Baseline assessment
Randomisation
Intervention group
• Digital intervention programme
Active control group
• Health information on alcohol and drugs
Three-month follow-up assessment
Six-month follow-up assessment

CONSORT 2010 checklist of information to include when reporting a randomised trial*

Section/Topic	Item No	Checklist item	Reported on page No
**Title and abstract**			
	1a	Identification as a randomised trial in the title	Title page, 1
	1b	Structured summary of trial design, methods, results, and conclusions (for specific guidance see CONSORT for abstracts)	1-2
**Introduction**			
Background and objectives	2a	Scientific background and explanation of rationale	3-6
	2b	Specific objectives or hypotheses	6
**Methods**			
Trial design	3a	Description of trial design (such as parallel, factorial) including allocation ratio	6
	3b	Important changes to methods after trial commencement (such as eligibility criteria), with reasons	none
Participants	4a	Eligibility criteria for participants	7
	4b	Settings and locations where the data were collected	6-7
Interventions	5	The interventions for each group with sufficient details to allow replication, including how and when they were actually administered	9-10
Outcomes	6a	Completely defined pre-specified primary and secondary outcome measures, including how and when they were assessed	7-8
	6b	Any changes to trial outcomes after the trial commenced, with reasons	none
Sample size	7a	How sample size was determined	6
	7b	When applicable, explanation of any interim analyses and stopping guidelines	none
Randomisation:			9
Sequence generation	8a	Method used to generate the random allocation sequence	
	8b	Type of randomisation; details of any restriction (such as blocking and block size)	9
Allocation concealment mechanism	9	Mechanism used to implement the random allocation sequence (such as sequentially numbered containers), describing any steps taken to conceal the sequence until interventions were assigned	9
Implementation	10	Who generated the random allocation sequence, who enrolled participants, and who assigned participants to interventions	9
Blinding	11a	If done, who was blinded after assignment to interventions (for example, participants, care providers, those	9

For peer review only

		assessing outcomes) and how	
	11b	If relevant, description of the similarity of interventions	9-10
Statistical methods	12a	Statistical methods used to compare groups for primary and secondary outcomes	11
	12b	Methods for additional analyses, such as subgroup analyses and adjusted analyses	11
**Results**			
Participant flow (a diagram is strongly recommended)	13a	For each group, the numbers of participants who were randomly assigned, received intended treatment, and were analysed for the primary outcome	6, fig 1
	13b	For each group, losses and exclusions after randomisation, together with reasons	none
Recruitment	14a	Dates defining the periods of recruitment and follow-up	6-7
	14b	Why the trial ended or was stopped	not
Baseline data	15	A table showing baseline demographic and clinical characteristics for each group	none
Numbers analysed	16	For each group, number of participants (denominator) included in each analysis and whether the analysis was by original assigned groups	none
Outcomes and estimation	17a	For each primary and secondary outcome, results for each group, and the estimated effect size and its precision (such as 95% confidence interval)	none
	17b	For binary outcomes, presentation of both absolute and relative effect sizes is recommended	none
Ancillary analyses	18	Results of any other analyses performed, including subgroup analyses and adjusted analyses, distinguishing pre-specified from exploratory	none
Harms	19	All important harms or unintended effects in each group (for specific guidance see CONSORT for harms)	none
**Discussion**			
Limitations	20	Trial limitations, addressing sources of potential bias, imprecision, and, if relevant, multiplicity of analyses	12-13
Generalisability	21	Generalisability (external validity, applicability) of the trial findings	12-13
Interpretation	22	Interpretation consistent with results, balancing benefits and harms, and considering other relevant evidence	11-13
**Other information**			
Registration	23	Registration number and name of trial registry	1, 11
Protocol	24	Where the full trial protocol can be accessed, if available	11
Funding	25	Sources of funding and other support (such as supply of drugs), role of funders	13

*We strongly recommend reading this statement in conjunction with the CONSORT 2010 Explanation and Elaboration for important clarifications on all the items. If relevant, we also recommend reading CONSORT extensions for cluster randomised trials, non-inferiority and equivalence trials, non-pharmacological treatments, herbal interventions, and pragmatic trials. Additional extensions are forthcoming: for those and for up to date references relevant to this checklist, see www.consort-statement.org.

For peer review only - http://bmjopen.bmj.com/site/about/guidelines.xhtml
For peer review only
